# The Trithorax group protein ASH1 requires a combination of BAH domain and AT hooks, but not the SET domain, for mitotic chromatin binding and survival

**DOI:** 10.1007/s00412-021-00762-z

**Published:** 2021-07-31

**Authors:** Philipp A. Steffen, Christina Altmutter, Eva Dworschak, Sini Junttila, Attila Gyenesei, Xinzhou Zhu, Tobias Kockmann, Leonie Ringrose

**Affiliations:** 1 Institute of Molecular Biotechnology (IMBA), Dr. Bohr-Gasse 3, 1030 Vienna, Austria; 2 Vienna Biocenter Core Facilities GmbH (VBCF), Dr. Bohr-Gasse 3, 1030 Vienna, Austria; 3 Department of Biosystems Science and Engineering, ETH Zürich, Mattenstrasse 26, 4058 Basel, Switzerland; 4 Insistute of Biology, Humboldt-Universität Zu Berlin, Philippstrasse 13, Haus 22, 10115 Berlin, Germany

**Keywords:** ASH1, Trithorax, Chromatin, *Drosophila*, Mitosis

## Abstract

The *Drosophila* Trithorax group (TrxG) protein ASH1 remains associated with mitotic chromatin through mechanisms that are poorly understood. ASH1 dimethylates histone H3 at lysine 36 via its SET domain. Here, we identify domains of the TrxG protein ASH1 that are required for mitotic chromatin attachment in living *Drosophila*. Quantitative live imaging demonstrates that ASH1 requires AT hooks and the BAH domain but not the SET domain for full chromatin binding in metaphase, and that none of these domains are essential for interphase binding. Genetic experiments show that disruptions of the AT hooks and the BAH domain together, but not deletion of the SET domain alone, are lethal. Transcriptional profiling demonstrates that intact ASH1 AT hooks and the BAH domain are required to maintain expression levels of a specific set of genes, including several involved in cell identity and survival. This study identifies in vivo roles for specific ASH1 domains in mitotic binding, gene regulation, and survival that are distinct from its functions as a histone methyltransferase.

**Supplementary Information:**

The online version contains supplementary material available at 10.1007/s00412-021-00762-z.

## Introduction

During mitosis, chromatin undergoes profound structural changes. The interphase chromatin fibre is compacted over 300-fold, accompanied by extensive changes in DNA topology and a tenfold increase in the occurrence of single-stranded DNA (Belmont 2006; Juan et al. 1996; Liang et al. 2015; Michelotti et al. 1997). Histone acetylation and deacetylation cease, and core and linker histones become heavily phosphorylated (Gottesfeld and Forbes 1997; Kruhlak et al. 2001; Sawicka and Seiser 2012). In addition, RNA polymerases and many transcription factors and chromatin regulators disengage from chromatin during mitosis, and transcription is actively and globally repressed (Martínez-Balbás et al. 1995; Parsons and Spencer 1997; Spencer et al. 2000). However, despite these extensive structural rearrangements, mitotic chromatin is not inert. Several genes maintain a low level of transcription (Palozola et al. 2019), and not all regulatory proteins dissociate (Chen et al. 2005; Kadauke and Blobel 2013). Indeed, proteomic analysis has identified approximately 4000 proteins in isolated vertebrate mitotic chromosomes (Ohta et al. 2010). Proteins that remain bound to mitotic chromatin include centromeric proteins (Ohta et al. 2010), transcription factors (Chen et al. 2005; Kadauke and Blobel 2013) and several Polycomb and Trithorax group proteins (Blobel et al. 2009; Dey et al. 2009; Steffen et al. 2013; Zhao et al. 2011).

The Polycomb (PcG) and Trithorax (TrxG) groups of proteins work antagonistically to maintain active (TrxG) and silent (PcG) states of gene expression and can do so over many cell generations in the absence of the transcription factors that initially determined the gene expression state (Steffen and Ringrose 2014). This epigenetic maintenance is thought to involve both the maintenance of histone modifications and the direct binding of the PcG and TrxG proteins to replicating and mitotic chromatin (Alabert et al. 2015; Follmer et al. 2012; Francis et al. 2009; Lengsfeld et al. 2012; Lo et al. 2012; Petruk et al. 2012; Steffen et al. 2013) reviewed in Francis (2009), Steffen and Ringrose (2014) and Bellec et al. (2018). The polycomb group proteins for which mitotic chromatin attachment has been studied all dissociate completely or partially from mitotic chromatin (Buchenau et al. 1998; Dietzel et al. 1999; Follmer et al. 2012; Fonseca et al. 2012; Steffen et al. 2013). In contrast to the PcG proteins, several TrxG proteins remain extensively associated with mitotic chromatin (Blobel et al. 2009; Dey et al. 2009; Steffen et al. 2013; Zhao et al. 2011), reviewed in Steffen and Ringrose (2014). Mitotic binding of the mammalian TrxG proteins MLL and BRD4 has been shown to be required for correct post-mitotic gene activation of specific genes in cultured cells indicating that “mitotic bookmarking” by TrxG proteins may be an essential component of epigenetic memory of active gene expression states (Blobel et al. 2009; Dey et al. 2009; Zhao et al. 2011). Mitotic bookmarking of active transcriptional states has also been observed in *Drosophila* embryos (Bellec et al. 2018; Ferraro et al. 2016), but a role for *Drosophila* TrxG proteins has not been directly demonstrated.

We have previously shown that the *Drosophila* TrxG protein ASH1 remains bound to chromatin throughout mitosis (Steffen et al. 2013). ASH1 is a histone methyltransferase, whose SET domain dimethylates lysine 36 on histone H3 (An et al. 2011; Dorighi and Tamkun 2013; Gregory et al. 2007; Tanaka et al. 2007). Unlike several other TrxG proteins, which have a general role in transcriptional activation (Kingston and Tamkun 2014; Smith et al. 2004), ASH1 is thought to be required specifically at PcG target genes to counteract PcG-mediated silencing (Dorighi and Tamkun 2013; Klymenko and Müller 2004; Papp and Müller 2006; Rozovskaia et al. 1999). *ash1* null alleles are homozygous lethal at late pupal stages (Schmäling et al. 2018). Surviving adults and hypomorphic alleles show homeotic transformations attributable to loss of maintenance of *Hox* gene activation (Kingston and Tamkun 2014; Schmäling et al. 2018; Shearn^1989^; Tripoulas et al. 1996, 1994). Many additional targets for ASH1 beyond the *Hox* genes have been identified, which may be targeted at different developmental times or in different tissues (Beltran et al. 2007; Kockmann et al. 2013; Schmäling et al. 2018; Schwartz et al. 2010; Tripoulas et al. 1996). Interestingly, recent studies have shown that ASH1 histone methyltransferase activity is not essential for survival (Schmäling et al. 2018) or for counteracting PcG repression (Dorafshan et al. 2019a, b). These studies indicate that additional properties of ASH1, independent of its histone methyltransferase activity, are required for its full function. ASH1 binds mitotic chromatin (Steffen et al. 2013), but how this mitotic binding is mediated, and whether it occurs through similar mechanisms to interphase binding, is unknown.

Here, we use quantitative live imaging in *Drosophila* embryos to identify domains of the TrxG protein ASH1 that are required for binding to chromatin during mitosis and interphase. We show that the AT hooks and the BAH domain but not the SET domain are required for mitotic binding, and that none of these domains are essential for binding in interphase. Addressing the role of these domains during development in living animals, we show that disruption of the BAH domain and the AT hooks together causes complete lethality. In contrast, animals in which ASH1 lacks the SET domain are able to survive to adulthood. Thus, the domains of ASH1 that are essential for mitotic chromatin binding are also required for survival. This study identifies roles in living animals for specific ASH1 domains in mitotic binding, gene regulation and survival that are distinct from its functions as a histone methyltransferase.

## Results

### The ASH1 AT hooks and the BAH domain but not the SET domain are required for chromatin binding in metaphase

**Fig. 1:**
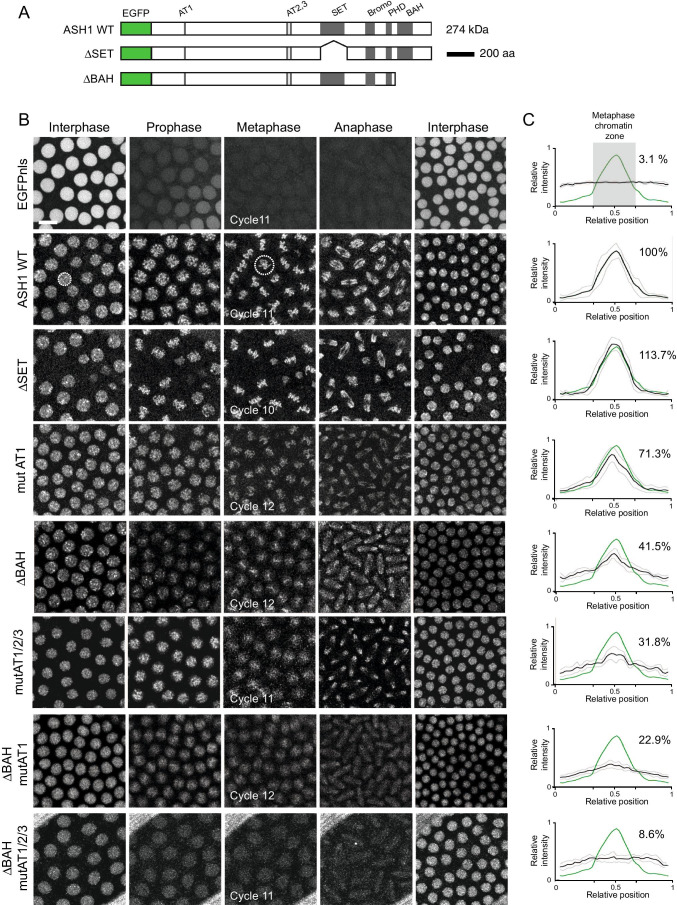
The AT-hooks and the BAH domain mediate chromatin association of ASH1 during mitosis. **A** EGFP::ASH1 fusion protein and variants. Grey: domains according to UNIPROT; green: EGFP tag (green). **B** Confocal images of pre-blastoderm embryos with EGP fusion proteins as shown, at the cell cycle stages indicated. Scale bar represents 10 µm and is the same for all images. Dotted circles indicate the area of interphase and metaphase images used to evaluate total signal intensity. Mitotic cycle number is indicated on metaphase images. **C** Averaged profiles through nuclei centred on the mitotic chromatin zone within maximum-intensity projections at metaphase. For 3 embryos, 7–10 nuclei each were measured. Profiles show mean (black line) and standard deviation (thin grey line) of all nuclei. The y-axis shows the relative average intensity along the profile, calculated as described in “Material and methods”. The ASH1 WT profile is shown in green as reference. Data for EGFPnls and ASH1 are reproduced from Steffen et al. (2013). Mitotic binding for each variant was calculated as % of binding by WT ASH1 in the metaphase chromatin zone as described in methods

We have previously shown that ASH1 remains bound to chromatin throughout mitosis (Steffen et al. 2013). To identify the domains of ASH1 that are required for this interaction, we generated transgenic fly lines expressing variants of ASH1 fused to EGFP (Figs. 1, S1, and S2). All constructs were placed under control of the αTubulin promoter and were integrated at the same genomic location as described previously ((Steffen et al. 2013) and Material & Methods). A preliminary analysis of 21 ASH1::EGFP variants, including deletions of large sections of the protein, and of individual domains (data not shown) identified the SET domain, the BAH domain and the three AT hooks as being of interest for this study. The ASH1 SET domain is a histone methyltransferase domain, dimethylating lysine 36 on histone H3 (An et al. 2011; Dorighi and Tamkun 2013; Gregory et al. 2007; Tanaka et al. 2007). BAH domains of different proteins interact with nucleosomes by diverse mechanisms (Kuo et al. 2012; Noguchi et al. 2006; Onishi et al. 2007). AT-hooks bind to the minor groove of AT-rich DNA (Huth et al. 1997) and are found in many chromatin-associated proteins (Aravind and Landsman 1998). AT hooks are best characterized in the high-mobility group protein HMGA1 (Reeves and Nissen 1990) and the methyl CpG binding protein MeCp2 (Lyst et al. 2016).

EGFP::ASH1 variants were generated in which the SET domain or the BAH domain was lacking, and in which single or multiple AT hooks were mutated (Fig. 1A). To disrupt the AT hooks, the conserved “R-G-R” motif in the centre of the AT-hook was altered to “A-G-A”. These arginine residues are required for chromatin binding of HMGA1 in interphase and mitosis (Fonfría-Subirós et al. 2012; Harrer et al. 2004; Huth et al. 1997).

To determine relative expression levels of the ASH1 variants, we performed several analyses at both RNA and protein levels. Transcript levels were determined by qPCR in embryos and by RNAseq for selected variants in 3rd instar larval wing discs, showing that the wild-type *ash1::GFP* transgenic transcript and the *ash1 variant::GFP* transcripts are consistently expressed at approximately 3–fivefold higher levels than the endogenous *ash1* transcript (Steffen et al. 2013) (Figure S2G and 4B). The qPCR analysis also showed that for selected variants, the difference in transcript levels between GFP:: ASH1 WT and the other variants is less than 1.4-fold. Western blot analysis confirmed that the GFP fusion proteins are not substantially degraded (Figure S2H). We note that due to the large size of ASH1 (> 270 kDa), it was not possible to obtain quantitative transfer at the top (containing ASH1) compared to the bottom of the blot (containing Tubulin loading control). Thus, we cannot reliably estimate relative levels of transgenic protein in the different lines from western blotting. However, the fact that the fusion proteins are intact means that GFP can be used as a proxy for quantifying total amounts of protein.

We have previously shown that the ASH1::GFP transgenic protein is present at approximately 3 to fourfold higher than the endogenous protein in pre-blastoderm embryos and larval brains (Steffen et al. 2013). To determine the nuclear levels of the ASH1::GFP variants presented here, we used two independent methods: live imaging of whole nuclei and FCS quantification, described in detail in “Materials and methods”. Image quantification showed that the total signal detected per nucleus in each interphase was less than 1.2-fold different between the ASH1 transgenic lines (Figure S2E). Interestingly, the amount of GFP detected in interphase was similar to the total amount detected in the subsequent metaphase (over 90% of interphase signal present in metaphase in all ASH1 lines), indicating that the ASH1::GFP fusion proteins are retained in the nucleoplasmic space, despite the syncytial nature of the embryo at this stage (Figure S2E). This is true for all the ASH1 fusions but not for EGFP, whose total signal was approximately twofold higher in interphase than that of the ASH1 lines and was reduced approximately twofold in metaphase.

In addition, we quantified molecule numbers by FCS for each variant in interphase nuclei of pre-blastoderm embryos (Table S1, Figure S2F). For all except two variants, the molecule numbers in the FCS volume were within 1.4-fold of those detected for ASH1 WT. For ASH1ΔBAH mutAT1/2/3 and ASH1ΔBAH mutAT2, the molecule numbers measured by FCS were approximately 2.5-fold and twofold higher respectively than those measured for ASH1 WT (Figure S2F). Thus, the FCS measurements partially disagree with those of the imaging analysis. We note that the estimated FCS volume is 0.104µm^3^, approximately 2000-fold smaller than the total nuclear volume at mitotic cycle 12, and that although they look qualitatively similar, the fusion proteins are not homogeneously distributed in interphase (Figs. 1, S1, S2). We propose that this may have contributed to different concentrations of the fusion proteins in the FCS volume. We do not see evidence of 2–2.5-fold higher total expression of these two variants in images taken under identical microscopy conditions (see Figs. 1, S1 and S2). For this reason, we have used the microscopy-based quantification in the following analysis of mitotic binding.

Mitotic chromatin binding was evaluated by time-lapse microscopy in pre-blastoderm embryos, in which nuclei divide synchronously 13 times in a time window of approximately 2 h (Foe and Alberts^1983^). The interphase images of all variants tested showed no discernable differences. All displayed similar heterogeneous distributions in nuclei of pre-blastoderm embryos (Figure S2A, B). Mitotic chromatin binding of each variant was compared to that of the EGFP::ASH1 wild-type fusion protein by quantitative analysis of metaphase images (Figs. 1, S1, S2D).

Variants in which the SET domain was deleted, or in which the 2nd AT hook was mutated, showed over 90% of ASH1 WT mitotic chromatin binding levels (Figs. 1, S1, S2). Mutation of the first or third AT hook resulted in 71.3% and 77.3% of mitotic binding levels respectively (Figs. 1, S1, S2). Partial loss of mitotic binding was observed upon deletion of the BAH domain alone (41.5% of ASH1 WT levels; Fig. 1), of any two AT hooks (38.8–52.6% of ASH1 WT levels Figure S1) or all three AT hooks (31.8%; Fig. 1). Variants in which the BAH domain was deleted in addition to mutation of any one of the three AT hooks showed a further reduction in mitotic chromatin binding in comparison to deletion of the BAH domain alone (22.9–35.6%; Figs. 1, S1), suggesting cooperativity between the BAH domain and each of the AT hooks. In variants lacking the BAH domain and all three AT hooks, very little enrichment on mitotic chromatin was detectable (8.6%; Fig. 1).

Taken together, these data demonstrate that ASH1 requires at least two of its AT hooks and the BAH domain for full chromatin binding in metaphase, and that the BAH domain and the three AT hooks mediate mitotic chromatin binding in a cooperative manner.

### The AT hooks, the BAH domain and the SET domain are not essential for ASH1 chromatin binding in interphase

**Fig. 2:**
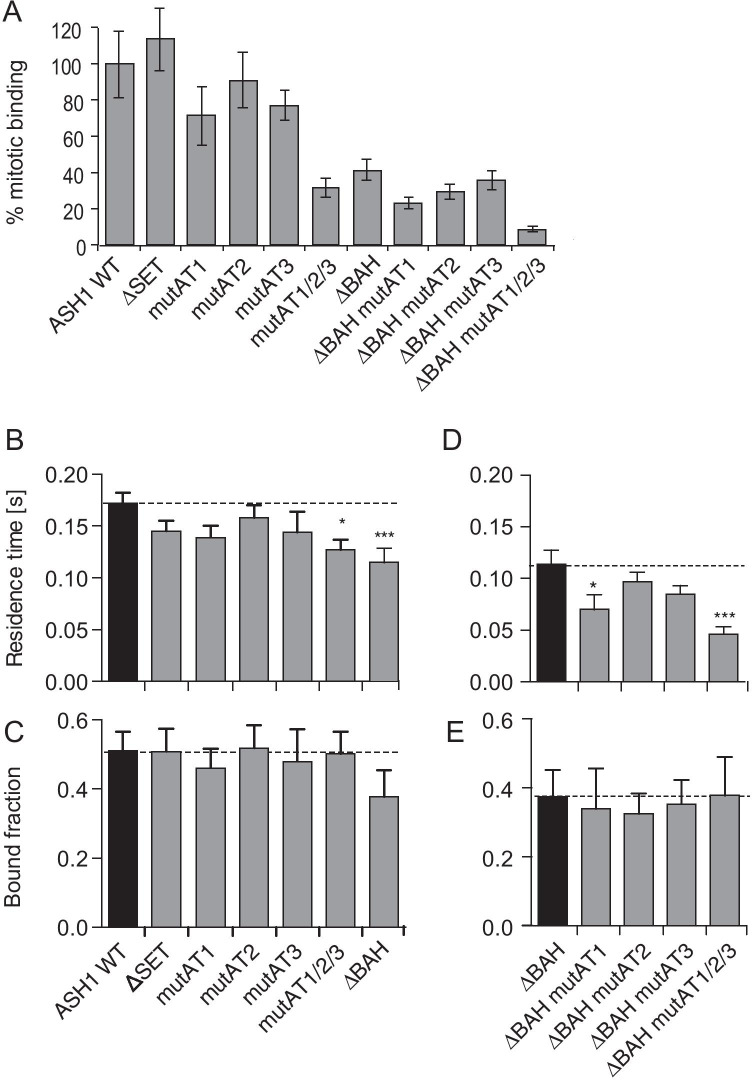
The AT-hooks and the BAH domain are not essential for chromatin association of ASH1 during interphase. **A** Summary of % mitotic binding calculated for GFP fusions as % of GFP::ASH1 WT, extracted from Figs. 1 and S1. **B**–**E** Interphase chromatin binding kinetics was measured by FCS in preblastoderm embryos during cleavage cycles 10–13. See also Table S1. **B**, **D** Residence times; **C**, **E** nound fractions. Error bars represent cumulative standard error of measurements in at least 10 nuclei. Statistical significance was tested using ANOVA with Dunnett’s post-test (α = 0.05) comparing each ASH1 variant against ASH1 WT (**B**, **C**) or ΔBAH (**D**, **E**). *p*-values for ANOVA: *p < 0.01; ***p < 0.001. See also Table S1

The interphase distributions of ASH1 variants appeared similar to one another (Figure S2A, B). However, image analysis alone does not allow reliable conclusions to be drawn regarding chromatin binding, because the bound and unbound fractions are superimposed. To quantify interphase binding by independent means, we used fluorescence correlation spectroscopy (FCS) (Mazza et al. 2012a, b). To determine whether the domains that mediate mitotic chromatin binding are also required for chromatin binding during interphase, we used FCS to measure chromatin binding kinetics of the ASH1 variants in pre-blastoderm embryos (Fig. 2). Kinetic parameters were extracted by fitting reaction—diffusion models to FCS data as described in Steffen et al. (2013) and are shown in Table S1. Residence time and bound fraction were calculated as described in “Materials and methods”.

Deletion of the SET domain affected neither the residence time nor the bound fraction of ASH1, demonstrating that the SET domain is not only dispensable for mitotic chromatin binding (Figs. 1, 2A) but also does not contribute to global chromatin binding of ASH1 in interphase (Fig. 2B). We next examined the role of the AT hooks. Mutation of single AT hooks had no effect on residence time, whilst mutation of all three AT hooks or the BAH domain led to a significant decrease in residence time compared to wild-type ASH1 (25–30%; Fig. 2B). Thus the three AT hooks and BAH domain may contribute to binding during interphase as well as in mitosis. However, none of these variants showed a significant change in the bound fraction of protein (Fig. 2C; Table S1). This is in contrast to the substantial decrease in mitotic chromatin binding observed upon mutation of all three AT hooks or the BAH domain (Figs. 1, Fig. S2C).

Mutation of the first AT hook in the ΔBAH context reduced the residence time to approximately 60% of that of ΔBAH, while mutation of the 2nd or 3rd AT hook had little effect (Fig. 2D). Notably, ΔBAH mut AT2, which showed twofold higher average molecule numbers in the FCS volume than the ΔBAH transgenic protein, did not show a substantially different residence time or bound fraction than ΔBAH. Indeed, these two parameters are independent of protein concentration, being calculated from the off rate (*k*_off_, units, s^−1^) and the pseudo-first-order association rate (k*_on_, units, s^−1^, Steffen et al. 2012). Mutation of all three AT hooks led to a further reduction in residence time to approximately 50% of that of ΔBAH (Fig. 2D). Thus, the AT hooks contribute to interphase binding in combination with the BAH domain. Nevertheless, all of the AT hook mutations in the ΔBAH context showed essentially identical bound fractions to the ΔBAH variant itself (approximately 75% of wild-type levels, Fig. 2E). Furthermore, this reduction in bound fraction was not statistically significant (Fig. 2C, E). Thus, ASH1 can still bind substantially to interphase chromatin in the absence of the BAH domain and the AT hooks. This is in contrast to the severe loss of detectable mitotic chromatin binding for ΔBAH in combination with any single AT hook mutant, and the almost complete loss of detectable mitotic chromatin binding when all three AT hooks are mutated in ΔBAH (Figs. 1, S1, S2). Taken together, these results demonstrate that in contrast to their role during mitosis, the AT hooks and the BAH domain are not essential for interphase chromatin binding.

### ASH1 chromatin binding in metaphase and interphase is independent of FSH-S

ASH1 has been reported to genetically and physically interact with the TrxG protein FSH-S and to colocalise with FSH-S on chromatin in ChIP experiments (Kockmann et al. 2013; Shearn 1989). FSH-S is the *Drosophila* homologue of mammalian BET-family (bromodomain and extra-terminal domain family) proteins, which have been shown to interact with chromatin via their bromodomains (Dey et al. 2003). To address whether chromatin binding of ASH1 depends on its interaction with FSH-S, we examined the interaction using live imaging. We first generated flies carrying an EGFP::FSH-S transgene and investigated its binding behaviour during mitosis and interphase as described above for ASH1 (Figure S3A-E). We observed that FSH-S is strongly enriched on mitotic chromosomes (Figure S3D,E). Both this mitotic interaction and the interphase protein distribution and residence time were substantially reduced upon mutation of the first but not the second bromodomain (Figure S3C-E). Thus we conclude that FSH-S attaches to chromatin in interphase and mitosis via its first bromodomain, likely via interactions with acetylated lysines.

To evaluate whether ASH1 depends on FSH-S for chromatin binding, we used a small molecule inhibitor to interfere with FSH-S chromatin binding. The inhibitor ( +)-JQ1 specifically inhibits the interaction of BET family bromodomains with acetyl lysines (Dawson et al. 2011; Filippakopoulos et al. 2010; Nicodeme et al. 2010). Injection of ( +)-JQ1 into embryos expressing EGFP::FSH-S resulted in dissociation of FSH-S from chromatin during mitosis specifically upon injection of the inhibitor (Figure S3F). This result provides a means to address the dependency of ASH1 upon FSH-S both in interphase and mitosis. Surprisingly, neither mitotic binding (Figure S2G) nor interphase binding (Figure S3H) of ASH1 was detectably affected in ( +)-JQ1-treated embryos. Taken together, these results demonstrate that global chromatin binding of ASH1 during metaphase and interphase occurs independently of FSH-S.

### The ASH1 SET domain, AT hooks and the BAH domain are required for correct cell identity

**Fig. 3:**
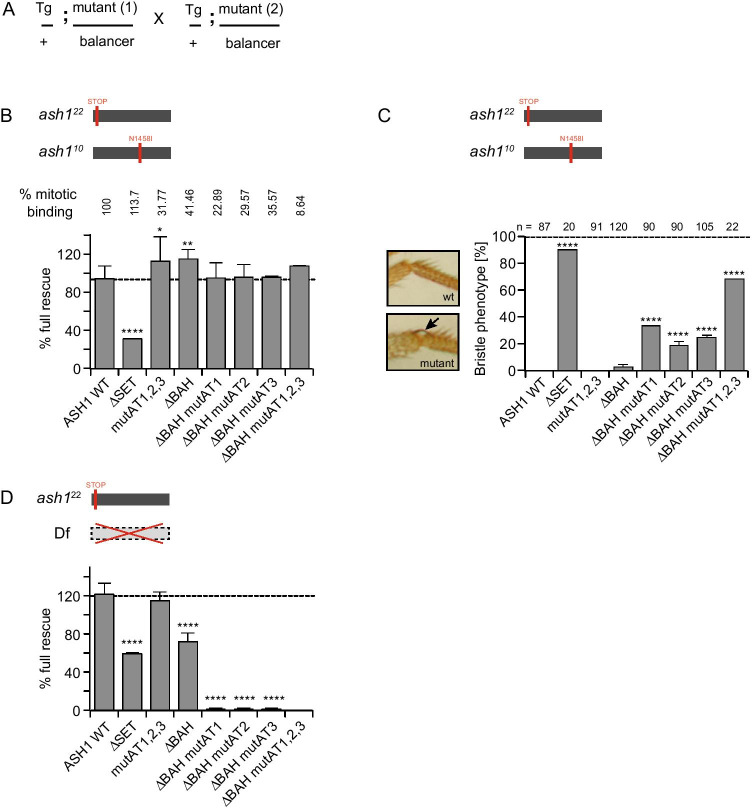
The ASH1 AT hooks and the BAH domain are required for correct cell identity and for survival. **A** Crossing scheme used to evaluate genetic rescues. See “Materials and methods” for details. **B**, **D** Rescue of lethality in *ash1*^22^/*ash1*^10^ (**B**) or *ash1*^22^/DF3L^Exel9011^ (**D**) by ASH1 WT fusion protein a. Molecular lesion in mutant alleles is shown. **B** % mitotic binding according to Figs. 1 and S1 is shown above the plot. **C** Left: 3^rd^ legs of adult flies show ectopic bristle in *ash1*^10^/*ash1*^22^ mutants when rescued by specific variants. Right: frequency of bristle phenotype in *ash1*^10^/*ash1*^22^ flies carrying EGFP::ASH1 variant transgenes as indicated. Number of flies analysed is shown above each bar. All plots show mean and standard deviation of at least two independent crosses. Statistical significance was tested using Fisher’s exact test comparing each variant with ASH1 WT (mutants in full length ASH1 context) or ASH1ΔBAH (mutants in ΔBAH context). *p < 0.01; **p < 0.005; ****p < 0.0005

The experiments described above show that different ASH1 variants are specifically impaired in different aspects of chromatin binding. In order to evaluate whether these variants also show functional differences, we performed a genetic rescue experiment (Fig. 3). The ability of each of the ASH1 variants to rescue lethal combinations of *ash1* mutant alleles was evaluated. We first addressed rescue of lethality of *ash1*^*22*^*/ash1*^*10*^ (Fig. 3A, B). The *ash1*^*22*^ allele carries a premature stop codon after the first 46 amino acids; thus, no functional ASH1 protein is produced from this allele (Tripoulas et al. 1996). The *ash1*^*10*^ allele carries a point mutation in the SET domain, which abolishes histone methyltransferase activity (Byrd and Shearn 2003). Thus, a full-length, catalytically inactive ASH1 protein is produced from this allele. The *ash1*^*22*^ and *ash1*^*10*^ alleles were introduced by crossing balanced heterozygote stocks; thus, a maternal contribution of wild type *ash1* is present during embryogenesis (see “Materials and methods”). In the absence of a rescuing transgene, no mutant adults eclosed. Recent analysis of *ash1*^*22*^ homozygous mutants lacking both maternal and zygotic contributions showed similar results, with lethality at late pupal stages (Schmäling et al. 2018).

The rescue results are shown in Fig. 3B. Surprisingly, the ΔSET variant, lacking the SET domain, gave partial rescue (30% of expected number of mutant adults eclosed). This is consistent with a recent report showing that a transgene carrying a catalytically inactive form of ash1 (*ash1*^*R1464A*^) is also able to partially rescue adult lethality in *ash1*^*22*^ homozygotes, both with and without a maternal contribution of wt *ash1* (Schmäling et al. 2018). This demonstrates that a functional ASH1 SET domain is not strictly essential for survival. All other variants tested gave a full rescue of lethality in this background, demonstrating that they can fully complement the impaired SET domain function of the protein encoded by the *ash1*^*10*^ mutant allele (Fig. 3B).

We next asked whether the flies rescued by different ASH1 variants showed phenotypic differences (Fig. 3C). Loss of function mutations in *ash1* or mutants lacking SET domain activity show a wide variety of homeotic transformations (Schmäling et al. 2018; Dorafshan et al. 2019a). These include the transformation of the 3rd to 2nd legs, resulting in ectopic apical bristles on the distal tibia (Shearn 1989). The ectopic bristle results from a change in cell identity in the sensory organ precursor lineage in the 3rd leg (Rozowski and Akam 2002), (Fig. 3C, left). In flies rescued by wild-type ASH1, none of the survivors showed an ectopic bristle (Fig. 3C). In contrast, over 90% of the flies rescued by ASH1 ΔSET developed an ectopic bristle on the 3rd leg. We next examined the occurrence of the ectopic bristle in flies rescued by other ASH1 variants (Fig. 3C). Strikingly, neither the mutation of all three AT hooks nor the deletion of the BAH domain led to a significant occurrence of ectopic bristles. In contrast, flies in which the BAH domain was lacking in combination with mutation in any single AT hook showed ectopic bristles in 22 to 38% of flies, whilst mutation of all three AT hooks in the ΔBAH context gave rise to the bristle phenotype in 70% of flies. Thus, a change of only two amino acids in an AT hook in the ΔBAH protein causes a sharp increase in the occurrence of homeotic phenotypes. We conclude that in addition to the SET domain, the ASH1 AT hooks together with the BAH domain are required for correct cell identity in the third leg sensory organ precursor lineage.

### A combination of ASH1 BAH domain and AT hooks, but not the SET domain, is required for survival

To evaluate the function of ASH1 variants in a more severe mutant background, we addressed their ability to rescue null mutants in which endogenous ASH1 is completely lacking. To this end, we combined the *ash1*^*22*^ allele (carrying a premature stop codon) with a deficiency in which the *ash1* gene is deleted (Df(3L)Exel9011 Fig. 3D, top). The mutant alleles were introduced by crossing balanced heterozygote stocks, so a maternal contribution of wt *ash1* is present during embryogensis (see “Materials and methods”). Rescue was scored in terms of the number of eclosing adults. In the absence of a rescuing transgene, no mutant adults eclosed, consistent with Schmäling et al. (2018); Dorafshan et al. (2019a). Both the ASH1 wild type and the variant in which all three AT hooks were mutated gave full rescue of lethality in this context, showing that these proteins can fully complement a complete lack of ASH1 (Fig. 3D, bottom). The ΔSET variant gave partial rescue (50%: Fig. 3D) consistent with its ability to partially rescue *ash1*^*22*^*/ash1*^*10*^ (Fig. 3C) and of *ash1*^*R1464A*^ to partially rescue *ash1*^*22*^*/ash1*^*22*^ (Schmäling et al. 2018). Interestingly, the ΔBAH variant also gave partial rescue (65%: Fig. 3D), in contrast to its ability to fully rescue *ash1*^*22*^*/ash1*^*10*^ (Fig. 3C). These results are consistent with a recent report testing the ability of several transgenic ASH1 variants to rescue lethality in the same genetic background, showing that both full-length ASH1 and a variant in which all three AT hooks were deleted gave full rescue as we have also shown here (Dorafshan et al. 2019a). In the same study, ΔSET and ΔBAH transgenes gave partial rescue, as we also observe here. The authors conclude that the AT hooks are not required for ASH1 function. However, these authors did not examine variants in which both the BAH domain and the AT hooks were impaired.

Strikingly, we found that variants with additional mutations in any one or all of the three AT hooks in the ΔBAH context no longer gave rescue of lethality (Fig. 3D). Thus, a change of only two amino acids in an AT hook in the ΔBAH protein abolishes its ability to complement the complete lack of endogenous ASH1 in these flies. We conclude that the AT hooks together with the BAH domain are required for survival.

### Intact ASH1 AT hooks together with the BAH domain are required to maintain expression levels of specific genes

**Fig. 4:**
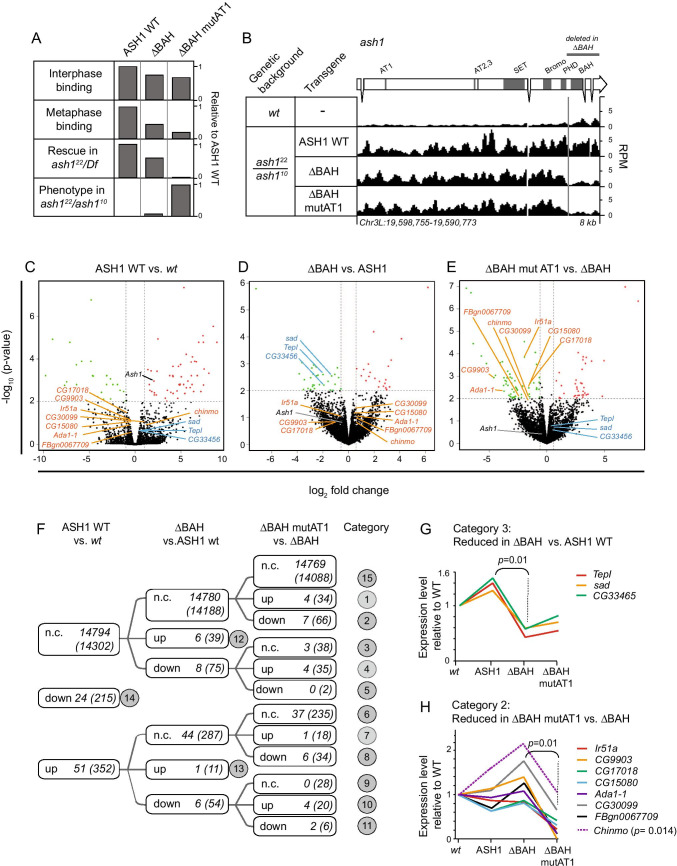
Disruption of ASH1 AT hooks causes misregulation of specific genes. **A** Summary of results of chromatin binding and genetic experiments for EGFP fusions of ASH1 WT and two variants as indicated (data from Figs. 1, S1 and 3; Table S1). **B** RNA-seq tracks showing RPM (reads per million) for *ash1* in 3rd instar larval wing discs of the genotypes shown. **C**–**E** Volcano plots showing comparison of RNA-Seq data from 3rd instar larval wing discs for pairs of genotypes as shown. Genetic backgrounds: “wt” refers to wild-type files; all other genotypes refer to EGFP::ASH1 variants in the *ash1*^10^/*ash1*^22^ background. Capitals refer to EGFP transgenes. X-axis: log_2_ of mean fold change of three replicates for the first genotype *vs* the second. Y-axis: -log_10_ of *p-*value, calculated by *t* test based on the standard deviation of the three replicates as described in the document Supplementary_RNAseq. **F** Summary of RNA-seq data from the 3rd instar larval wing discs showing genes whose mean RPKM changes > twofold between the two genotypes shown at the top of the scheme, after filtering out short genes and lowly expressed genes (see “Materials and methods”). Categories are indicated in grey circles. Gene numbers in each category are given. The first number indicates the number of genes for which the relevant fold change has a *p*-value of < 0.01. The second number in brackets indicates the total number of genes in each category regardless of *p*-value. **G** Relative mean expression values of the three genes in category 3 for which the difference between ΔBAH and ASH1 WT has a *p*-value of < 0.01 are shown. **H** As for **G**, showing the seven genes in category 2 for which the *p*-value for the reduction in ΔBAH mut AT1 compared to ΔBAH is < 0.01. In addition, the *chinmo* gene is shown, category 2, *p* = 0.014 for the reduction in ΔBAH mut AT1 compared to ΔBAH

We have shown that different ASH1 variants are specifically impaired in different aspects of chromatin binding and that these same variants induce different phenotypes when introduced into a mutant background. To address whether these variants affect different sets of downstream target genes, we performed genome-wide mRNA profiling by RNA-seq. We compared wild-type animals with those expressing selected EGFP::ASH1 variants in the *ash1*^*22*^*/ash1*^*10*^ mutant background (Fig. 4). We selected three variants that gave distinct results in the chromatin binding and genetic rescue experiments, namely ASH1 WT, ASH1ΔBAH and ASH1ΔBAHmutAT1 (Fig. 4A). These two mutant variants were selected because they showed only minor differences to ASH1 WT in interphase binding, but showed loss of mitotic binding, viability and cell identity at intermediate (ASH1ΔBAH) and severe (ASH1ΔBAH mutAT1) levels (Fig. 4A). Thus, by comparing these two variants, which differ by only two amino acids, and show strong phenotypic differences, we aimed to determine whether they also affect different genes.

In order to obtain sufficient quantities of a single tissue for this analysis, RNA was extracted from wing discs of mutant 3rd instar larvae. RNA-seq data were confirmed by qPCR on selected genes in the four genotypes (Figure S4) and are summarised in Fig. 4. For full datasets, see Tables S2 and S3. Analysis of the *ash1* locus itself showed that the transgenic *ash1* variants were expressed at equivalent levels to each other in wing discs, giving approximately threefold higher total *ash1* transcript levels in the transgenic animals than in wild type (Fig. 4BC–E). This is consistent with the observed 3–fourfold levels of overexpression of EGFP::ASH1 in wild-type larval brains and embryos, determined previously (Steffen et al. 2013).

Comparison of genome-wide gene expression levels in wild-type wing discs and discs expressing each of the three EGFP fusion proteins in the *ash1*^*22*^*/ash1*^*10*^ mutant background revealed that the majority of genes were unaffected in transgenic discs (Fig. 4C–E). At a *p*-value cut-off of 0.01, 14,769 of the 14,869 genes (over 99%) analysed showed less than twofold change in expression between any pair of genotypes. This is consistent with the full rescue of *ash1*^*22*^*/ash1*^*10*^ mutants by all three transgenes (Fig. 3B). Furthermore, in the comparison of ASH1 WT rescue animals to wild type, 24 genes were downregulated and 51 were upregulated, representing 0.05% of the total. Thus, 99.5% of genes were unchanged compared to wild-type levels, suggesting that the full-length ASH1 WT transgene substantially restores wild-type function. A recent study performed RNA-seq on 3^rd^ instar wing and leg discs of *ash1*^*22*^ homozygous null mutants compared to wild type (Schmäling et al. 2018). This identified approximately 600 genes that were up or downregulated over twofold (*p*-value < 0.01). In our dataset, 32 of these genes showed a significant change in expression level in ASH1 WT transgenic rescue wing discs compared to wild type (see Table S2 for full list).

Figure 4C shows the genes that were down- or upregulated over twofold in ASH1 WT rescue animals compared to wild type for *p* < 0.01 (for full list, see Table S2). For the downregulated genes, we observed a high overlap (21 of 24 genes for *p* < 0.01) with the genes that were downregulated in *ash1*^*22*^ homozygous null mutant wing discs (Schmäling et al. 2018) (see Table S2 for list). Thus, these may represent genes that are downregulated in the mutant background and not rescued by the transgene. In contrast, of the 51 upregulated genes that we identified, 12 of these genes were upregulated in our ASH1 WT rescue, but downregulated (10 genes) or upregulated (2 gene) in *ash1*^*22*^ homozygous null mutants (see Table S2 for lists). The remainder of these 51 genes were not changed in *ash1*^*22*^ homozygous null mutants in Schmäling et al. (2018). Of these 51 upregulated genes, the majority (37 genes, or 72%) showed no further change in expression levels in ΔBAH or ΔBAHmutAT1 rescue animals (Fig. 4F category 6; Table S2). We reason that these genes are upregulated either directly or indirectly by the overexpression of EGFP::ASH1 protein, to an equal extent by the wild type and mutant fusion variants.

We were particularly interested in genes that were not misexpressed in ASH1 WT rescue animals compared to wild type but were only differentially expressed in the presence of ΔBAH or ΔBAHmutAT1. These genes are expected to include targets of ASH1 that specifically depend on the BAH domain or the BAH domain together with the AT hooks for their correct expression. Several genes showed a significant reduction in expression in ΔBAH compared to ASH1WT, but not in any other comparison. These are the genes that responded to the loss of the BAH domain, and thus may be affected by partial loss of mitotic ASH1 binding (category 3, Fig. 4F, marked in blue on Fig. 4C–E). Thirty-eight genes in total fell into this class, of which 3 had a *p*-value of < 0.01 for the comparison of ΔBAH with ASH1 (Fig. 4G). *p*-values were calculated by *t*-test on RNA-seq data from three biological replicates (see document Supplementary RNA-seq and Table S2). Genes that pass the cut-off of *p* < 0.01 are high confidence candidates. To evaluate whether candidates with larger *p*-values may also be of interest, we performed qPCR on genes from different categories as shown in Fig. 4F, and with a range of *p-*values calculated from RNA-seq data (*p* = 1.2 E − 07 to *p* = 0.273; Figure S4). This analysis showed that differences that did not pass the stringent thresholds applied to the RNAseq data were nevertheless clearly present in the qPCR data. Thus, we conclude that the genes identified as significant in RNA-seq are a subset of the truly misregulated genes. Total numbers of genes in each category are given in brackets in Fig. 4F, and the entire set is listed in Tables S2 and S3.

We were also interested in those genes whose expression was reduced in animals expressing ΔBAHmutAT1 compared to ΔBAH, but not in any other comparison. These are the genes that were unaffected by the loss of the BAH domain, but specifically responded to the loss of a single AT hook from the transgenic protein, and thus may be affected by severe loss of mitotic binding (category 2, Fig. 4F, marked in orange on Fig. 4C–E). Sicty-six genes in total fell into this class, of which seven had a *p*-value of < 0.01 for the comparison of ΔBAHmutAT1 with ΔBAH (Fig. 4H). Table 1 shows the genes in category 2 for which a molecular function is known (the less stringent *p*-value cut-off of 0.1 is used). Interestingly, this list contains two genes with roles in transcriptional activation (*Ada1-1* (Guelman et al. 2006) and *chinmo* (Flaherty et al. 2010)) and two for which loss of function or RNAi knockdown leads to lethality (*chinmo* (Flaherty et al. 2010) and *RNaseMRP:RNA* (Schneider et al. 2010)). Interestingly, the *chinmo* gene is a transcription factor involved in several developmental processes including sex determination, control of neuronal identity, tumour formation and stem cell self-renewal (Table 1; Fig. 4E). We note that all of the genes shown in Table 1, except for* Pde1c*, were also downregulated in homozygous null mutants in one or both tissues examined in Schmäling et al. (2018) (Table S2).

**Table 1:** Gene functions

Symbol	Name	Molecular function/ description	Mutant phenotype	*p*-value	Ref
*Ada1-1*	*Transcriptional Adaptor 1–1*	Transcriptional coactivator, contributes to histone H3 acetyltransferase activity of SAGA complex	No information	0.003	Guelman et al. (2006)
*chinmo*	*Chronologically inappropriate morphogenesis*	Zinc finger BTBPOZ transcription factor. Involved in wing morphogenesis, sex determination, neuronal identity, eye development, tumour formation, stem cell self-renewal	Lethal, die before larval stages	0.014	Flaherty et al. (2010)
*eater*	*Eater*	Phagocytic receptor for bacterial pathogens. Required to survive bacterial infection	Viable, immune response defective	0.013	Chung and Kocks (2011) Kocks et al. (2005)
*Ir51a*	*Ionotropic receptor 51a*	Member of family of receptors for internal and external chemical cues. May be a pseudogene	No information	0.00014	Benton et al. (2009)
*Nplp4*	*Neuropeptide-like precursor 4*	Neuropeptide hormone activity	Viable	0.08	Baggerman et al. (2002)
*Pde1c*	*Phosphodiesterase 1c*	Calcium- and calmodulin-regulated 3',5'-cyclic-nucleotide phosphodiesterase activity. Regulates intracellular levels of cAMP and cGMP	Viable, male sterility and male mating defects, reduced copulation rates	0.044	Day et al. (2005) Morton et al. (2010)
*RNase* *MRP:RNA*	*Ribonuclease MRP RNA*	5.8 s rRNA processing	2nd instar larval lethal; growth defect	0.097	Schneider et al. (2010)
*Tsp42Ej; sun*	*Tetraspanin 42Ej; sunglassless*	Transmembrane protein. Cellular response to high light intensity. Endocytosis in response to light	Viable	0.044	Xu et al. (2004) Han et al. (2007)

The table shows genes of category 2 (i.e., they were downregulated only in ΔBAH mutAT1 compared to ΔBAH, and were not deregulated in any other comparison). Genes are shown that have a known function, and for which the *p*-value for the downregulation in ΔBAH mutAT1 compared to ΔBAH is less than 0.1. *p*-values were calculated from RNA-Seq data by *t*-test on the basis of standard deviation of the three replicates, as described in the document Supplementary RNA_Seq

Taken together, these results demonstrate that disruption of the BAH domain, or of a single AT hook in the ASH1 ΔBAH protein leads either directly or indirectly to downregulation of a specific group of genes in each case. Thus, intact ASH1 AT hooks and the BAH domain are required to maintain the expression levels of these genes.

## Discussion

**Fig. 5:**
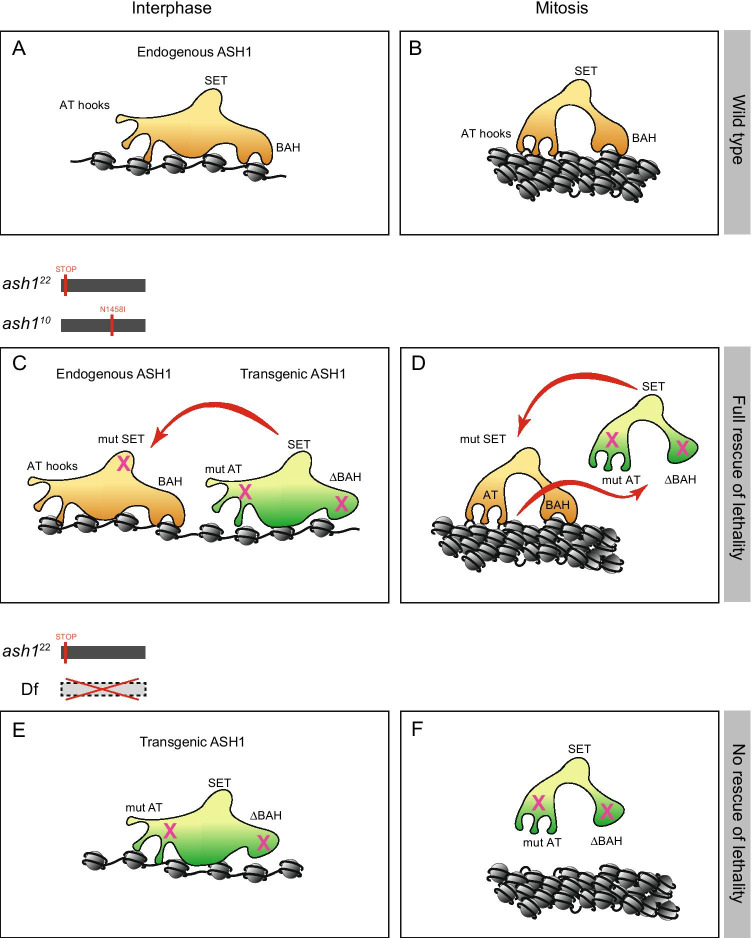
Summary of ASH1 binding modes in interphase and mitosis in wild type and in the genetic rescue. **A**, **B** Wild type. **C**, **D** Rescue of *ash1*^*22*^* /ash*^*10*^ by ΔBAH mutAT variants. Red arrows indicate potential functional compensation between the endogenous and transgenic proteins. **E**, **F** The same transgenic protein fails to rescue lethality of *ash1*^*22*^*/Df*, in which no endogenous ASH1 protein is present. See main text for details

By studying variants of the ASH1 protein fused to EGFP in living *Drosophila*, we have identified domains that are required for mitotic chromatin binding and have investigated the effects of deleting or mutating those domains on cell identity, survival and gene regulation. We show that the ASH1 BAH domain in combination with the AT hooks is required for full mitotic chromatin binding and for survival. In contrast, animals in which ASH1 lacks the SET domain show no impairment of mitotic chromatin binding and are able to survive to adulthood. Thus, we have identified essential domains and functions of ASH1 that are independent of its histone methyltransferase activity. In Fig. 5, we summarise these findings and propose based on the genetic analysis, that mitotic chromatin binding and SET domain functions may complement each other differently during interphase and mitosis. In interphase, the AT hooks and BAH domain stabilise, but are not essential for binding (Fig. 5A). In mitosis, these domains become more important for binding (Fig. 5B). The SET domain is not required for binding in interphase or mitosis. The *ash1*^*10*^ allele encodes endogenous full-length ASH1 protein that carries a loss of function mutation in the SET domain, but no impairment of the AT hooks and BAH domain. The *ash1*^*22*^ /*ash*^*10*^ mutant lethality is fully rescued by ΔBAH mutAT variants and only partially by ΔSET (Figs. 3 and 5C, D).The same transgenic proteins (ΔBAH mutAT variants) fail to rescue lethality of *ash1*^*22*^*/Df*, in which no endogenous ASH1 protein is present (Fig. 5E, F). We propose that in the *ash1*^*22*^* /ash*^*10*^ background, the transgene complements the lack of SET domain function in interphase, while the endogenous protein compensates the mitotic binding defect of the transgenic protein (Fig. 5C, D). The mechanism of this complementation is not known but may involve interactions via other proteins such as MRG15, Caf1 and/or Nurf55 (Huang et al. 2017; Schmäling et al. 2018).

### Mechanisms of mitotic and interphase chromatin binding

We have investigated the AT hooks, the BAH domain and the SET domain and determined their contributions to chromatin binding of ASH1 in vivo. We note that our conclusions relate to global binding characteristics and cannot exclude that any of the domains tested might contribute to chromatin binding differently at specific genes or in specific tissues. To address specific binding sites in vivo, it would be necessary to perform ChIP on each of the ASH1 variants using separate interphase and mitotic chromatin preparations from specific tissues in whole animals. Whilst isolation of mitotic chromatin in sufficient quantities for ChIP is possible in cultured *Drosophila* and mammalian cells and tissues (Blobel et al. 2009; Follmer et al. 2012; Kadauke et al. 2012), the isolation of pure populations of mitotic cells from living *Drosophila* has not so far been reported; thus, such an experiment is currently technically not feasible. We also note that (Schmäling et al. 2018) reported difficulty with ASH1 ChIP-seq in larval tissues, and instead inferred ASH1 function at selected genes from H3K36me2 ChIP qPCR.

#### SET domain

Consistent with earlier studies, we found that the SET domain is required for correct cell identity and for full viability, but that the ΔSET variant can nevertheless partially rescue a null mutant (Schmäling et al. 2018; Tripoulas et al. 1996, Dorafshan et al. 2019a). Surprisingly, deletion of the SET domain did not detectably affect chromatin binding in interphase or metaphase. In addition to its histone methyltransferase activity (An et al. 2011; Dorafshan et al. 2019a, b; Gregory et al. 2007; Schmäling et al. 2018; Tanaka et al. 2007), the ASH1 SET domain has also been shown to interact with RNA and with single-stranded DNA (Krajewski et al. 2005). However, the fact that the SET-domain did not contribute to global chromatin interactions in interphase or mitosis suggests that its in vitro affinity for RNA and DNA is not decisive for global chromatin interactions in vivo. Interestingly, the ASH1 ΔSET variant gave partial rescue of both the hypomorph (which contains a single copy of *ash1* lacking a functional SET domain) and the complete ASH1 null mutant (Fig. 3). This demonstrates that the ASH1 SET domain is not strictly required for survival, consistent with previous observations (Schmäling et al. 2018, Dorafshan et al. 2019a). Interestingly, a recent study reported that complete zygotic substitution of H3 lysine 36 with arginine did not lead to severe loss of Hox gene activity (Dorafshan et al. 2019b). These observations, combined with the fact that *ash1* null mutants are lethal, point to essential roles of ASH1 that are independent of its histone methyltransferase activity. By identifying a requirement for the BAH domain and AT hooks in mitotic chromatin binding and survival, we propose that these domains may indeed contain those essential functions.

#### BAH domain

We have shown that the BAH domain is required both to stabilise chromatin binding of ASH1 during interphase and to play a more essential role during mitosis. BAH domains have been found in DNA methyltransferases, origin recognition complex proteins and factors involved in transcriptional regulation (Callebaut et al. 1999). Several BAH domains have been shown to stabilise or modify interactions with nucleosomes (Kuo et al. 2012; Noguchi et al. 2006; Onishi et al. 2007; Stoddard et al. 2019; Yarychkivska et al. 2018; Zhao et al. 2016). The mechanism by which the BAH domain attaches ASH1 to mitotic chromatin may be different to that in interphase, due to the differences in the chromatin template or post-translational modification of the BAH domain itself.

#### AT hooks

We have shown that mutation of a single AT hook in the context of the ΔBAH mutant is sufficient to severely reduce mitotic chromatin attachment whilst having little effect on the detectable levels of interphase binding. The mutation of all three AT hooks in the ΔBAH mutant almost completely abolishes detectable mitotic chromatin binding whilst only partially reducing interphase binding. This indicates a more stringent requirement for AT hooks for chromatin binding during mitosis than in interphase and suggests that AT hooks may bind to the two platforms via different mechanisms. AT hooks bind to the minor groove of DNA with nanomolar affinity (Huth et al. 1997), have been shown to compete with binding of the linker histone H1 (Catez et al. 2004; Zhao et al. 2011) and to induce DNA-bending (Fonfría-Subirós et al. 2012). The two AT hooks of MeCP2 bind to AT-rich DNA with different affinities (Lyst et al. 2016). The MLL AT-hooks have been shown to bind cruciform DNA and to recognize DNA structure rather than a specific sequence (Zeleznik-Le et al. 1994) and to colocalize with topoisomerase II on mitotic chromosomal scaffolds (Caslini et al. 2000). Thus, AT hooks are attractive candidates for binding to distorted DNA structures, which are highly enriched in mitotic chromosomes (Juan et al. 1996; Michelotti et al. 1997). Interestingly, sites interspersed with the AT hooks of HMG1 protein from the insect *Chironomus* have been shown to be phosphorylated by mitotic kinases, causing modulations in DNA binding affinity (Schwanbeck et al. 2001; Schwanbeck and Wisniewski 1997). In summary, the AT hooks of ASH1 may change their interaction with chromatin and DNA during mitosis both via changes in the available binding surface and by modifications of the properties of the AT hooks themselves.

#### Other proteins

We have shown that FSH-S binds to mitotic chromatin via its first bromodomain, but that ASH1 binding is independent of FSH-S. In the future, it will be of interest to determine the role of FSH-S in mitosis. Recent reports have identified the MRG15, Nurf55 and Caf1 proteins as subunits of the ASH1 complex (Huang et al. 2017; Schmäling et al. 2018). ASH1 has been shown to recruit MRG 15 (Huang et al. 2017). It will be of interest in the future to determine whether MRG 15 and other complex members also remain associated with ASH1 in mitotic chromatin. The MRG interaction domain identified by Huang et al. (2017) was not disrupted in any of the variants we describe here.

### Does maintenance of cell identity, viability and gene expression depend on ASH1 mitotic chromatin binding?

We have shown that variants of ASH1 that are severely impaired in mitotic chromatin binding cause homeotic transformations, deregulate a specific set of genes and are unable to rescue lethality of an *ash1* null mutant. What causes lethality in these variants, and does loss of mitotic chromatin binding play a role? If it does, then it is possible that ASH1 itself contributes to mitotic chromatin integrity and mitotic progression. Interestingly, the mammalian TrxG protein MLL also binds robustly to mitotic chromatin (Blobel et al. 2009) and MLL-deficient cells show mitotic defects (Liu et al. 2007; Mishra et al. 2009).

Alternatively, it is possible that if loss of mitotic binding of ASH1 contributes to lethality, then this may be a direct result of the loss of activation of specific essential genes that require mitotic binding of ASH1 for their correct reactivation after mitosis. Good candidates are those listed in Table 1, for which lethality upon loss of function has been documented in other studies (*chinmo* (Flaherty et al. 2010) and *RNaseMRP:RNA* (Schneider et al. 2010)). Such a “mitotic bookmarking” mechanism has been described for several transcription factors (Caravaca et al. 2013; Deluz et al. 2016; Iberg-Badeaux et al. 2016; Kadauke and Blobel 2013; Kadauke et al. 2012) and for the mammalian TrxG proteins MLL (Blobel et al. 2009) and BRD4 (Dey et al. 2009; Zhao et al. 2011), but has not previously been described for ASH1. We identify candidate genes that may be subject to such regulation and that will form the basis of future analyses. We have identified 66 genes (7 with high confidence) whose expression levels were reduced only in flies carrying ΔBAHmutAT1 compared to ΔBAH but were not significantly affected in any other comparison (category 2: Fig. 4; Table 1). This demonstrates that these genes are unaffected by the deletion of the BAH domain from the transgenic ASH1 but are sensitive to the further loss of a single AT hook. In future, it will be essential to determine at what stage in development and when during the cell cycle this occurs, and whether it involves direct chromatin binding by ASH1.

To determine whether our list of ASH1 targets overlaps with PcG target genes, we compared our deregulated genes with the lists of Enderle et al. (2011), Schwartz et al. (2010) and Schuettengruber et al. (2009). Interestingly we found very little overlap; the vast majority of PcG targets were either in category 16 (too lowly expressed in wing) or 15 (no change in any comparison). We did find two overlaps in categories 2 and 3 with the extended list of targets found by Enderle et al. (2011), in S2 cells by ChIP-seq: *chinmo* and *RNaseMRP:RNA*. In general, the ASH1 targets we have identified (many of which overlap with targets identified independently by Schmäling et al. 2018) are a distinct set from PcG targets. However, it is likely that we have not identified all potential targets of ASH1 mitotic regulation.

To elucidate the role of the ASH1 AT hooks and the BAH domain in gene regulation, and to determine the role of mitotic chromatin attachment, it will be informative to construct reporters for candidate target genes in living *Drosophila* (Zhao et al. 2011). Recent elegant assays for mitotic memory in living embryos based on quantitative analysis of stochastically expressed reporter genes have delivered insights into the timing of post mitotic reactivation and the regulatory DNA sequence requirements for memory (Dufourt et al. 2018; Ferraro et al. 2016). It will be of great interest in future to apply such approaches to the misregulated genes we have identified here.

In summary, this work reveals important properties of ASH1 beyond its role as a histone methyltransferase. To fully understand these properties, it will be important to visualise and quantify the dynamic relationship between mitotic ASH1 binding and gene regulation in living, developing animals.

## Materials and methods

Transgenic *Drosophila* strains expressing EGFP fusion proteins.

Generation of the fly strain expressing EGFP::ASH1 is described in Steffen et al. (2013). Briefly, the ASH1 cDNA was cloned downstream of EGFP and the *αTubulin* promoter in a modified version of the attB plasmid pKC27 which allows site-directed integration into the landing site “43.4” on chromosome IIL at position 38E3 using the φ C31 integrase as described in Okulski et al. (2011) and Ringrose (2009). The EGFP::FSH-S transgenic fly line and the bromodomain mutants EGFP::FSH-S PFV1/2 were generated in a similar way. The plasmids used for injection were generated by PCR amplification of the cDNA sequences (kindly provided by Christian Beisel and Tobias Kockmann). Generation of ASH1 variants for the structure–function analysis is described in detail in Supplementary Material. Plasmids and fly lines are available on request.

### Genetic rescue experiments

Flies expressing EGFP::ASH1 or variants of EGFP::ASH1 were crossed to *Ly*/TM3,*Sb*. In the next generation, EGFP::ASH1/ + ; + /TM3,*Sb* flies were crossed in parallel to *ash1*^10^/TM3,*Ser*, *ash1*^22^/TM3,*Ser* and DF(3L)^Exel9011^/TM6,*Tb*,*Sb* to obtain EGFP::ASH1/ + ; *ash1*^m^/TM3,*Sb,* where *ash1*^m^ stands for any of the three *ash1* deficient alleles. EGFP::ASH1 was followed by the *miniwhite* marker of the transgenes. To address genetic rescue of EGFP::ASH1, progeny of 2 different *ash1* alleles was intercrossed, and the combination of alleles was assayed by the absence of the *Sb* marker. Thus, the progeny received a transheterozygous combination of either *ash1*^22^ and *ash1*^10^ or *ash1*^22^ and DF3L^Exel9011^. In Fig. 3, “Full rescue” (i.e. 100%) is defined as 33% of *mw* + adult progeny having the transheterozygous mutant allele combination (i.e. lacking a balancer chromosome: the other 67% carry heterozygous balancer; homozygous balancers are lethal). At least 300 flies for each transgene for each mutant background were analysed.

### Live imaging of Drosophila embryos

Embryos were dechorionated by a 2-min treatment with 50% household bleach (2.8% hypochlorite) and transferred on a no. 1.5 coverslip (60 × 24 mm) that was covered with a thin layer of embryo glue (Ringrose 2009). The embryos were covered with a thin layer of Voltalef 10S oil before mounting the coverslip upside down on a wet chamber (Reed et al. 2009). Images were acquired during cleavage cycles 10–13 of embryogenesis. Live imaging was performed at room temperature on a Zeiss LSM780 microscope. For time-lapse microscopy, a Plan-Apochromat 40 × /NA 1.3 Oil Lens was used. EGFP fluorescence was excited with a 488-nm Argon Laser at 1–4% power. Twelve-bit images of 512 × 512 pixels were acquired with a zoom of 4.3 resulting in images with a pixel size of 100 nm. Z-stacks of 20–30 μm were acquired every 15 s with a slice interval of 1 μm. The confocal pinhole was set to 2.65 Airy units. EGFP fluorescence was detected in a window of 490–560 nm with a master gain of 750 V. Time-lapse microscopy datasets were deconvolved using Huygens Core (SVI) using the Classic Maximum Likelihood Estimation approach with a theoretical point-spread function and 40 iterations. Figures 1, S1 and S2 show maximum-intensity projections of deconvolved z-stacks.

### Quantification of total GFP signals and mitotic chromatin binding

Mitotic chromatin binding of EGFP fusion proteins was observed by confocal time-lapse microscopy. All measurements were performed on maximum-intensity projections of deconvolved z-stacks collected as described above. Seven to ten nuclei in 3 embryos for each line were analysed. The mean and standard deviation of all nuclei for a given line are shown in the figures. We found that there was more variation between the nuclei of a single animal than between averages measured in different animals, due to the irregular shape of mitotic chromosomes. Thus, we show the larger standard deviation of the mean of all nuclei. Nuclei from cleavage cycles 11–13 were analysed. Cleavage cycle in the representative images shown in the figures was determined by nuclear size and is indicated in the figure panels. Nuclear volume decreases during these cycles from 300 µm^3^ before cleavage 10, to 55 µm^3^ after cleavage 13 (Steffen 2012). The relative intensity per area was constant across nuclear divisions, indicating that the total amount of protein per nucleus decreased with decreasing size, but the relative detectable amount remained constant with respect to area of the image. Thus, to correct for size differences, all images were scaled so that interphase nuclei were 37 pixels in diameter, giving a diameter of metaphase nuclear area of 52 pixels (see Fig. 1). This area was determined by examining the images of those proteins that substantially dissociate but remain visibly associated with the position of each metaphase plate in a larger area during metaphase (e.g. D BAH mut AT 1/2/3: Fig. 1). Total pixel intensities in a circular area of 37 (interphase) or 52 pixels (metaphase) diameter were measured for each transgenic line using ImageJ. Since interphase and metaphase images were acquired with identical settings, the proportion of metaphase signal to that of the preceeding interphase gives an indication of the extent of retention of EGFP fusions in the perinuclear volume during mitosis. In order to quantify relative levels of metaphase chromatin binding, intensity profiles were measured in a rectangular area of 46 × 16 pixels with the long axis perpendicular to the mitotic chromatin plate using the plot profile function of ImageJ. In cases with no mitotic chromatin association of the EGFP fusion protein, the intensity profile was measured along the assumed axis of the nuclear division as seen from later time points. Profiles were normalized to the total signal intensities measured in metaphase (i.e. in the circular 52-pixel diameter area) for each line. The normalization results in profiles in which the area under the profile curve is proportional to the sum of total signal measured in metaphase for that line. Baseline levels vary, according to the proportion of total signal that is colocalised with mitotic chromatin or located in the surrounding nucleoplasm. For each line, the proportion of signal measured in the metaphase chromatin zone (pixels 16–30) above this nucleoplasmic level was measured. The % mitotic binding was calculated by comparing these levels for each variant to that of EGFP ASH1 WT, which was set to 100%.

### FCS

Fluorescence correlation spectroscopy measurements were performed using a Zeiss LSM780 confocal laser scanning microscope mounted on an upright Zeiss Axioobserver stand. Embryos were mounted in wet chambers as described above. Selection of nuclei for FCS measurements was performed in LSM mode, and at least 5 FCS measurements of 5 s each were acquired at the selected spot. Further processing was performed using custom-developed MATLAB scripts, which are available on request. Individual correlation curves were screened for outliers, and the remaining curves of a single nucleus were averaged. Different variants (pure-diffusion/full model) of a reaction–diffusion model (Michelman-Ribeiro et al. 2009) were fitted to the measured correlation curve. The diffusion coefficient (*Df*) and the rate constants k^*^_on_ (pseudo-first order association rate) and *k*_*off*_ (dissociation rate) were extracted from reaction–diffusion model fits. Bound fractions (= k^*^_on_ /(k^*^_on_ + *k*_*off*_) and residence times (= 1/ *k*_*off*_) were calculated using the mean values of the extracted rate constants.

The FCS volume was determined from measuring autocorrelation curves of a dilution series of fluorescein. We performed a calibration of the FCS measurement volume using a concentration series (5–100 nM) of fluorescein. FCS autocorrelation curves were fitted with a fixed diffusion coefficient (Df = 425 µm^2^ s^−1^ (Culbertson et al. 2002, Steffen 2012)). Linear regression of number of molecules in the FCS measurement volume (*n*) and the known fluorescein concentrations (c) was performed. The FCS volume (in litres) is *V* = *n*/(NA.*c*) where *n* is the number of molecules determined from the fit, NA the Avogadro constant and *c* the molar concentration of the fluorescein solution. The FCS volume thus determined was 0.104 femtolitres (= 0.104 µm^3^).

### Western blot

Whole protein extracts of overnight collections of transgenic *Drosophila* embryos were prepared under denaturing conditions as described in Wodarz (2008), using 2 × NuPAGE LDS sample buffer (Invitrogen) as the extraction buffer. Proteins were run at 150 V on NuPAGE Tris acetate 3–8% minigels (Invitrogen) in 1 × NuPAGE Tris–acetate SDS running buffer (Invitrogen) and blotted onto PVDF membrane (Thermofischer) in 2 × NuPAGE Transfer buffer (Invitrogen) with 10% methanol and no SDS, in a semi dry blotting apparatus at 30 V for 1 h at room temperature. Membranes were blocked in PBST (PBS, 0.05% Tween 20), 5% BSA for 30 min at room temperature. The membrane was cut at 55 kDa to separate the portions containing ASH1 (> 270 kDa) and tubulin (50 kDa), and the two parts were subsequently incubated at 4 °C overnight separately with primary antibodies diluted in blocking buffer, as follows: αAsh1 rabbit polyclonal serum (provided by Jürg Müller and described in (Schmäling et al. 2018) 1:2,000, αAlpha-Tubulin mouse monoclonal (Merck T5168) 1:10,000. Membranes were incubated with secondary antibodies for 1 h at room temperature, separately as follows: top portion, horseradish peroxidase–conjugated α rabbit IgG (Merck NA934) 1:5000; bottom portion, horseradish peroxidase–conjugated α mouse IgG HRP (Merck NA913V), 1:10,000. Signals were detected with ECL Plus detection reagent (Pierce) and visualized using a LI-COR C-DiGit Chemiluminescence Western Blot Scanner.

### Injection of JQ1 for live imaging

Embryo injections were performed as described in Ringrose (2009) with minor modifications. Briefly, embryos were collected for 30–60 min and dechorionated using 50% household bleach for 2 min. 50 embryos were aligned in a row and transferred on a no. 1.5 coverslip (60 × 24 mm, Menzel) covered with a thin layer of embryo glue (Ringrose 2009). The embryos were dried for 12 min before injection. A 10-mg/ml stock of ( +)-JQ1 dissolved in DMSO was used to prepare injection mixes containing 0.05 mg/ml ( +)-JQ1 and 0.5 mg/ml Hoechst 34580 diluted in a (2-hydroxypropyl)-beta-cyclodextrin solution (45 g/100 ml water). The mock injection mixes lacked ( +)-JQ1. The injected embryos were covered with Voltalef oil (Sigma-Aldrich). The coverslip was mounted on a wet chamber as described by Reed et al. (2009) and directly used for live imaging experiments.

### RNA extraction, cDNA synthesis and quantitative PCR analysis

Three independent RNA samples were prepared from each genotype. Five micrograms of total RNA was extracted from dissected 3rd instar larval wing discs or embryos using Trizol Reagent (Ambion). RNA was additionally treated with DNase (Turbo DNase, Ambion). cDNA was synthesized using the SuperScript II kit (Invitrogen) according to the manufacturer’s instructions. Priming was performed with equal amounts of oligo(dT) and random decamer primers (Ambion). RNA was removed by treatment with RNase H (NEB) before PCR analysis or preparation of sequencing libraries. qPCR analysis shown in Figure S2G was performed using SYBR Green sso master mix (Bio-Rad) in a CFX Real time PCR cycler (Bio-Rad). qPCR analysis shown in Figure S4 was performed using SYBR Green JumpStart Taq ReadyMix (Sigma) in a Realplex MasterCycler (Eppendorf). Primer sequences are given in Supplementary Material.

### RNAseq

mRNA was purified from 5 µg total RNA using Oligo (dT)25 Dynabeads (Invitrogen), fragmented by heat treatment and converted to cDNA using Superscript III (Invitrogen). Sequencing libraries were prepared with the KAPA Library Preparation Kit Illumina series (KK8201). After adapter ligation, library fragments of 250–800 bp were isolated from an agarose gel and quantified using the Bioanalyser (Agilent). The DNA was PCR amplified with Illumina primers for 15 cycles, purified and loaded on an Illumina flow cell for cluster generation. Libraries were sequenced on the Illumina HiSeq 2000 at the Vienna Biocentre sequencing facility. Reads were aligned to the *Drosophila* genome (dm3/BDGP5) using TopHat version 2.0.9 (Langmead et al. 2009). Further information on bioinformatic analysis of RNAseq data is given in the document Supplementary_RNA_seq and the legend to Table S2. Note that this document and Table S3 contain all information on genes that were significantly deregulated. Manual inspection of tracks revealed that several of these genes are very short (e.g. tRNAs and snoRNAs, less than a few hundred base pairs) whilst others are very lowly expressed in all four genotypes. These two classes of genes potentially give false positives with low *p* values, because they typically contain only a few reads per gene. For example, we observed that a very short or lowly expressed gene may have two or three reads per gene in all three replicates of one genotype and one or zero reads in all replicates in the other genotype, leading to a false positive hit for what is in effect, random variation. For this reason, we manually removed all genes < 200 bp and all genes for which the sum of RPKM across all four genotypes was < 1.0. The filtered data are given in Table S2. The gene numbers shown in Fig. 4F refer to these filtered lists. The full unfiltered RNAseq data have been submitted to GEO with the record number GSE95226.

## Supplementary Information

Below is the link to the electronic supplementary material.

Supplementary file1 (PDF 4461 KB)

Supplementary file2 (PDF 1054 KB)

Supplementary file3 (XLSX 4891 KB)

Supplementary file4 (XLSX 3366 KB)

## Data Availability

The RNA-seq data sets generated in this study are available at GEO with the record number GSE95226.
