# Supplementary material for: The Trithorax group protein ASH1 requires a combination of BAH domain and AT hooks, but not the SET domain, for mitotic chromatin binding and survival
Source: Chromosoma. 2021 Jul 31;130(2-3):215–34. doi: 10.1007/s00412-021-00762-z (PMC8426247; doi:10.1007/s00412-021-00762-z)
Supplement: Supplementary file 1 — Supplementary file1 (PDF 4461 KB) [file 412_2021_762_MOESM1_ESM.pdf]

Chromosoma: Supplementary information.

The Trithorax group protein ASH1 requires a combination of BAH domain and AT hooks, but not the SET domain, for mitotic chromatin binding and survival

Philipp A. Steffen[1], Christina Altmutter[1], Eva Dworschak[1], Sini Junttila[2], Attila Gyenesei[2], Xinzhou Zhu[3], Tobias Kockmann[3] and Leonie Ringrose[1,4 §]

1) Institute of Molecular Biotechnology (IMBA), Dr. Bohr-Gasse 3, 1030 Vienna, Austria.

2) Vienna Biocenter Core Facilities GmbH (VBCF), Dr. Bohr-Gasse 3, 1030 Vienna, Austria.

3) Department of Biosystems Science and Engineering, ETH Zürich, Mattenstrasse 26, 4058 Basel, Switzerland.

4) Insistute of Biology, Humboldt-Universität zu Berlin, Philippstrasse 13, Haus 22, 10115 Berlin, Germany

§ Corresponding author:
Email: leonie.ringrose@hu-berlin.de
Tel: +49 30 2093 49772

Supplementary Materials and Methods

Fly stocks

Fly strains used in this study are shown in the table below. Fly stocks were maintained on standard medium at 18°C. Crosses and expanded stocks were maintained at 25°C. Embryo collections were performed on apple juice agar plates at room temperature.

Name	Genotype	Ref.
43.4	y^-, w^{1118}; P{43.4, y^+}	(Okulski et al., 2011)
EGFP::ASH1 (tub. prom.)	y^-,w^{1118}; P{pTub - EGFP::ASH1, w^+}43.4	(Steffen et al., 2013)
EGFP::ASH1 variants (tub. prom.)	y^-,w^{1118}; P{pTub - EGFP::ASH1 variants, w^+}43.4	this study
EGFP::FSH-S (tub. prom.)	y^-,w^{1118}; P{pTub - EGFP::FSH-S, w^+}43.4	this study
EGFP::FSH-S PFV1 (tub. prom.)	y^-,w^{1118}; P{pTub - EGFP::FSH-S PFV1, w^+}43.4	this study
EGFP::FSH-S PFV2 (tub. prom.)	y^-,w^{1118}; P{pTub - EGFP::FSH-S PFV2, w^+}43.4	this study
EGFPnls (tub. prom.)	y^-,w^{1118}; P{pTub - EGFPnls, w^+}43.4	(Steffen et al., 2013)
ash1^{10}	w^-; ; *ash1*^{10}/TM3,*Ser*^{1}	F. Sauer
ash1^{22}	w^-; ; *ash1*^{22}/TM3,*Ser*^{1}	F. Sauer
Df(3L)Exel9011	w^{1118}; Df(3L)Exel9011/TM6B,*Tb*^{1}	Bloomington #7945

Generation of EGFP::FSH-S fusion protein fly lines

The EGFP::FSH-S transgenic fly line and the bromodomain mutants EGFP::FSH-S PFV1/2 were generated in a similar way as described earlier for EGFP::ASH1 (Steffen et al., 2013). The plasmids used for injection were generated by PCR amplification of the cDNA sequences (kindly provided by Christian Beisel) using the primers FSH-S_fw/FSH-S rv and direct cloning into pKC27-α1tub-EGFP::ASH1 using BglII and MluI, thereby replacing the coding sequence of ASH1. Plasmids were used for injection into embryos for site-directed integration into the landing site "43.4" according to (Ringrose, 2009) with the exception that the integrase was not provided by co-injection of the integrase plasmid but rather by a genetic cross to the integrase line ZH11 (Bischof et al., 2013).

Generation of EGFP::ASH1 deletion variants for the in-vivo structure function analysis

The ASH1 variants for the structure-function analysis were designed based on the plasmid pKC27-α1tub-EGFP::ASH1 that was used to generate the EGFP::ASH1 fly line (Steffen et al., 2013).

EGFP::ASH1ΔAT1:
pKC27-α1tub-EGFP::ASH1 was digested by XhoI/SphI and the N-terminus of ASH1 was replaced by a 3.6 kb PCR product amplified from pKC27-α1tub-EGFP::ASH1 using the primers PS189/PS187. This removes the amino acids 1-375 of ASH1.

EGFP::ASH1ΔAT1-3:

The N-terminus of ASH1 was removed by digestion of pKC27-α1tub-EGFP::ASH1 with XhoI/SphI and insertion of a 0.8 kb PCR product amplified from pKC27-α1tub-EGFP::ASH1 using the primers PS188/PS187. This removes the amino acids 1-1268 of ASH1.

EGFP::ASH1ΔSET:
A 3.3 kb Bsu36I/SphI fragment was removed from pKC27-α1tub-EGFP::ASH1 and replaced by a 2.7 kb PCR product amplified from pKC27-α1tub-EGFP::ASH1 using the primers PS211/PS212. This removes the amino acids 1339-1564 of ASH1.

EGFP::ASH1 N-term. + SET:
A 2.2 kb SphI/MluI fragment was removed from pKC27-α1tub-EGFP::ASH1 and replaced by a 250 bp PCR product amplified from pKC27-α1tub-EGFP::ASH1 using the primers PS202/PS203. This removes the amino acids 1629-2224 of ASH1.

EGFP::ASH1ΔBAH:
A 2.2 kb SphI/MluI fragment was removed from pKC27-α1tub-EGFP::ASH1 and replaced by a 1.2 kb PCR product amplified from pKC27-α1tub-EGFP::ASH1 using the primers PS202/PS286. This removes the amino acids 1950-2224 of ASH1.

EGFP::ASH1ΔBromo:
The bromodomain of ASH1 was removed using overlap-extension PCR according to (Heckman and Pease, 2007). A 0.5kb PCR product was generated from pKC27-α1tub-EGFP::ASH1 using the oligonucleotides PS202/287 which contains the sequence between the SphI site and the beginning of the bromodomain. A second 1.6kb PCR product was amplified using the oligonucleotides PS288/289 that contains the sequence from the end of the bromodomain up to the MluI site. Both PCR products overlap because PS287 has an overhang that anneals to PS288. A second PCR reaction using the oligos PS202/289 and a mixture of both previous products as templates was used to generate a 2.1kb DNA fragment with the deletion of the bromodomain that was cloned into pKC27-α1tub-EGFP::ASH1 using the restriction enzymes SphI/MluI. This removes the amino acids 1698-1770 of ASH1.

EGFP::ASH1_N-terminus:
A 5.5 kb Bsu36I/MluI fragment was removed from pKC27-α1tub-EGFP::ASH1 and replaced by 2.7 kb PCR product amplified from pKC27-α1tub-EGFP::ASH1 using the primers PS211/PS215. This removes the amino acids 1339-2224 of ASH1.

EGFP::ASH1 C-terminus:
A 1.8 kb fragment containing the C-terminal domains of ASH1 was amplified from pKC27-α1tub-EGFP::ASH1 using the primers PS216/PS217 which brings in flanking XhoI and MluI sites. The PCR product was then cloned into pKC27-α1tub-EGFP::ASH1 using XhoI and MluI. This construct contains the amino acids 1629-2224 of ASH1.

Generation of EGFP::ASH1 AT-hook variants by site-directed mutagenesis

Point mutations in the AT-hooks were introduced using site-directed mutagenesis (Quikchange II XL, Stratagene). 50 ng template and 125 ng of each oligonucleotide were used for the PCR mutagenesis reaction. The elongation time was set to 14 min for the approximately 14 kb large EGFP::ASH1 plasmids. All other steps were performed according to the instructions of the

manufacturer. The first AT-hook was mutated using the primers PS323/324, the second using the primers PS193/PS194 and the third AT-hook was mutated using PS195/196 each of them changing the conserved “R – G – R” motif into “A – G – A”.

Primers used for cloning

Name	Sequence 5’ – 3’
FSH-S_fw	AGATCTCGAGATGTCGTCCAGTGAGCCACCG
FSH-S rv	ACGCGTCTAACCTGCTTCACTGTCGCTCGAG
PS187	CCGTTGAGCATGCTTTTTGGCCTTCTGCTT
PS188	AGGCACTCGAGCTCCGAAGGACTGAAATGGAC
PS189	AGGCACTCGAGCGAGAGATCGACGTGAACAAG
PS193	CACCAGCTAGAGGAAGACCGGCAGGTGCAAAACCTAAGAACAGGGAAC
PS194	GTTCCCTGTTCTTAGGTTTTGCACCTGCCGGTCTTCCTCTAGCTGGTG
PS195	CTGAGATAAGGCCAGCCAAAAAAGCTGGCGCGCAACCCAAGCAG
PS196	CTGCTTGGGTTGCGCGCCAGCTTTTTTGGCTGGCCTTATCTCAG
PS202	GCCATCTGGAGAGGGACTTT
PS203	AGTGCTACGCGTTTATGCTGGCGAGGATGCAGT
PS211	GTTACAAGTCCGATGCCAGC
PS212	GATCAGCATGCTTTCCCGCCAAATTTGGACG
PS215	GATCACGCGTTTATCCCGCCAAATTTGGACG
PS216	CATGCTCGAGATGCTTGGCACCACTAATGGG
PS217	CAGTACGCGTTTATGTTGAGTTGGCCGTAGA
PS286	AGTGCTACGCGTTTAGCCGGACTCATCCTTGAT
PS287	GGACTGCAGCGCTTTTTTGAGGGTCTCCATGGCAC
PS288	AAAGCGCTGCAGTCCCTGAAGGATAGCTATGAGCA
PS289	TGGATCTGGATCACCTAGGT
PS323	AGCTGTTCCTCGAAAGCGCGCAGGTGCACCCAAAAAAGTGGTTCCC
PS324	GGGAACCACTTTTTTGGGTGCACCTGCGCGCTTTCGAGGAACAGCT

Primers used for qPCR

Name	Sequence 5’ – 3’
TBP fwd	CATCGTGTCCACGGTTAATCT
TBP rev	GAAACCGAGCTTTTGGATGAT
inGFP_fwd	CTTCAAGATCCGCCACAACATCG
inGFP_rev	TCGTCCATGCCGAGAGTGATCC
ASH1_fwd2	GAGTTACCTAACCTGGTGC
ASH1_rev2	GACTAGGGTCTCCTCCTTAGC
CG32572 exon 3 fwd	GGTTTCAAGGAGGTGAAGGAC
CG32572 exon 3 rev	GGATTCTTGGTGTGGTTGCTA
swi2 exon 3 fwd	GGCCAAAGGATGGTCTAAATC
swi2 exon 3 rev	CGCGATGAGATGTACGAGAG
et exon 2 fwd	AATCAACGGACTCGTACAACG
et exon 2 rev	CACACATGACTCTGGATGACG
CG6356 exon 1 fwd	CAGGAACCACAATCAACACAA
CG6356 exon 1 rev	GGCAGAGGTGGGAGATAAGAG
Gbp fwd	TCCAAAGGATGACGACGATAC
Gbp rev	TGTACTTTCCAAACGGAGTGC
Ir51a 1 fwd	ATTTGTGGAGTACCTGCATCG
Ir51a 1 rev	CCTGACCGGAACAATAAGACA
CG7497 exon 1 fwd	AACGGGTTTCCATCTACCAGT
CG7497 exon 1 rev	CGATTTCGATTTGCATAGAGC
Lip1 exon 1 fwd	CTTCCGTGAAAGTGAAACTCG
Lip1 exon 1 rev	TTCTTCTTCCTCCTCCTCGTC

Supplementary Figures and Tables

Fig. S1 Contributions of individual AT-hooks to chromatin association of ASH1 during mitosis

A) EGFP::ASH1 WT fusion protein. Grey: domains according to UNIPROT; green: EGFP tag (green). B) Living blastoderm embryos were imaged using timelapse confocal microscopy to visualize expression and localization fusion proteins as indicated, during cleavage cycles 10-13 of embryogenesis. Individual timepoints corresponding to different cell-cycle phases are indicated. Dotted circles indicate the area of interphase and metaphase images used to evaluate total signal intensity. Mitotic cycle number is indicated on metaphase images. Images are maximum-intensity projections of z-stacks from deconvolved time-lapse experiments. Scale bar represents 10 µm and is the same for all images. C) Averaged profiles through nuclei centered on the mitotic chromatin zone within maximum-intensity projections at metaphase. For 3 embryos, 7-10 nuclei each were measured. Profiles show mean (balck line) and standard deviation (this grey line) of all nuclei. The y-axis shows the relative average intensity along the profile, calculated as described in material and methods. The ASH1 WT profile is shown in green as reference. Mitotic binding for each variant was calculated as % of binding by WT ASH1 in the metaphase chromatin zone as described in methods.

Fig. S1

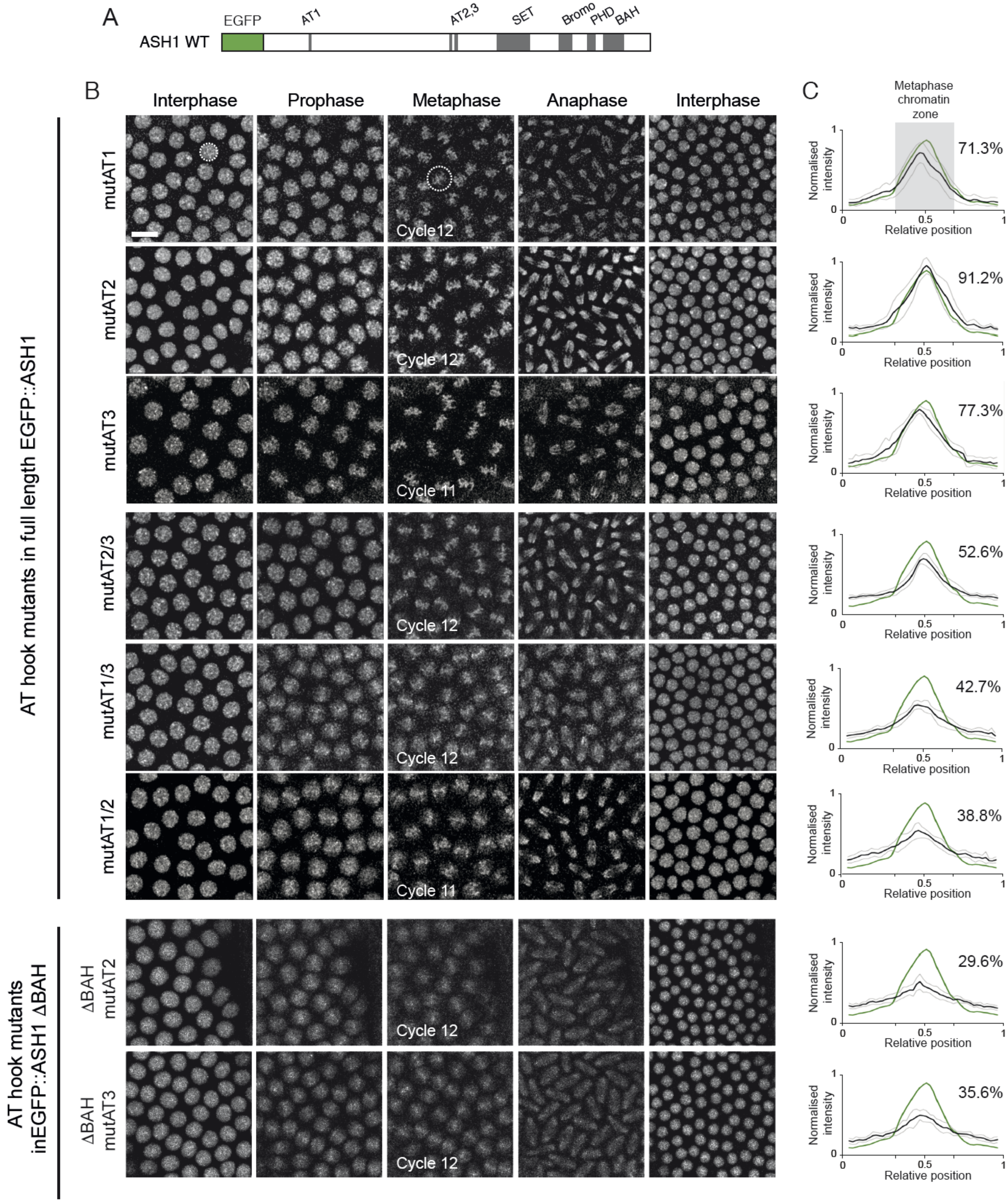
A
ASH1 WT
EGFP
AT1
AT2,3
SET
Bromo
PHD
BAH
B
Interphase
Prophase
Metaphase
Anaphase
Interphase
AT hook mutants in full length EGFP::ASH1
mutAT1
mutAT2
mutAT3
mutAT2/3
mutAT1/3
mutAT1/2
Cycle12
Cycle 12
Cycle 11
Cycle 12
Cycle 12
Cycle 11
AT hook mutants inEGFP::ASH1 ΔBAH
ΔBAH mutAT2
ΔBAH mutAT3
Cycle 12
Cycle 12
C
Metaphase chromatin zone
Normalised intensity
Relative position
71.3%
91.2%
77.3%
52.6%
42.7%
38.8%
29.6%
35.6%

Fig. S2 Quantification of interphase and metaphase binding for ASH1 and variants
A) Magnification of interphase images from Figures 1 and S1 for ASH1 and selected variants as indicated. Scale bar represents 10 μm and is the same for all images. B) Surface plot of the uppermost nucleus in each image of (A), showing that all variants display a similar heterogeneous distribution in interphase. Surface plots were generated using the 'analyze' function of ImageJ64. C) Magnification of metaphase images from Figures 1 and S1. Despite similar interphase distributions, the ASH1 variants show different extents of chromatin binding at metaphase. D) Mitotic binding for each variant was calculated as % of binding by WT ASH1 as described in methods. E) Total mean fluorescence intensity in interphase and metaphase was calculated within the dotted circles shown in (A) and (C) as described in methods. For 3 embryos, 7-10 nuclei each were measured. Mean and standard deviation of all nuclei in each variant are shown. F) Molecule numbers measured by FCS in the FCS volume of 0.104 μm^3 (representing approximately 1/2000th of the entire nuclear volume). G) qPCR analysis on cDNA from 0-16h old embryos expressing the GFP::ASH1 fusion proteins as indicated. Primers specifically detecting GFP or ASH1 enable the relative endogenous and transgenic levels to be determined. TBP, TATA binding protein. Mean and standard deviation of at least two biological replicates for each transgenic line is shown. H) Western blot of whole protein extracts from 0-16h old embryos expressing the GFP::ASH1 fusion proteins as indicated. The top part of the blot was incubated with α ASH1. The bottom part was incubated with α Tubulin. The whole membrane is shown, indicating intact ASH1 fusion proteins.

Fig. S2

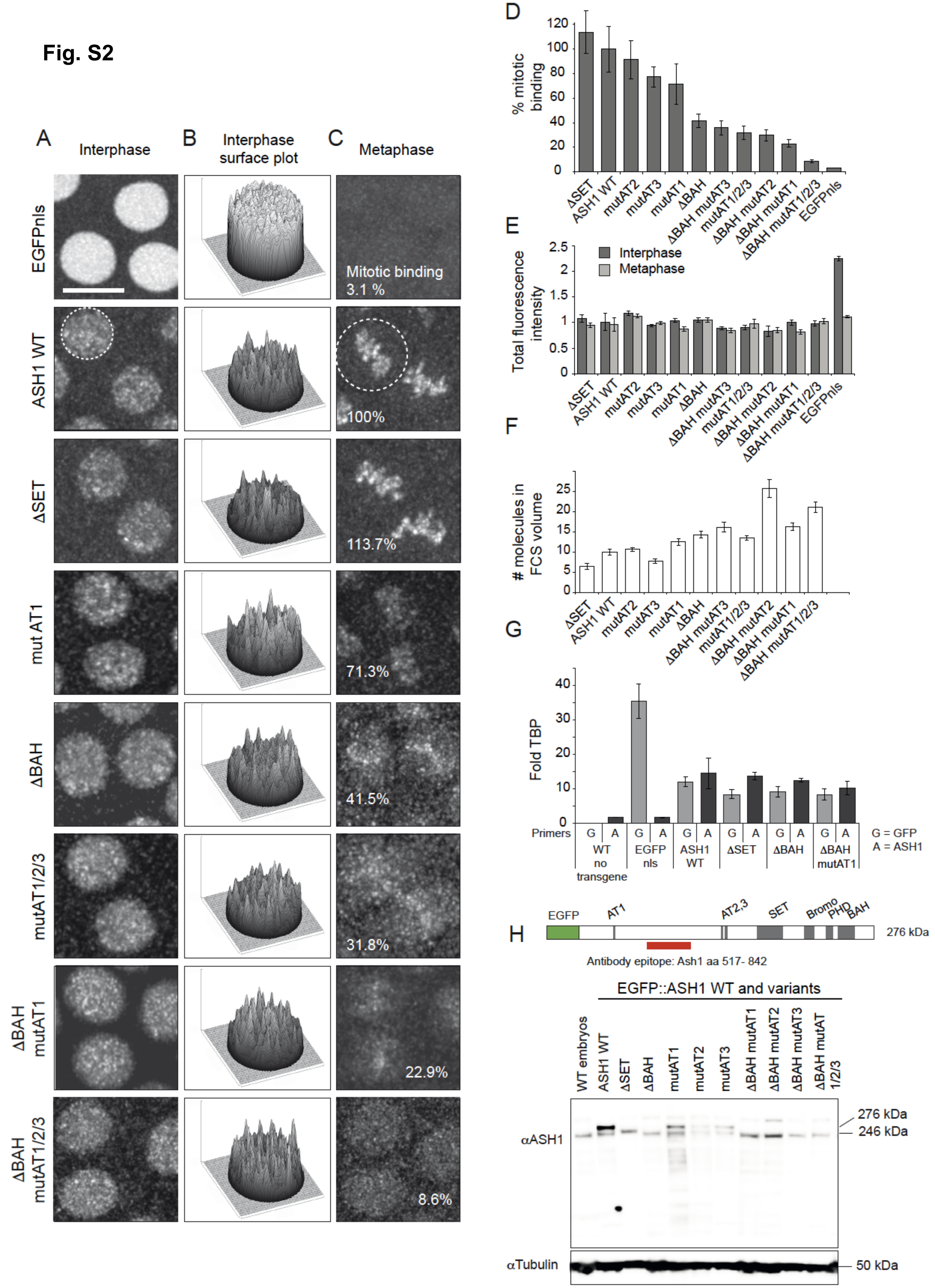
A
Interphase
B
Interphase surface plot
C
Metaphase
EGFPnls
ASH1 WT
ΔSET
mut AT1
ΔBAH
mutAT1/2/3
ΔBAH mutAT1
ΔBAH mutAT1/2/3
Mitotic binding 3.1 %
100%
113.7%
71.3%
41.5%
31.8%
22.9%
8.6%
D
% mitotic binding
0
20
40
60
80
100
120
ΔSET
ASH1 WT
mutAT2
mutAT3
mutAT1
ΔBAH
ΔBAH mutAT3
mutAT1/2/3
ΔBAH mutAT2
ΔBAH mutAT1
ΔBAH mutAT1/2/3
EGFPnls
E
Total fluorescence intensity
Interphase
Metaphase
0
0.5
1
1.5
2
2.5
F
molecules in FCS volume
0
5
10
15
20
25
G
Fold TBP
0
10
20
30
40
Primers
G
A
WT no transgene
EGFP nls
ASH1 WT
ΔSET
ΔBAH
ΔBAH mutAT1
G = GFP
A = ASH1
H
EGFP
AT1
AT2,3
SET
Bromo
PHD
BAH
276 kDa
Antibody epitope: Ash1 aa 517- 842
EGFP::ASH1 WT and variants
WT embryos
ASH1 WT
ΔSET
ΔBAH
mutAT1
mutAT2
mutAT3
ΔBAH mutAT1
ΔBAH mutAT2
ΔBAH mutAT3
ΔBAH mutAT 1/2/3
αASH1
276 kDa
246 kDa
αTubulin
50 kDa

Fig. S3 ASH1 mitotic chromatin attachment is independent of FSH
A) EGFP::FSH-S fusion protein. Grey: domains according to UNIPROT; green: EGFP tag. B) Clustal Omega alignment of the bromodomains of mammalian BRD2, BRD3, BRD4, BRDT and Drosophila FSH-S. Upper panel: first bromodomain, lower panel: second bromodomain. Conserved PFV residues which were mutated into alanines in the EGFP::FSH-S PFV1/2 fusion proteins are highlighted in red. These mutations are predicted to interfere with the binding to acetyl lysines due to alterations in the structure of the bromodomain (Morinière et al., 2009; Pivot-Pajot et al., 2003). Conservation of residues is shown for the first and second bromodomains individually above each alignment and for both bromodomains below the second alignment. JQ1 contact: Residues shown to be responsible for formation of the binding pocket that is occupied by the BET-family inhibitor JQ1 are marked by "^" according to the alignment in (Filippakopoulos et al., 2010). C) Interphase chromatin binding kinetics were measured by FCS in preblastoderm embryos during cleavage cycles 10-13. Kinetic association/dissociation constants, the diffusion coefficient and the abundance of the proteins in the measurement volume were extracted from fits of a reaction-diffusion model to experimental FCS autocorrelation curves as described in (Steffen et al., 2013) and are given in . C) Top: residence times were calculated as $1/k_{off}$ from the mean dissociation constant shown in . Bottom: Bound fractions were calculated from mean values of k^*_{on} and k_{off} shown in , as $k^*_{on}/(k^*_{on} + k_{off})$. Error bars represent cumulative standard error of measurements in 5 nuclei per experiment. Statistical significance was tested using ANOVA with a Dunnett post-test (α = 0.05) comparing FSH-S PFV1 and 2 to FSH-S WT. *p* - values for ANOVA: Residence time: 0.0043; Bound fraction: 0.8393. D) Living blastoderm embryos were imaged using time-lapse confocal microscopy to visualize expression and localization of FSH-S WT and the variants indicated, during cleavage cycles 10-13 of embryogenesis. Individual timepoints corresponding to different cell-cycle phases shown. Images are maximum-intensity projections of z-stacks from deconvolved time-lapse experiments. Scale bar represents 10 µm and is the same for all images. E) Averaged profiles were measured as for Figure 1. The FSH-S WT profile is shown in red in the other panels as reference. F,G) Embryos expressing EGFP::FSH-S (F) or EGFP::ASH1 (G) were injected with (+)-JQ1. Chromatin was visualized by co-injection of Hoechst 34580. Individual de-convolved planes of z-stacks from time-lapse microscopy of living embryos are shown. Green: EGFP fusion protein, magenta: DNA marked by Hoechst 34580. Scale bar = 10 µm. H) Interphase residence times (top) and bound fractions (bottom) for EGFP::ASH1 upon mock injection or injection of (+)-JQ1 were measured by FCS as described in (C) above. For full set of parameters see Table S1. Error bars represent cumulative standard error of measurements in 5 (mock injected) and 8 (JQ1 injected) nuclei per experiment.

Fig. S3

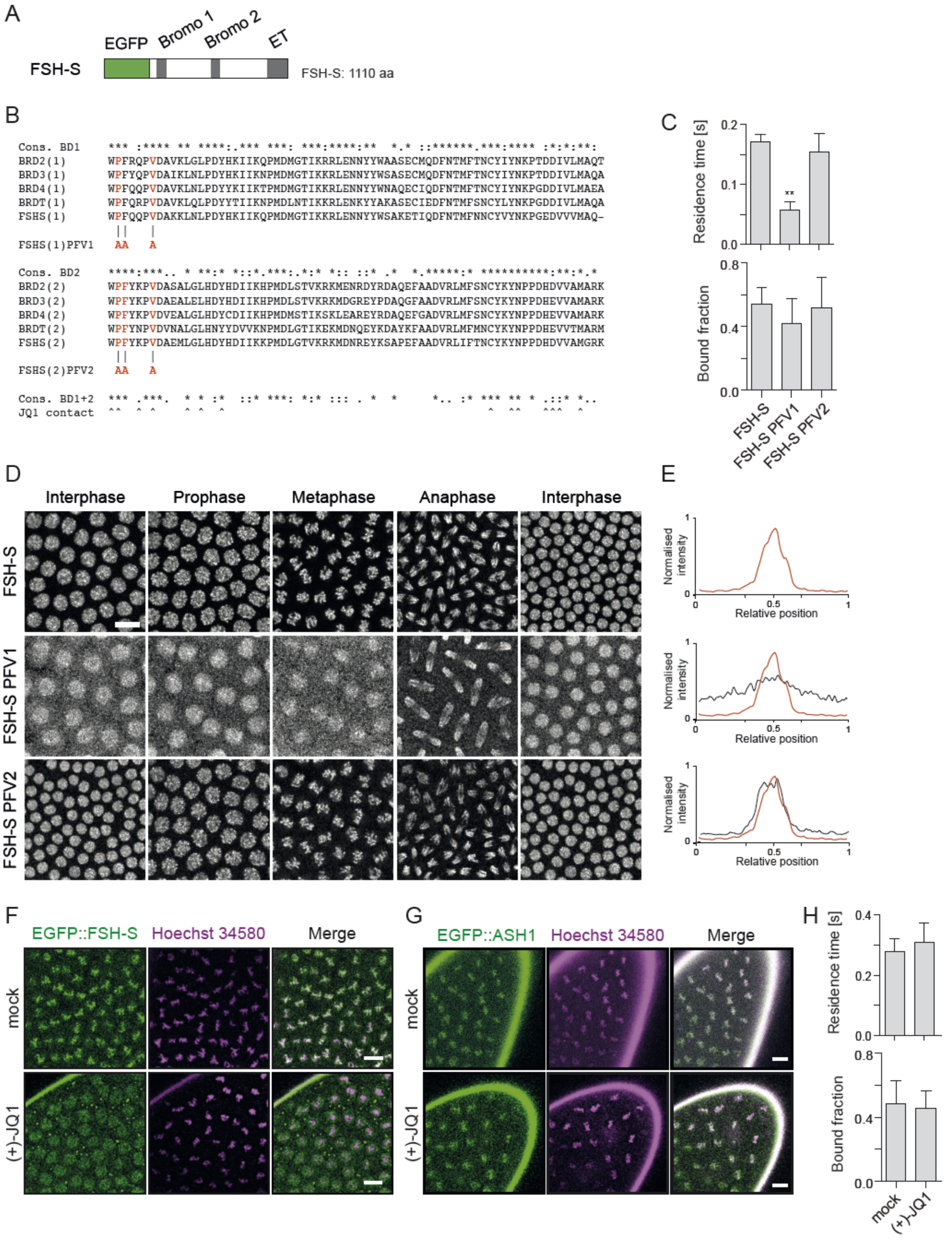
A
EGFP
Bromo 1
Bromo 2
ET
FSH-S
FSH-S: 1110 aa
B
Cons. BD1 *** :**** ** ****:.*** ***: ***:****:** *.* ::******.***:**** :*:*:**:
BRD2(1) WPFRQPVDAVKLGLPDYHKIIKQPMDMGTIKRRLENNYYWAASECMQDFNTMFTNCYIYNKPTDDIVLMAQT
BRD3(1) WPFYQPVDAIKLNLPDYHKIIKNPMDMGTIKKRLENNYYWSASECMQDFNTMFTNCYIYNKPTDDIVLMAQA
BRD4(1) WPFQQPVDAVKLNLPDYYKIIKTPMDMGTIKKRLENNYYWNAQECIQDFNTMFTNCYIYNKPGDDIVLMAEA
BRDT(1) WPFQRPVDAVKLQLPDYYTIIKNPMDLNTIKKRLENKYYAKASECIEDFNTMFSNCYLYNKPGDDIVLMAQA
FSHS(1) WPFQQPVDAKKLNLPDYHKIIKQPMDMGTIKKRLENNYYWSAKETIQDFNTMFNNCYVYNKPGEDVVVMAQ-
FSHS(1)PFV1 AA A
Cons. BD2 ****:***.. * **:* *::*.***:.*:* *:: ::* .* *.*****:* **********:**:*.*
BRD2(2) WPFYKPVDASALGLHDYHDIIKHPMDLSTVKRKMENRDYRDAQEFAADVRLMFSNCYKYNPPDHDVVAMARK
BRD3(2) WPFYKPVDAEALELHDYHDIIKHPMDLSTVKRKMDGREYPDAQGFAADVRLMFSNCYKYNPPDHEVVAMARK
BRD4(2) WPFYKPVDVEALGLHDYCDIIKHPMDMSTIKSKLEAREYRDAQEFGADVRLMFSNCYKYNPPDHEVVAMARK
BRDT(2) WPFYNPVDVNALGLHNYYDVVKNPMDLGTIKEKMDNQEYKDAYKFAADVRLMFMNCYKYNPPDHEVVTMARM
FSHS(2) WPFYKPVDAEMLGLHDYHDIIKKPMDLGTVKRKMDNREYKSAPEFAADVRLIFTNCYKYNPPDHDVVAMGRK
FSHS(2)PFV2 AA A
Cons. BD1+2 *** .***. * * :* ::* ***: *:* ::: . * * *.. :* *** ** * .::* *..
JQ1 contact ^^ ^ ^ ^ ^ ^ ^ ^^ ^^^ ^
C
Residence time [s]
0.2
0.1
0.0
**
Bound fraction
0.8
0.4
0.0
FSH-S
FSH-S PFV1
FSH-S PFV2
D
Interphase
Prophase
Metaphase
Anaphase
Interphase
FSH-S
FSH-S PFV1
FSH-S PFV2
E
Normalised intensity
1
0
0.5
Relative position
F
EGFP::FSH-S
Hoechst 34580
Merge
mock
(+)-JQ1
G
EGFP::ASH1
Hoechst 34580
Merge
mock
(+)-JQ1
H
Residence time [s]
0.4
0.2
0.0
Bound fraction
0.8
0.4
0.0
mock
(+)-JQ1

Fig. S4 Comparison of RNA-seq and qPCR data

The left side of each plot shows the mean RPKM (reads per kilobase per million) and standard deviation (SD) of three replicates as evaluated by RNA-seq. Each replicate is an independent RNA preparation from 3rd instar larval wing discs of the genotypes shown. *p* – values for the relevant differences, calculated on the basis of the SD between replicates (t - test) from RNAseq data as described in supplementary material (RNA_seq), are shown. The right side of each plot shows RT-qPCR analysis of the same RNAs as % TBP (TATA binding protein mRNA). Mean and standard deviation of at least two biological replicates for each genotype are shown.

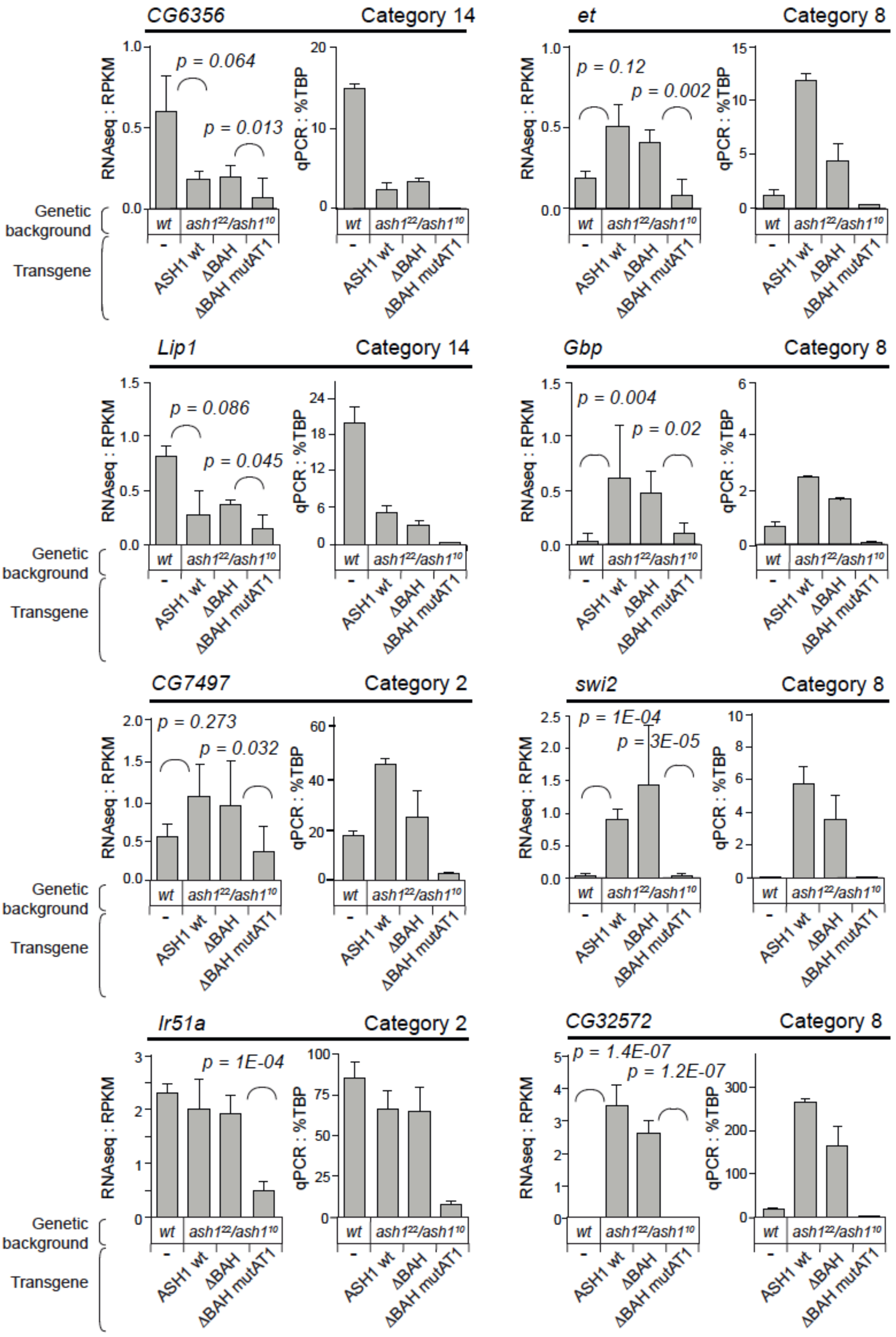

Fig. S5 RNA seq data

A) Summary of RNA-seq data from 3rd instar larval wing discs showing genes whose mean RPKM changes >2- fold between the two genotypes shown at the top of the scheme, after filtering out short genes and lowly expressed genes (see methods and Figure 4). E.g., “up” in column 1 indicates that the genes in this group are more highly expressed in EFGP::ASH1 WT; *ash1*^{10} / *ash1*^{22} than in wild type wing discs. Categories are indicated in circles. Total gene numbers in each category are given, without *p* - value filtering. (For *p* - value filtering see Figure 4). B) Heat map of expression levels for the three replicates for all genes in each category are shown. Red: high expression; green: low expression.

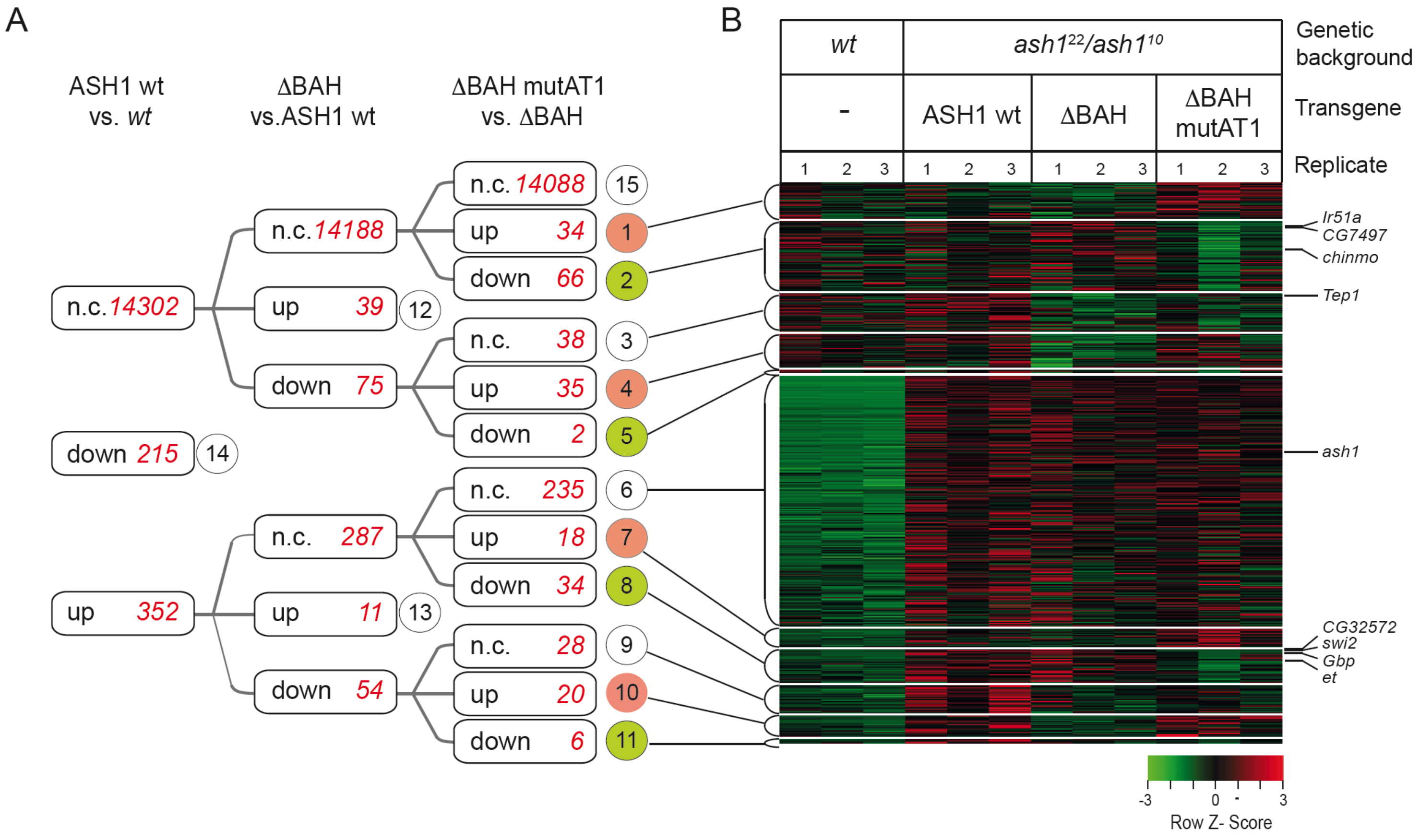

Table S1 Interphase chromatin binding kinetics determined by Fluorescence correlation spectroscopy (FCS)
Compilation of measured chromatin binding kinetics in interphase of *Drosophila* pre-blastoderm embryos. FCS experiments were performed in several nuclei (n is shown for each protein in the table) and analyzed individually as described in (Steffen et al., 2013). Extracted parameters from reaction-diffusion model fits were averaged. The Table shows the mean values ± standard error for the diffusion coefficients (*Df* [$\mu m^2 s^{-1}$]), the pseudo-first order association rate constant k^*_{on} [s^{-1}], the dissociation rate constant k_{off} [s^{-1}] and the absolute number of fusion protein molecules in the FCS measurement volume. The residence time [s] was calculated as $1/k_{off}$ from the mean dissociation rate. The fraction of bound molecules was calculated as $k^*_{on}/(k_{off} + k^*_{on})$ from mean rate constants. The table contains FCS interphase chromatin binding kinetics of wt ASH1::GFP as given in (Steffen et al., 2013) for comparison.

Protein	Nuclei	Df [$\mu m^2 s^{-1}$]	k^*_{on} [s^{-1}]	k_{off} [s^{-1}]	Number of molecules in FCS volume	Residence time [s]	bound fraction
**ASH1**	34	4.98 ± 0.36	6.07 ± 0.53	5.81 ± 0.35	9.97 ± 0.72	0.17 ± 0.01	0.51 ± 0.05
**ASH1ΔSET**	17	6.45 ± 0.81	7.12 ± 0.79	6.89 ± 0.48	6.47 ± 0.82	0.15 ± 0.01	0.51 ± 0.07
**ASH1 mutAT1**	23	4.45 ± 0.31	6.16 ± 0.54	7.22 ± 0.60	12.52 ± 0.83	0.14 ± 0.01	0.46 ± 0.06
**ASH1 mutAT2**	11	5.51 ± 0.67	6.80 ± 0.70	6.34 ± 0.49	10.59 ± 0.46	0.16 ± 0.01	0.52 ± 0.07
**ASH1 mutAT3**	13	5.48 ± 0.52	6.38 ± 0.87	6.93 ± 0.96	7.76 ± 0.47	0.14 ± 0.02	0.48 ± 0.09
**ASH1 mutAT1/2/3**	29	5.16 ± 0.57	7.93 ± 0.80	7.86 ± 0.60	13.50 ± 0.58	0.13 ± 0.01	0.50 ± 0.06
**ASH1ΔBAH**	26	3.12 ± 0.57	5.28 ± 0.89	8.79 ± 1.04	14.24 ± 0.82	0.11 ± 0.01	0.38 ± 0.08
**ASH1ΔBAH mutAT1**	15	2.63 ± 0.36	7.33 ± 2.03	14.21 ± 2.90	16.31 ± 0.94	0.07 ± 0.01	0.34 ± 0.12
**ASH1ΔBAH mutAT2**	20	2.60 ± 0.21	4.95 ± 0.76	10.31 ± 0.98	25.73 ± 2.33	0.10 ± 0.01	0.32 ± 0.06
**ASH1ΔBAH mutAT3**	15	2.59 ± 0.18	6.39 ± 1.12	11.72 ± 1.13	16.12 ± 1.22	0.09 ± 0.01	0.35 ± 0.07
**ASH1ΔBAH mutAT1/2/3**	15	3.36 ± 0.56	13.22 ± 3.20	21.66 ± 3.54	21.07 ± 1.32	0.05 ± 0.01	0.38 ± 0.11
**FSH-S**	5	1.94 ± 0.48	6.95 ± 1.21	5.90 ± 0.45	15.65 ± 1.79	0.17 ± 0.01	0.54 ± 0.10
**FSH-S PFV1**	5	7.07 ± 1.63	12.54 ± 3.65	17.38 ± 4.07	5.83 ± 0.61	0.06 ± 0.01	0.42 ± 0.16
**FSH-S PFV2**	5	4.24 ± 1.08	7.00 ± 2.17	6.48 ± 1.26	16.16 ± 1.07	0.15 ± 0.03	0.52 ± 0.19
**ASH1 mock injection**	5	2.95 ± 0.24	3.32 ± 0.89	3.59 ± 0.48	5.52 ± 0.65	0.28 ± 0.04	0.48 ± 0.14
**ASH1 JQ1 injection**	8	3.48 ± 1.56	2.69 ± 0.42	3.14 ± 0.48	10.59 ± 0.88	0.32 ± 0.05	0.46 ± 0.10

Table S2 Filtered RNA-seq data (see Excel sheet Table S2)

RNA was extracted from 3rd instar larval wing discs and RNA-seq was performed on 3 biological replicates for each genotype. *Sheet 1, All Dereg genes.* Columns A-D: Information on gene identities. Column E: Category. 14869 genes were evaluated, and each was assigned a category according to its expression pattern across the four genotypes. For categories 1- 15, see Figure 4F. Additional categories shown in the table: 16: Lowly expressed genes. Genes for which the sum of mean RPKMs (reads per kb per million) across all four genotypes is less than 1 (column J). 17: Short genes. Inspection of tracks showed that these genes have short ORFs and thus artificially high RPKMs. This category consists of tRNA genes, snoRNAs, snRNAs, and small non messenger RNAs. Column F-I: Mean RPKM values of the three replicates for each genotype are shown. WT: Wild type. nAT: expression of full length EGFP::ASH1 in *ash1^{22}/ash^{10}* background. dBAH: expression of EGFP::ΔBAH ASH1 in *ash1^{22}/ash^{10}* background. dBAHmut1: expression of EGFP:: ΔBAH mutAT1 ASH1 in *ash1^{22}/ash^{10}* background. Column F: Sum of values in columns F-I, used to filter out lowly expressed genes (category 16). Columns K-N: Standard deviation of the RPKM values of the three replicates for each genotype. O-S; T-X; Y-AC: pairwise comparisons. Column O: Fold change of nAT vs WT. Calculated from normalised count values. Positive values indicate that mean RPKM is higher in nAT than in WT. Negative values indicate lower mean RPKM in nAT than WT. Column T: Fold change of dBAH vs nAT (+ve, dBAH up; -ve, dBAH down). Column Y: Fold change of dBAHmut1 vs dBAH (+ve, dBAHmut1 up; -ve, dBAHmut1 down). Columns P, U, Z: Log fold change is shown as log2. Columns Q, V, AA: *p* - value based on the SD (t - test). Columns R, W, AB: FDR adjusted *p* - value. Columns S, X, AC: Rank of the fold change. Combines the fold change itself and the *p* - value. Lowest number has the highest rank. *Sheet 2, Overlap Schmäling*. Overlaps between selected categories in our analysis and the data set of Schmäling et al., 2018, as indicated in the Table.

Table S3 Raw RNA-seq data (see Excel sheet Table S3)

RNA was extracted from 3rd instar larval wing discs and RNA-seq was performed on 3 biological replicates for each genotype. Whole data set without filetering as in Table S2 is given. Column A: Gene Identifier. Column B-E (black): Mean RPKM values of the three replicates for each genotype are shown. WT: Wild type. nAT: expression of full length EGFP::ASH1 in *ash1^{22}/ash^{10}* background. dBAH: expression of EGFP::ΔBAH ASH1 in *ash1^{22}/ash^{10}* background. dBAHmut1: expression of EGFP:: ΔBAH mutAT1 ASH1 in *ash1^{22}/ash^{10}* background. Column F: Sum of values in columns B-E, used to filter out lowly expressed genes in Table 2. Columns G-J (blue): Standard deviation of the RPKM values of the three replicates for each genotype. K-M; N-P; Q-S: pairwise comparisons. See legend to Table S2 for details and *p* - values. Columns T-V: Information on gene identities.

Supplementary references

Bischof, J., Björklund, M., Furger, E., Schertel, C., Taipale, J. and Basler, K. (2013). A versatile platform for creating a comprehensive UAS-ORFeome library in Drosophila. *Development (Cambridge, England)*.

Culbertson, C. T., Jacobson, S. C. and Michael Ramsey, J. (2002). Diffusion coefficient measurements in microfluidic devices. *Talanta* **56**, 365-73.

Filippakopoulos, P., Qi, J., Picaud, S., Shen, Y., Smith, W. B., Fedorov, O., Morse, E. M., Keates, T., Hickman, T. T., Felletar, I. et al. (2010). Selective inhibition of BET bromodomains. *Nature* **468**, 1067-1073.

Heckman, K. L. and Pease, L. R. (2007). Gene splicing and mutagenesis by PCR-driven overlap extension. *Nat Protoc* **2**, 924-32.

Morinière, J., Rousseaux, S., Steuerwald, U., Soler-López, M., Curtet, S., Vitte, A.-L., Govin, J., Gaucher, J., Sadoul, K., Hart, D. J. et al. (2009). Cooperative binding of two acetylation marks on a histone tail by a single bromodomain. *Nature* **461**, 664-668.

Okulski, H., Druck, B., Bhalerao, S. and Ringrose, L. (2011). Quantitative analysis of polycomb response elements (PREs) at identical genomic locations distinguishes contributions of PRE sequence and genomic environment. *Epigenetics & chromatin* **4**, 4.

Pivot-Pajot, C., Caron, C., Govin, J., Vion, A., Rousseaux, S. and Khochbin, S. (2003). Acetylation-dependent chromatin reorganization by BRDT, a testis-specific bromodomain-containing protein. *Molecular and cellular biology* **23**, 5354-5365.

Ringrose, L. (2009). Transgenesis in Drosophila melanogaster. *Methods in molecular biology (Clifton, NJ)* **561**, 3-19.

Steffen, P. A., Fonseca, J. P., Gänger, C., Dworschak, E., Kockmann, T., Beisel, C. and Ringrose, L. (2013). Quantitative in vivo analysis of chromatin binding of Polycomb and Trithorax group proteins reveals retention of ASH1 on mitotic chromatin. *Nucleic acids research* **41**, 5235-5250.
