# Supplementary material for: The Trithorax group protein ASH1 requires a combination of BAH domain and AT hooks, but not the SET domain, for mitotic chromatin binding and survival
Source: Chromosoma. 2021 Jul 31;130(2-3):215–34. doi: 10.1007/s00412-021-00762-z (PMC8426247; doi:10.1007/s00412-021-00762-z)
Supplement: Supplementary file 2 — Supplementary file2 (PDF 1054 KB) [file 412_2021_762_MOESM2_ESM.pdf]

Gene expression profiling study for mutant *ash1* gene in *Drosophila* using RNA-seq

Sini Junttila, Attila Gyenesei
sini.junttila@vbcf.ac.at
attila.gyenesei@vbcf.ac.at

BioComp
Vienna Biocenter Core Facilities
http://www.vbcf.ac.at/facilities/bioinformatics-and-scientific-computing/

Contents

1 Analysis work flow 3

2 Samples 5

3 Data preprocessing and normalization 6

3.1 HiSeq sequencing run information 6

3.2 Aligning reads to the reference genome 6

3.3 Calculating normalized gene counts 6

3.4 Files for genome browsers 8

4 Data quality control 9

4.1 Expression values 9

4.2 Inspection of the expression value distribution: box plots 10

4.3 Sample relations 12

4.3.1 Correlations 12

4.3.2 Hierarchical clustering 14

4.3.3 PCA 16

4.4 Summary of the sample quality inspections 16

5 Group comparisons 18

5.1 Filtering parameters 18

5.2 Filtering result files 20

5.3 Visualization of the DE features 20

5.4 DE list comparison 40

1 Analysis work flow

The main steps of the analysis of gene expression data are presented in Figure 1. The first step is aligning the transcriptome sequence reads to the reference genome and counting the number of reads for each gene (Section 3). This is followed by the general data inspection (Section 4) during which the general data quality and sample relations are being studied using various measures and visualizations. If low quality samples are detected they may be excluded from further analysis at this point. The data are also normalized (Section 3) to reduce systematic noise caused by non-biological sources and to improve the comparability of the samples.

After the preprocessing, statistical testing is performed between the compared sample groups (Section 5). The results from the testing are used to filter the so called *DE* or *differentially expressed* genes. The filtering is based on the statistical significance and the size of the difference in the mean expression levels between the sample groups (Section 5.1). Annotated result files for all genes and for the filtered gene lists are included in the results (Section 7).

Functional analysis is a term used for describing all of the advanced analyses where the information related to the gene functions is taken into account during the analysis (Section 6). These analyses can be run for the filtered DE gene lists or the whole data to detect enrichments in various functional categories. One functional analysis for both filtered DE gene lists and the whole data has been performed in this data analysis. Input files and usage instructions for two other commonly used enrichment analysis tools have also been included in the report (see 6.3 for more information). The usage of these additional functional analysis tools requires good knowledge of biological background of the experimental set up and thus the purpose of this report is to provide comprehensive support and guidance for the independent use of these tools.

Various statistical methods are used for the analysis of gene expression profiling data. These methods and the related technology will be thoroughly explained in the report so that the results will be interpretable even without deep knowledge in statistics.

All the analyses have been performed with *R language and environment for statistical computing* [7]. Many of the analyses have been performed using R related *Bioconductor* module [1].

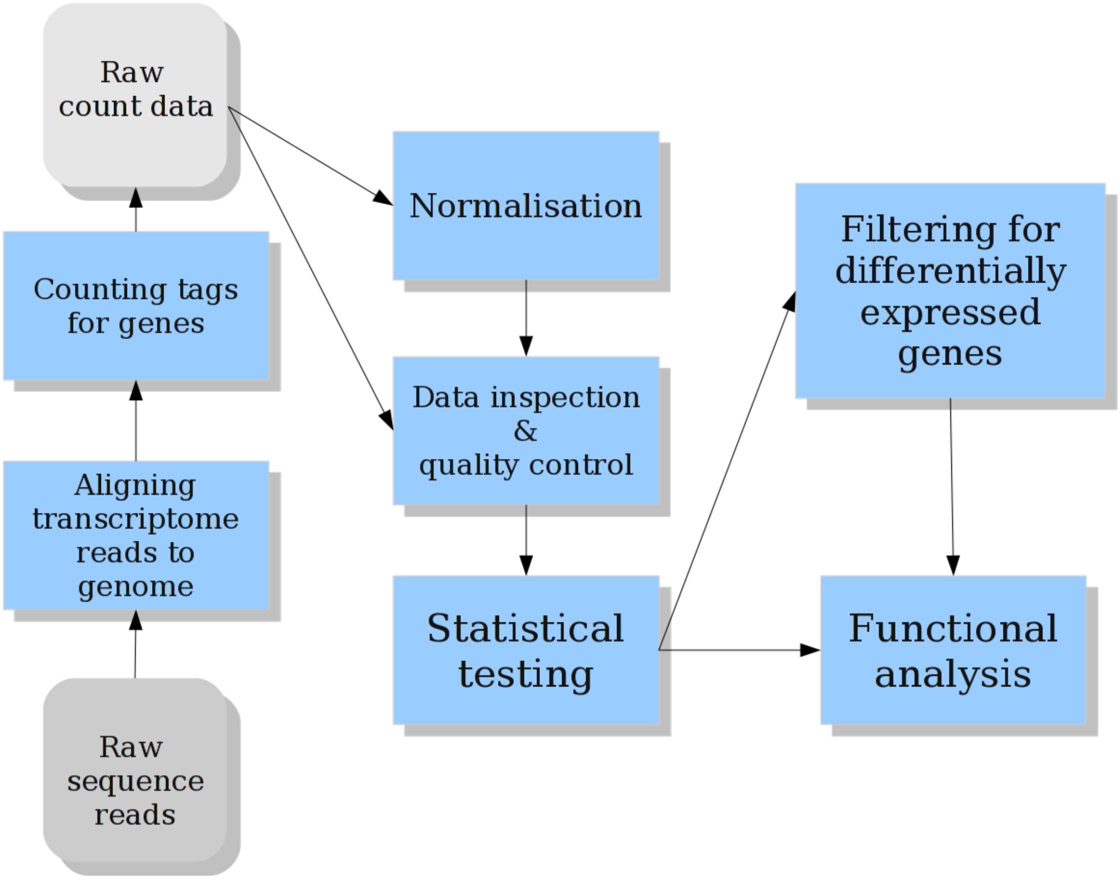

Figure 1: General RNA-seq data analysis workflow

2 Samples

General description of each sample group is given in the table below.

Group name	Number of replicates	Group definition
WT	3	wild type
nAT	3	nAT rescue
dBAH	3	dBAH rescue
dBAHmut1	3	dBAH mut 1 rescue

3 Data preprocessing and normalization

3.1 HiSeq sequencing run information

The samples were sequenced with the HiSeq2500 instrument using single-end sequencing with 50bp read length. The analysed samples were sequenced in three separate runs with differing amounts of samples multiplexed on one lane.

3.2 Aligning reads to the reference genome

The reads obtained from the instrument were base called using the instrument manufacturer's base calling software. The reads were aligned against the Drosophila melanogaster reference genome (BDGP5.25 assembly) using TopHat version 2.0.9 [4]. Summary of the mapping statistics is provided in Table 1.

1	2	3	4	5	6	7
WT_14968	47,600,524	42,841,022	90.0%	41,720,501	1,120,521	4,759,502
WT_15544	36,689,411	32,625,125	88.9%	31,948,356	676,769	4,064,286
WT_17794	19,967,854	17,733,492	88.8%	17,305,569	427,923	2,234,362
nAT_15546	37,129,370	32,554,892	87.7%	31,778,990	775,902	4,574,478
nAT_17798	22,386,404	19,884,954	88.8%	19,233,877	651,077	2,501,450
nAT_17799	22,610,142	19,077,802	84.4%	18,272,131	805,671	3,532,340
dBAH_15547	34,822,993	30,862,324	88.6%	30,181,002	681,322	3,960,669
dBAH_17796	18,858,256	16,738,787	88.8%	16,236,662	502,125	2,119,469
dBAH_17797	20,711,106	18,322,378	88.5%	17,723,729	598,649	2,388,728
dBAHmut1_14969	43,143,444	38,586,053	89.4%	37,499,641	1,086,412	4,557,391
dBAHmut1_15545	45,091,660	40,129,023	89%	39,243,751	885,272	4,962,637
dBAHmut1_17795	20,789,325	18,419,484	88.6%	17,899,920	519,564	2,369,841

Table 1: Summary of mapping statistics

1: **Sample**
2: **Total**: Number of reads detected for the sample
3: **Mapped reads**: Number of reads mapped to the reference genome
4: **Mapping rate**: Percentage of mapped reads from the total number of reads
5: **Unique reads**: Number of uniquely aligned reads
6: **Non-unique reads**: Number of reads mapped to more than one location in the reference genome
7: **Unmapped reads**: Number of reads not mapped to the reference genome

3.3 Calculating normalized gene counts

After alignment, the reads were associated with known genes based on annotations derived from Ensembl and the number of reads aligned within each gene was counted using HTSeq tool version 0.5.4p3.

The data are normalised to remove variation between samples caused by non-biological reasons and to make the values comparable across the sample set. Here the counts were normalised using the TMM normalisation method of the edgeR R/Bioconductor package (R version 3.0.1, Bioconductor version 2.12). The method takes the variable number of total reads across samples into account by calculating specific scaling factors between the samples.

For statistical testing the data were further log transformed using the voom approach in the limma package. For the visualizations and result files the TMM normalised counts are represented as RPKM values [6], which make the values not only between samples but also between genes comparable by taking the variable gene lengths into account. The following formula is used for calculation of the RPKM values:

$$RPKM = \frac{total\_gene\_reads}{mapped\_reads(millions) * gene\_length(KB)} \quad (1)$$

The distribution of the RPKM values in each sample is presented in Table 3.3. The RPKM value column denotes the RPKM value threshold and the amount of genes that have an RPKM value greater than the threshold is given for each sample.

RPKM value	14968	15544	17794	15546	17798	17799	15547	17796	17797	14969	15545	17795
0	14869	14869	14869	14869	14869	14869	14869	14869	14869	14869	14869	14869
0.125	8729	8421	8365	8580	8552	8676	8509	8458	8614	8792	8368	8771
1	7413	7305	7229	7391	7363	7390	7374	7337	7384	7461	7292	7332
3	6832	6744	6696	6762	6786	6786	6763	6777	6787	6852	6739	6761
5	6456	6361	6341	6341	6435	6407	6328	6422	6403	6490	6326	6386
10	5489	5346	5362	5275	5437	5362	5276	5409	5409	5501	5291	5410
15	4536	4444	4474	4340	4500	4427	4325	4492	4479	4559	4292	4485
25	3241	3071	3138	3064	3121	3063	3038	3151	3106	3184	2987	3157
30	2727	2643	2659	2682	2612	2559	2627	2645	2606	2695	2533	2659
40	2031	1967	1946	2090	1901	1895	2044	1943	1901	1962	1908	1949
50	1564	1547	1517	1658	1468	1442	1614	1490	1450	1522	1472	1520

Table 2: The number of genes with the given RPKM value or higher in each sample.

3.4 Files for genome browsers

Various different genome browsers can be used for viewing the aligned count data in genomic context with a variety of different annotations. Figure 2 shows an example of the data viewed with IGV Genome Browser (**IGV** (Integrative Genome Viewer) **Genome Browser** at the Broad Institute: http://www.broadinstitute.org/igv/)

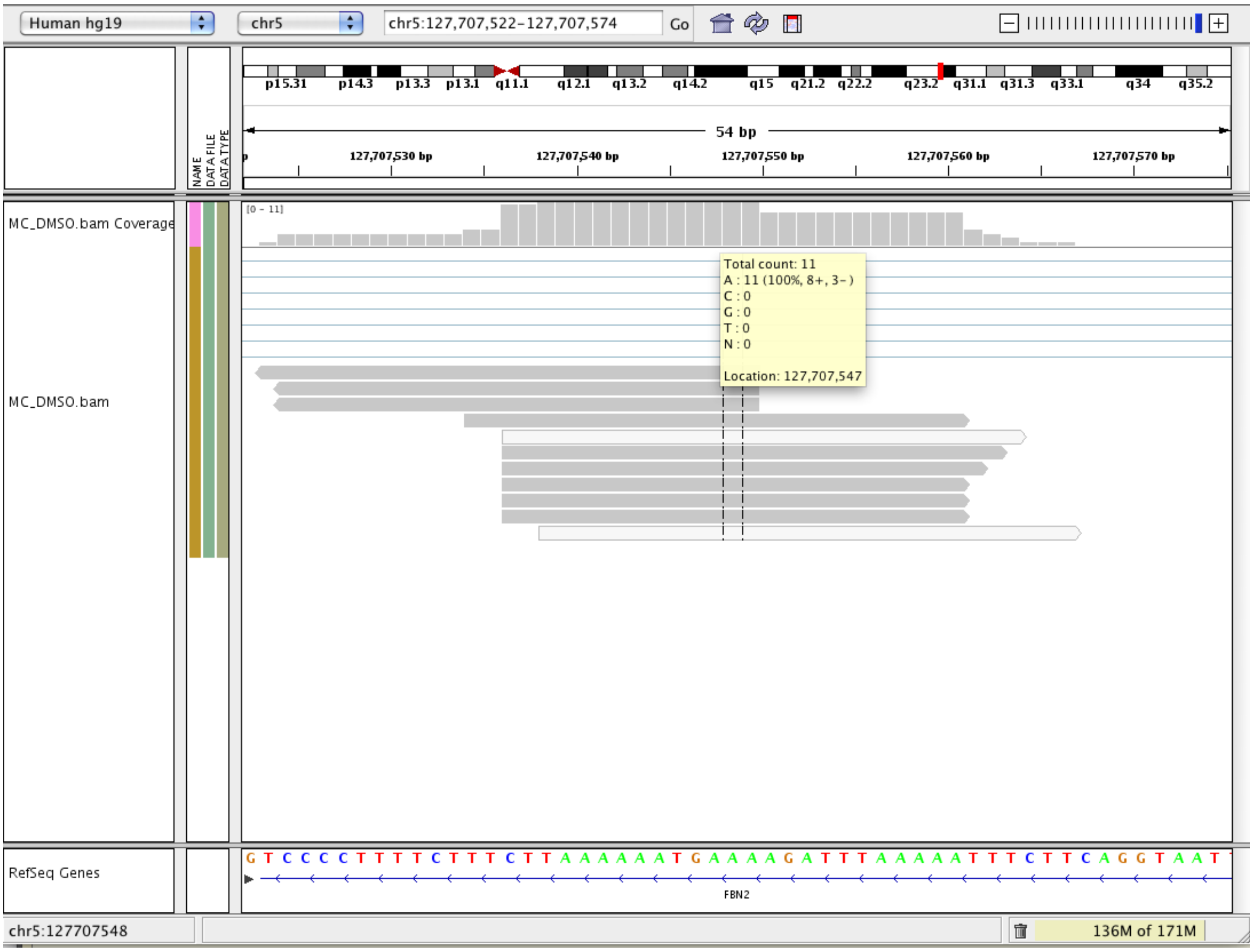

Figure 2: Snapshot from IGV Genome Browser

The *.bam and *.bai files needed for the genome browser are delivered separately by request since these files are very large. IGV genome browser is easy to use and comes with good manuals and tutorials. After choosing the correct genome, the data can be downloaded from File -> 'Load from file...' and choosing the appropriate *.bam and *.bai files.

4 Data quality control

The general quality of the samples and their relationships will be investigated in the following sections.

4.1 Expression values

Figure 3 visualizes the expression value distribution across the sample set.

Table 3 shows the minimum, median, mode, mean and maximum expression values of the normalised samples. The mode can be seen as a rough measure of the background since most of the genes in the genome are not expressed in most experiments.

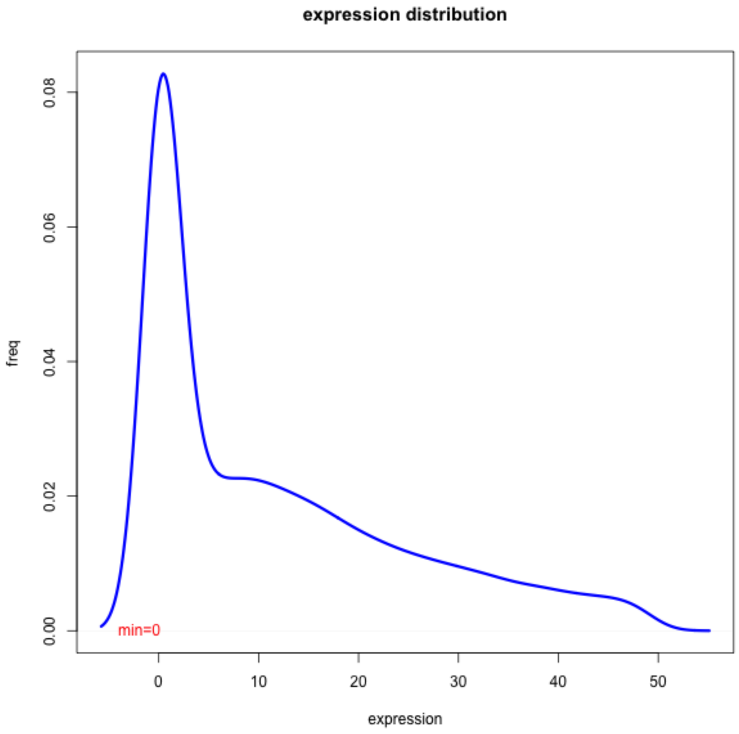

Figure 3: Curve describing a part of the expression value distribution of the samples in this study. Most genes yield very small expression values and only few genes have high values.

Min.	Median	Mean	Max.
0	11	49	38232

Table 3: Sample expression value summary

4.2 Inspection of the expression value distribution: box plots

The expression value distributions across the count values of all samples can be visualized by box plots. In the Figure 5 the asinh-transformed expression value distributions of the samples are visualized as box plots before and after normalization. The structure of a box plot is described in Figure 4.

Large variations in the sample expression value distributions may indicate quality problems. In these data there are some differences which is quite typical. This variation is compensated by the means of normalization after which the expression value distributions of the samples are almost equal. Normalization brings the expression values in to the same register so that the samples can be compared to each other.

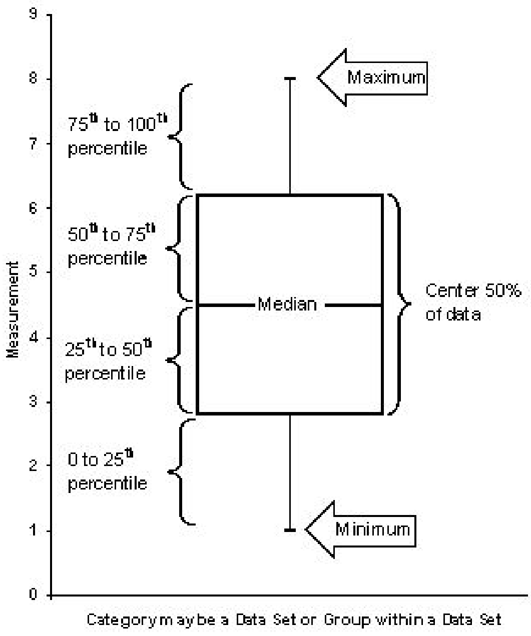

Figure 4: Structure of a box plot

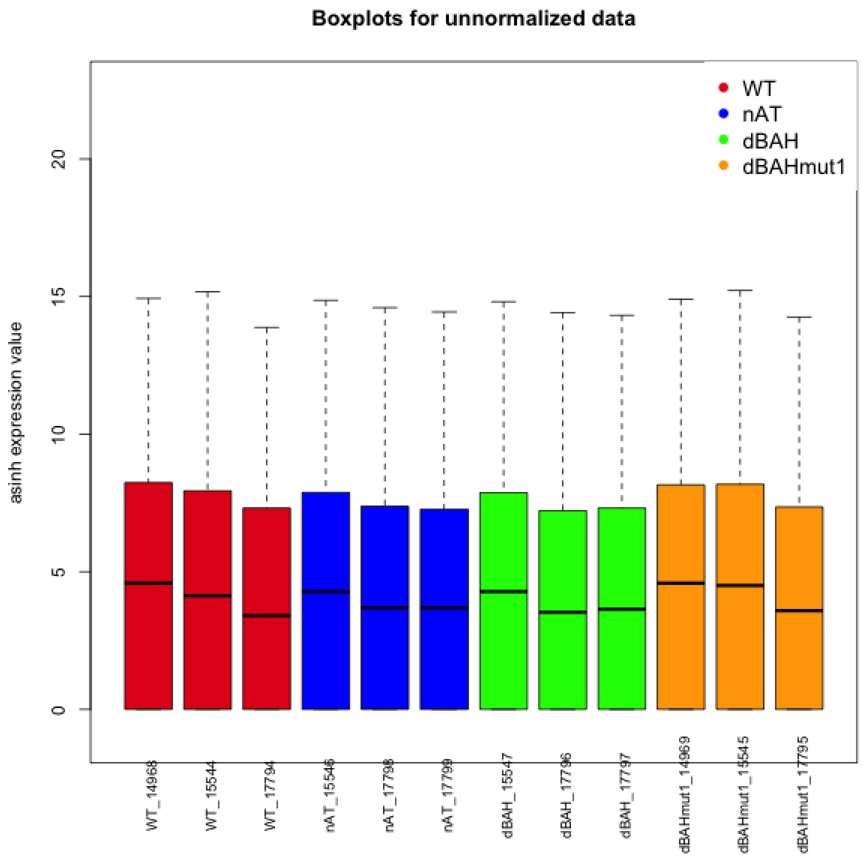

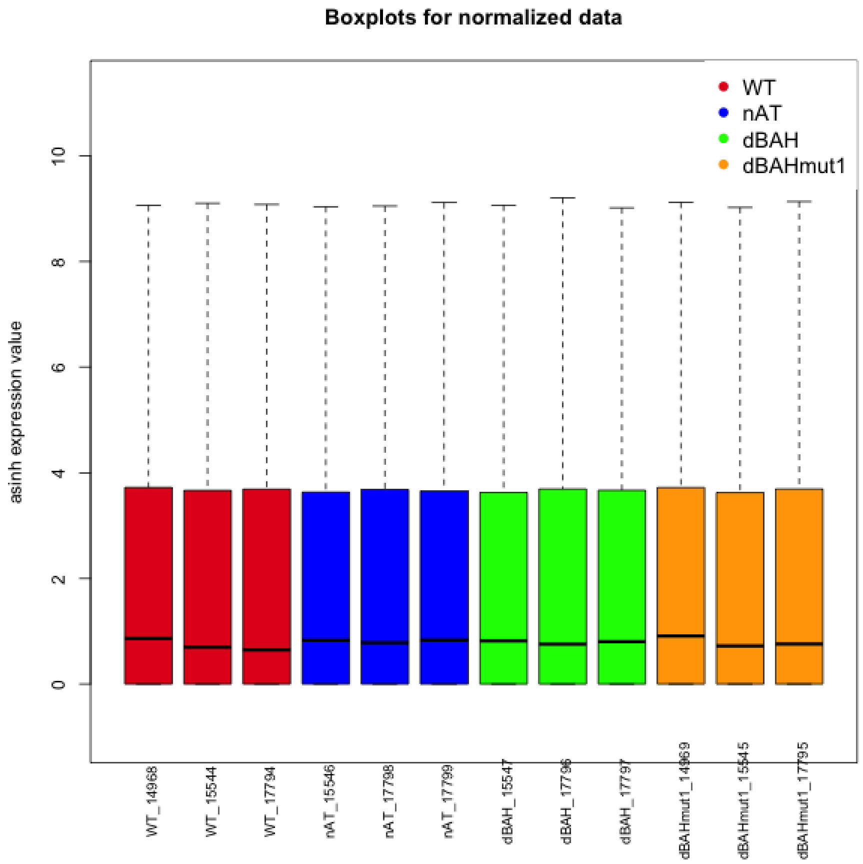

Figure 5: Expression value distributions as box plots for unnormalized and normalized data.

4.3 Sample relations

The sample relationships will be studied in the following sections by the means of correlation and cluster analysis.

4.3.1 Correlations

Between sample correlation values describe the similarity between the samples in a general level, when all measurement features of all samples are taken into consideration. In this analysis the so called *Spearman's metrics* is used which describes the between sample similarity on a scale of 0-1. Value 0 means perfect uncorrelation between the samples where as value 1 means perfect correlation between them. As a rule of thumb, correlation of at least 0.95 is usually expected from samples that are biological replicates.

The correlation values between all possible pairs of samples are visualized in Figure 6. Correlations between samples vary between 0.937 and 0.973. This depicts a high correlation between the samples. Groupwise minimum and mean correlations are given in the table below:

Group	minCor	meanCor
WT	0.959	0.961
nAT	0.937	0.952
dBAH	0.945	0.956
dBAHmut1	0.951	0.955

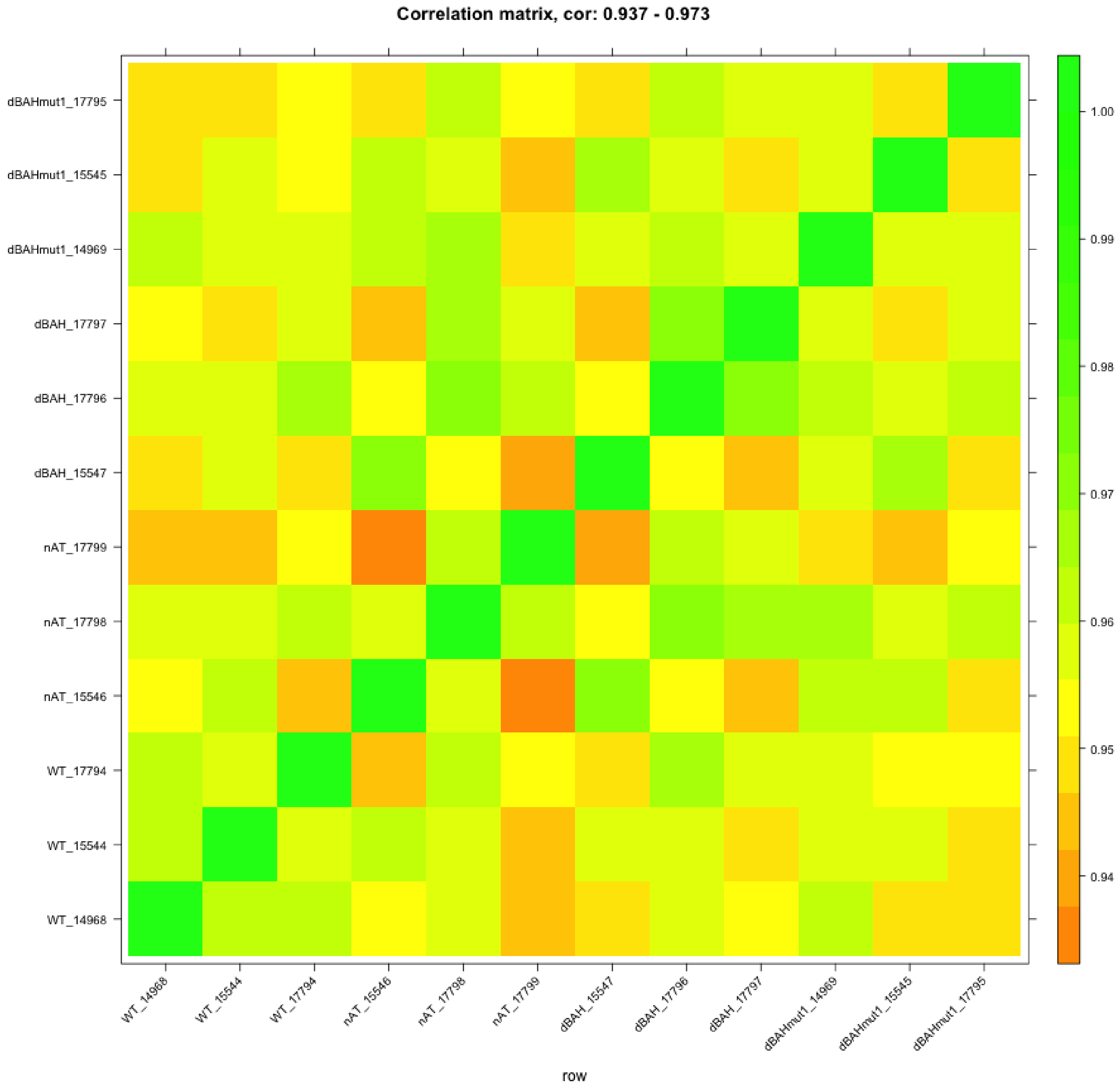

Figure 6: Sample correlations for normalized data.

4.3.2 Hierarchical clustering

In hierarchical clustering the samples are grouped according to their general similarity when all the measurements of all the samples are taken into consideration. In this analysis the samples were clustered with Euclidean metrics.

The result of the cluster analysis can be visualised as a *dendrogram* which is an out branching graph where the most similar samples (in another words best correlating) can be found in the branches that are nearest to one another.

The dendrogram produced by cluster analysis is shown for normalized data in Figure 7.

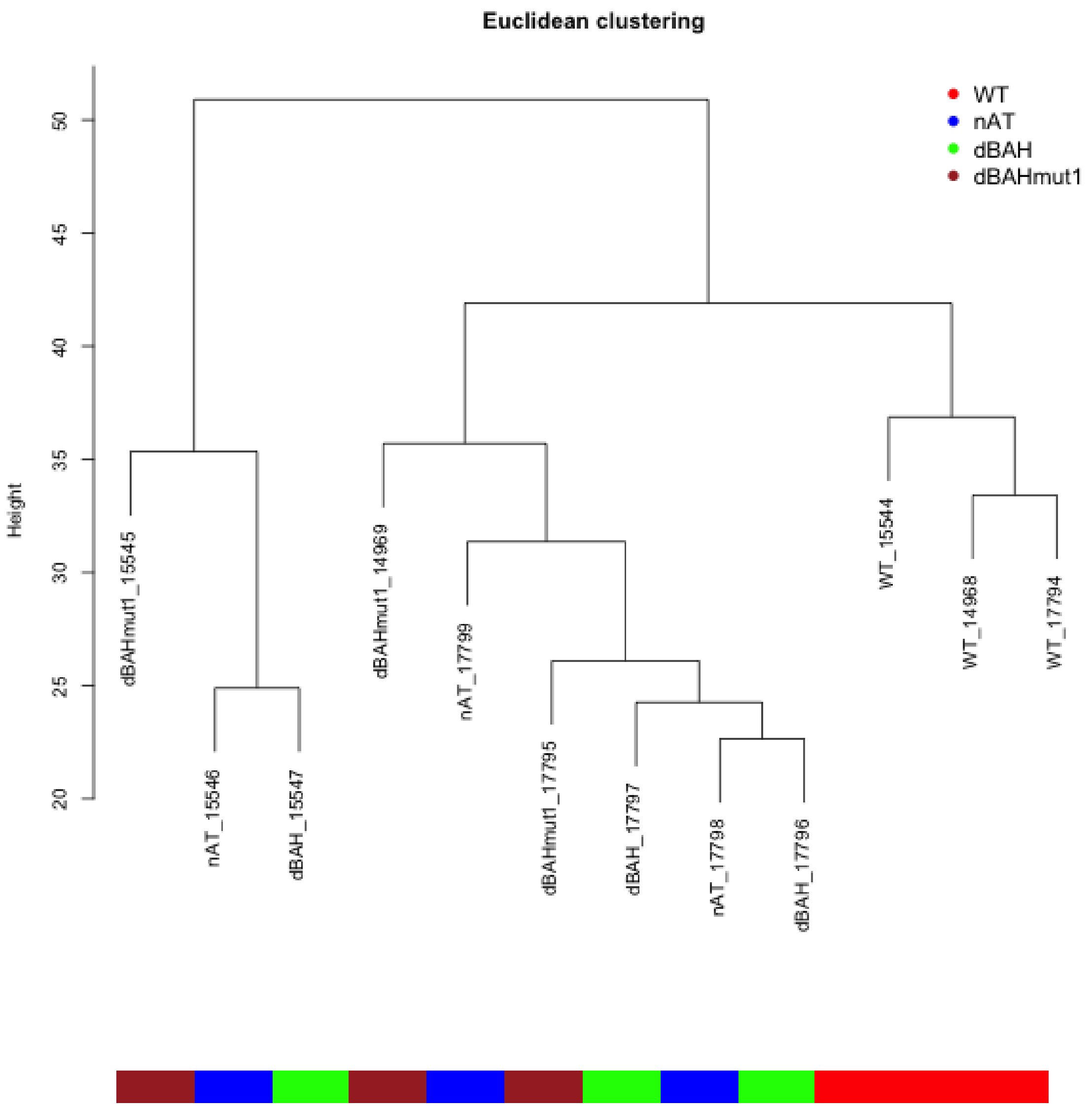

Figure 7: Hierarchical clustering for the normalized data.

In the hierarchical clustering, the WT samples group into a cluster of their own. The other samples group according to the sequencing runs. This is quite expected when clustering whole data and as the correlation values are high, the overall differences between the samples are likely quite small.

4.3.3 PCA

The sample relations can also be studied by the means of a Principal Component Analysis (PCA) which is an ordination technique complementary to clustering. Ordination orders objects so that similar objects are placed near each other and dissimilar objects are placed further from each other.

In PCA analysis the sample relationships can be visualized in three dimensional space. Figure 8 shows the PCA analysis for all samples.

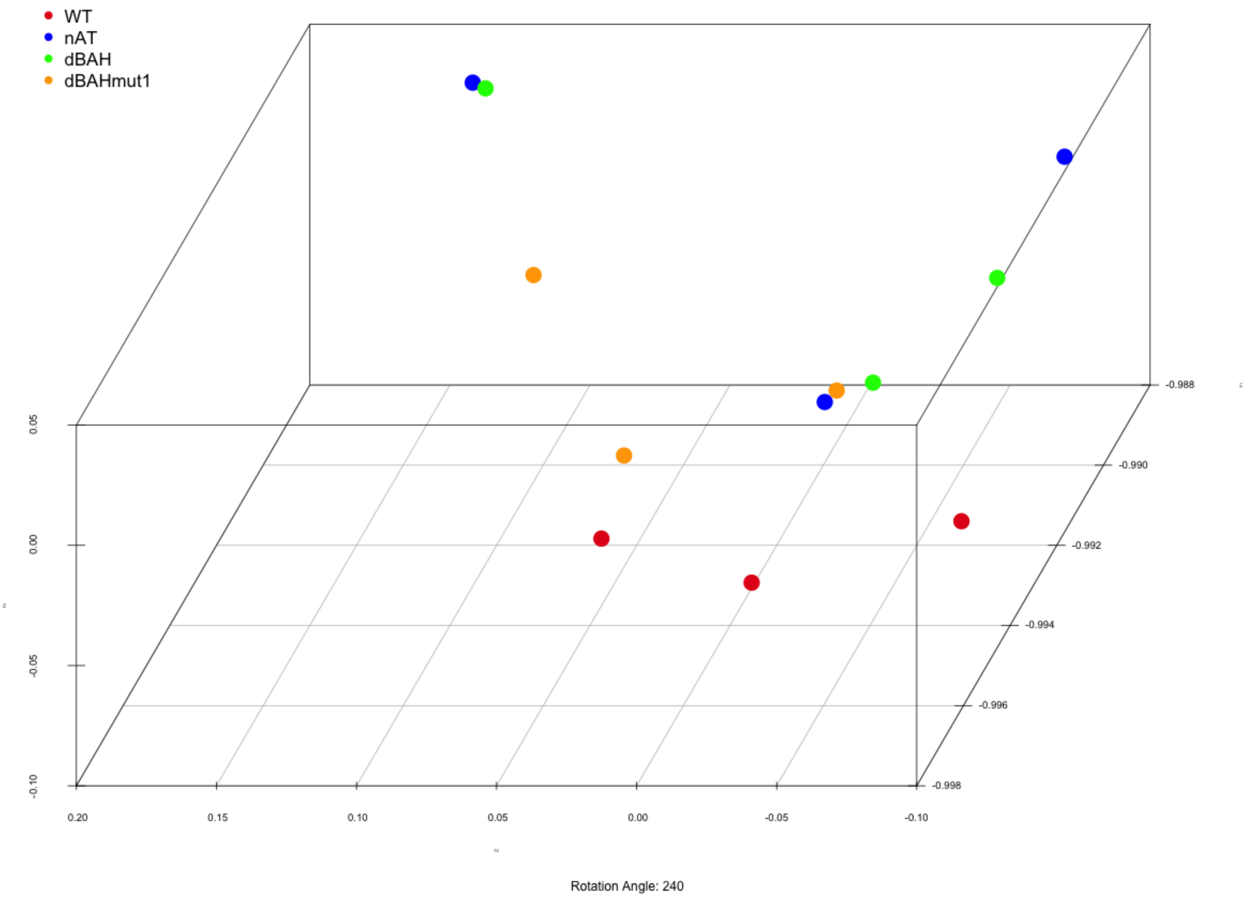

Figure 8: PCA plot for the normalized data.

The samples group similarly in the PCA plot as they did in the clustering dendrogram.

4.4 Summary of the sample quality inspections

Sample correlation values are high and technically the sample data is of high quality.

In the hierarchical clustering the WT sample group clustered in a group of its own and the other samples clustered according to the sequencing run. The PCA gave similar results as the

hierarchical clustering. The correlation values were high and therefore the differences between the samples are likely to be small.

5 Group comparisons

The following comparisons were performed to detect differentially expressed genes between groups:

- nAT rescue vs. WT
- dBAH rescue vs. nAT rescue
- dBAH mut 1 rescue vs. dBAH rescue
- dBAH rescue vs. WT
- dBAH mut 1 rescue vs. WT
- dBAH mut 1 rescue vs. nAT rescue

R package *Limma* [8] was used for performing the statistical testing. More information on the package can also be found in appendix B.

5.1 Filtering parameters

When filtering up- and down-regulated (i.e. differentially expressed = DE) genes between certain conditions (groups) fold changes and p-values (or corrected p-values) calculated during statistical testing are used as filtering criteria.

All of the measured genes are filtered to list those that show the strongest evidence for being differentially expressed between the compared groups.

Short descriptions of fold change and p-values:

FC/logFC Fold change describes the size of the difference in gene expression between the compared groups. In this analysis it results from linear modeling process performed with Limma package (see Appendix B). Fold changes are often expressed as log2-transformed, where value 0 means 'no change' and 1 means doubled value and -1 means halved value. The values are always in relation to the group used as a base level group (reference).

p-value P-value describes the reliability of the change in expression value between the compared groups. Better (i.e. smaller) p-value is given for those genes that show homogeneous behaviour inside each group and yet clearly differ between the compared groups. In this analysis the P-values used for filtering can be either so called modified t-test p-values or *FDR* (*false-discovery-rate*) p-values which are both produced by Limma (see B). Modified t-test p-values are not corrected for multiple testing. FDR p-values are used to control the rate of false positive findings in the result list and have been generally found to perform better than traditional p-values.

Choosing thresholds for filtering

The choice of the thresholds for p-value and fold change used for filtering the differentially expressed (DE) genes is not a trivial task. There is no one correct way or method to determine

the thresholds but the choice is based on different aspects of each study. Different thresholds can also be used for filtering the data for different purposes. For example, often very strict thresholds are chosen when the data is filtered to be included in a publication. Then the result list will contain very few false positive findings but on the other hand many true positives are left outside the result set. Because of this it is typically useful to use less stringent thresholds for filtering data for internal research purposes or functional analysis when a larger proportion of possible false positive findings can be tolerated. Cluster analysis of the filtered genes can also be used as a means for choosing the filtering thresholds: such thresholds should be chosen, that the samples are grouping according to the known sample groups in the cluster analysis of the filtered genes.

Thresholds used in filtering the DE genes and the corresponding numbers of results:

The tables below shows the numbers of filtered genes and thresholds used for each comparison. The thresholds were chosen to list a reasonable number of the most differentially expressed genes. With the chosen thresholds the filtered genes clustered according to the sample groups giving evidence of condition dependent behavior of the selected genes.

Comparison	FC	logFC	PType	P	Tot	Up	Down
nAT rescue vs. WT	2.000	1.000	FDR	0.050	139	83	56
dBAH rescue vs. nAT rescue	1.500	0.580	P.Val	0.010	53	21	32
dBAHmut1 rescue vs. dBAH rescue	1.500	0.580	P.Val	0.010	97	37	60

Table 4: Filtering summary table

Comparison	FC	logFC	PType	P	Tot	Up	Down
dBAH vs. WT	2.000	1.000	FDR	0.050	156	95	61
dBAHmut1 vs. WT	2.000	1.000	FDR	0.050	186	105	81
dBAHmut1 vs. nAT	1.500	0.580	P.Val	0.010	89	29	60

Table 5: Filtering summary table

5.2 Filtering result files

Up-regulated targets in each comparison can be found in file 'upRegulatedGenes<comparisonName>.xls' and down-regulated targets in file 'downRegulatedGenes<comparisonName>.xls'.

Result files for each comparison 'annotatedData<comparisonName>.xls' include results from the statistical testing as well as the annotations for all targets on the array. These files can be used to check results for genes not appearing in the lists of differentially expressed targets.

In these files only the group mean intensities and standard deviations are reported. The normalised intensities for individual samples can be found from the file 'NormalizedData.xls' file by the corresponding target identifiers. The unnormalised intensities can be found in the file 'RawData.xls'.

The results are organised by average ranking (based both on p-value and fold change), in decreasing order of significance. As p-values alone ignore the size of the gene expression change and fold changes alone ignore the consistency of the changes, ordering the genes in the result files based only on one of these two is not the best approach. Thus we have developed a method based on average ranking for ordering the genes which is more likely to capture the true biological order of importance. The method works as follows: First the genes inside each comparison are ranked independently based on p-values (increasing order) and absolute fold changes (decreasing order). Next the average ranks for these two are calculated. As these values do not follow the original value range, these average ranks are further ranked again to get the ranks from 1 to the number of genes, rank of 1 meaning the highest possibble rank (most differentially expressed gene based both on p-value and fold change). In tie situations, the order of the results is randomized.

5.3 Visualization of the DE features

Volcano plot(s)

The following figure(s) show the results of the comparison(s) as Volcano plot(s).

In a Volcano plot the log10 of the p-values is on the y-axis and the logFC calculated for the comparison group vs. base level group is on the x-axis. In this plot it can be seen how the reliability values of the measurement features behave in relation to the fold change. The thresholds used in filtering are marked in the plot with tickled lines, up-regulated genes are coloured red and down-regulated genes green.

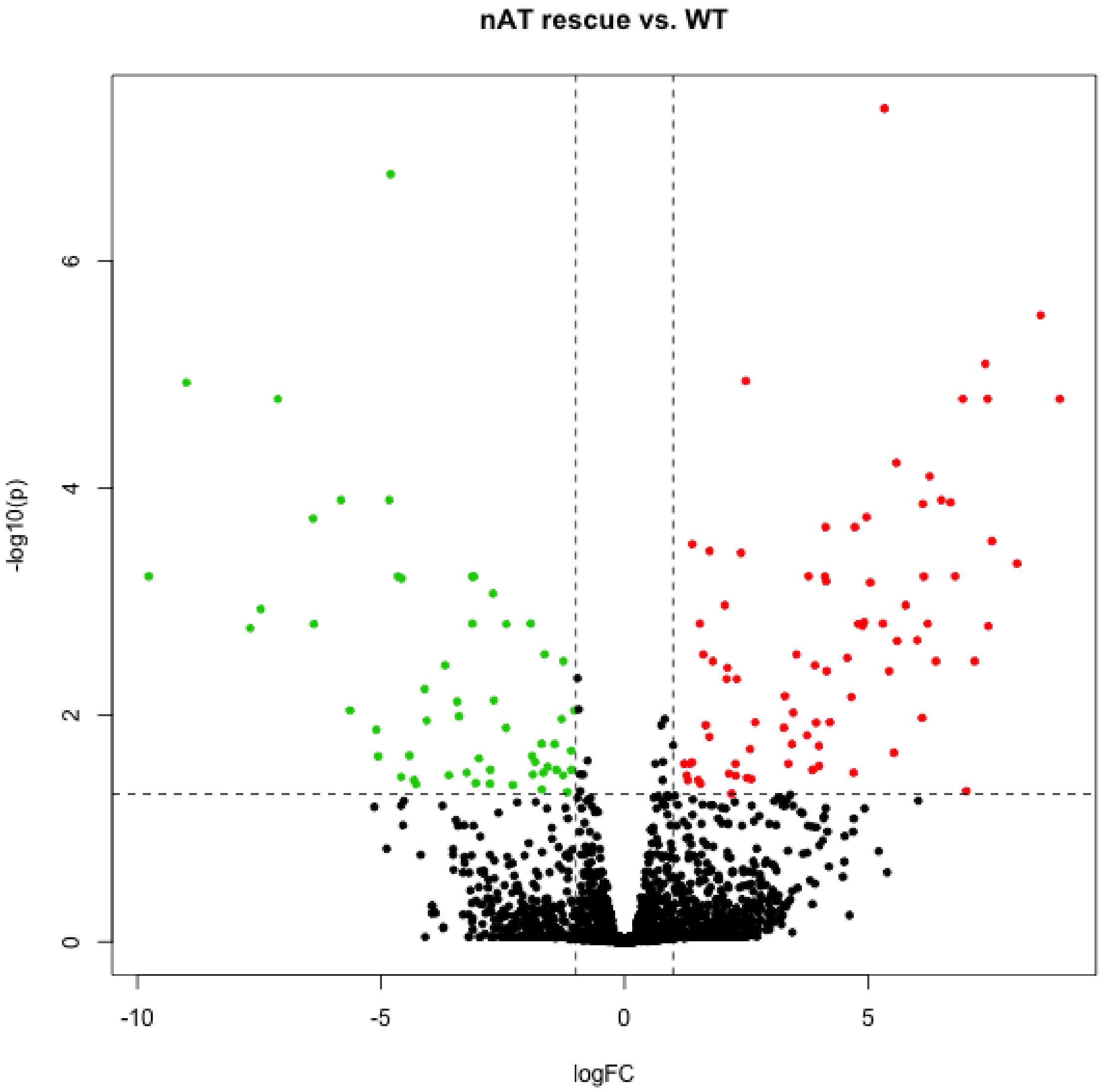

Figure 9: VOLCANO plot for comparison nAT rescue vs. WT.

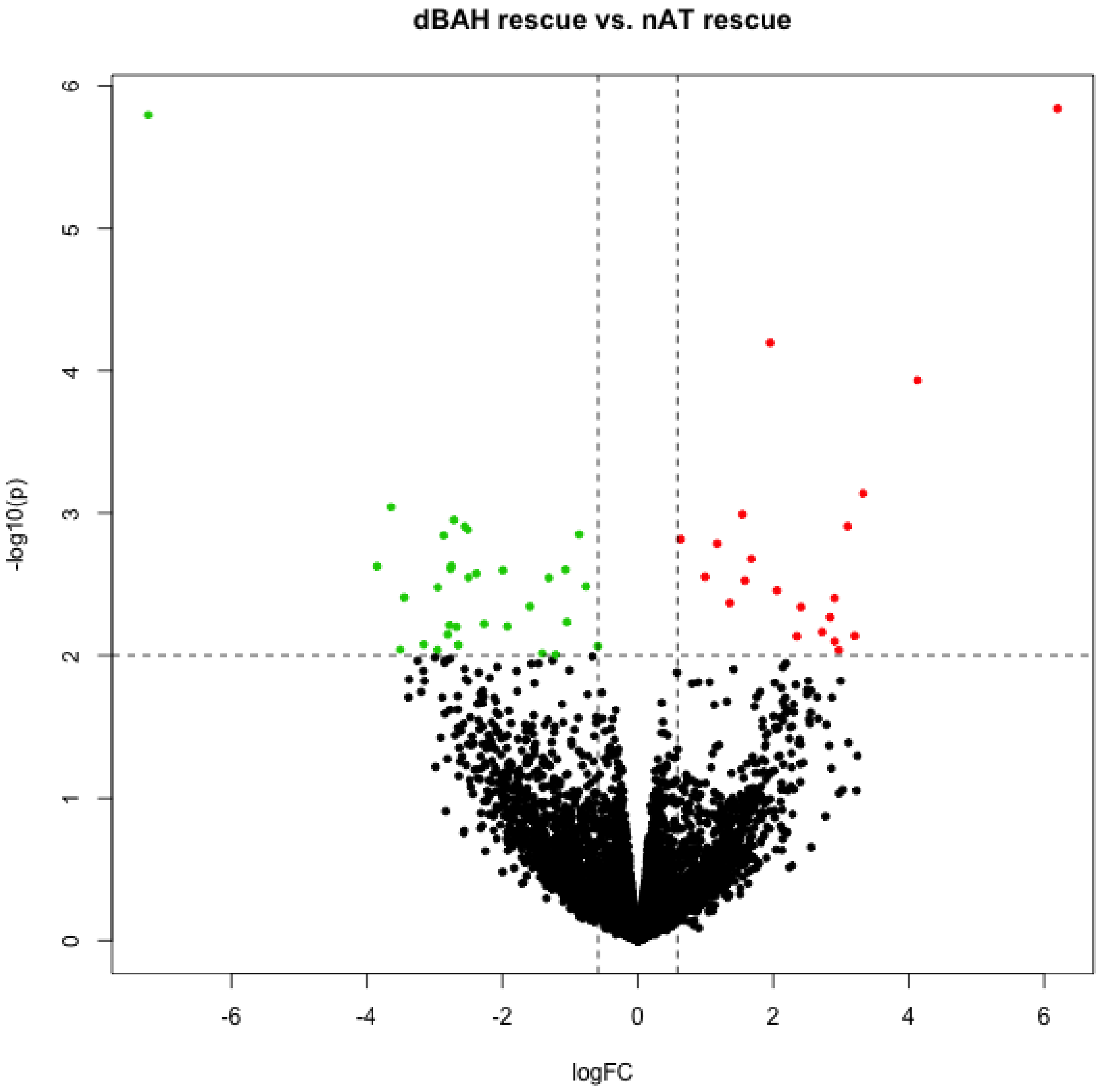

Figure 10: VOLCANO plot for comparison dBAH rescue vs. nAT rescue.

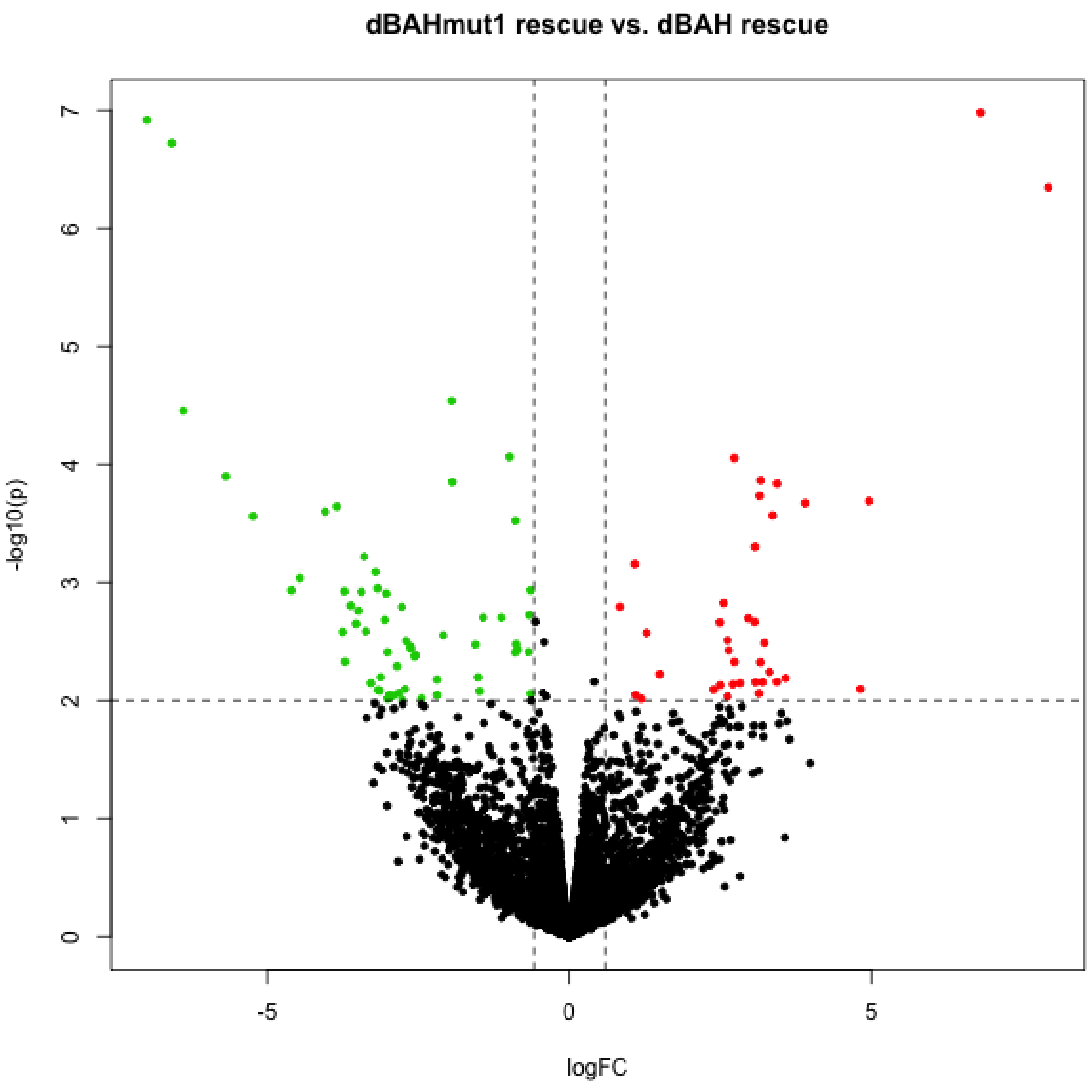

Figure 11: VOLCANO plot for comparison dBAH mut 1 rescue vs. dBAH rescue.

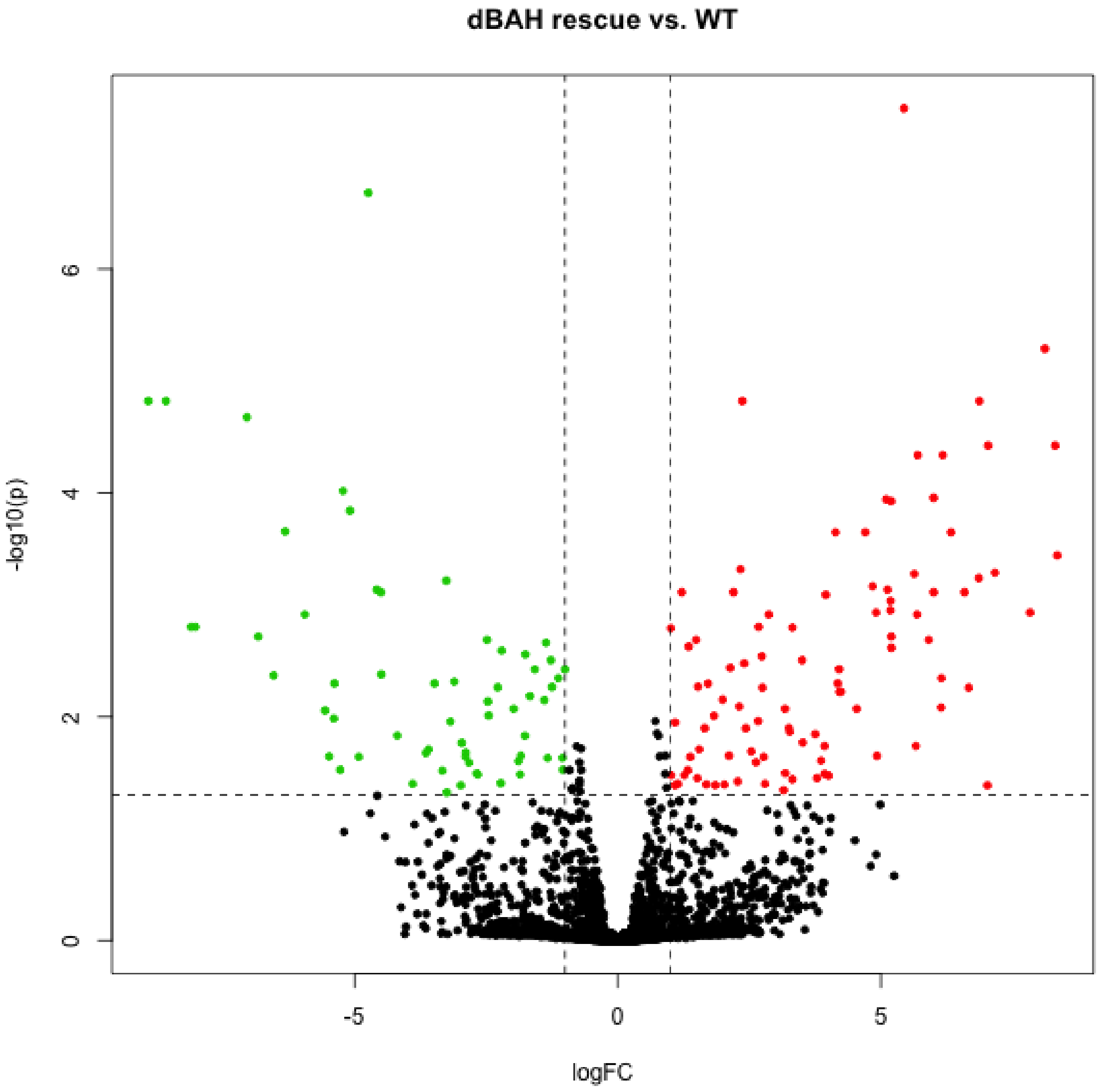

Figure 12: VOLCANO plot for comparison dBAH rescue vs. WT.

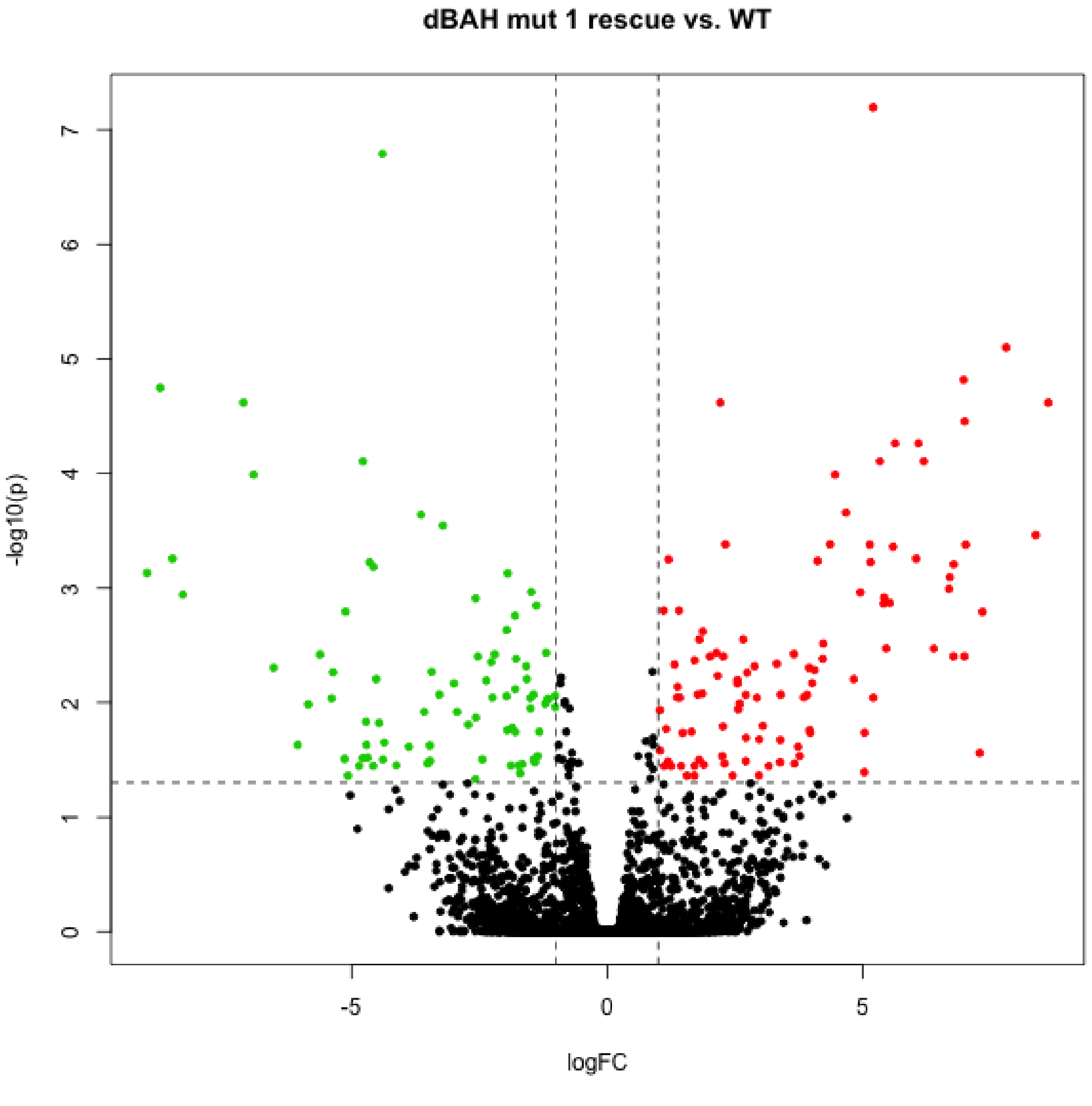

Figure 13: VOLCANO plot for comparison dBAH mut 1 rescue vs. WT.

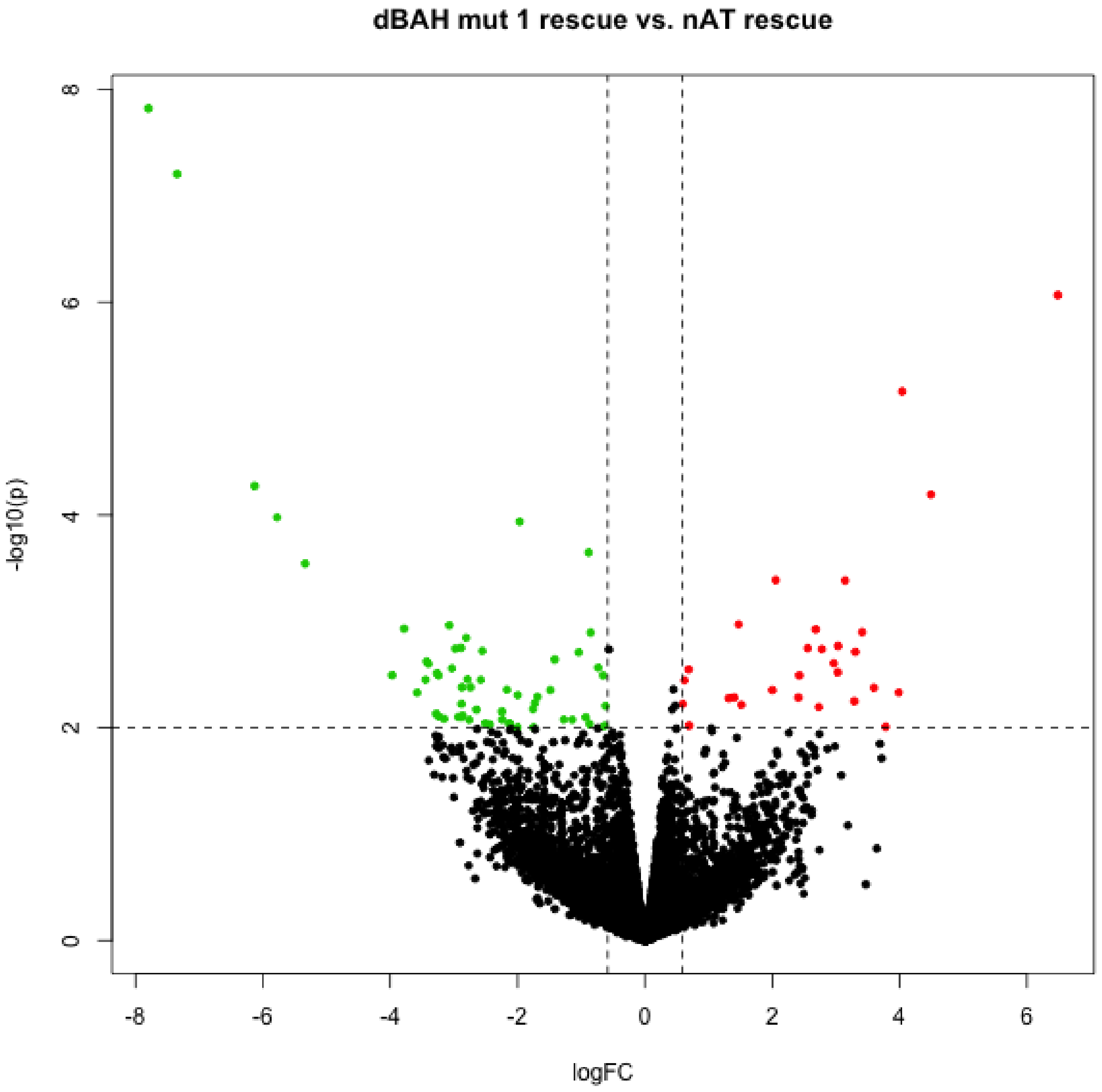

Figure 14: VOLCANO plot for comparison dBAH mut 1 rescue vs. nAT rescue.

Heatmap(s)

The following figure(s) show the heat map clustering of the differentially expressed features for the comparison(s). Pearson's metrics has been used in hierarchical clustering of the samples and filtered features. The clustering is based on the general expression measurement similarity. In the plot red colour means high expression and green low expression. Each row represents one DE feature and each column represents one sample.

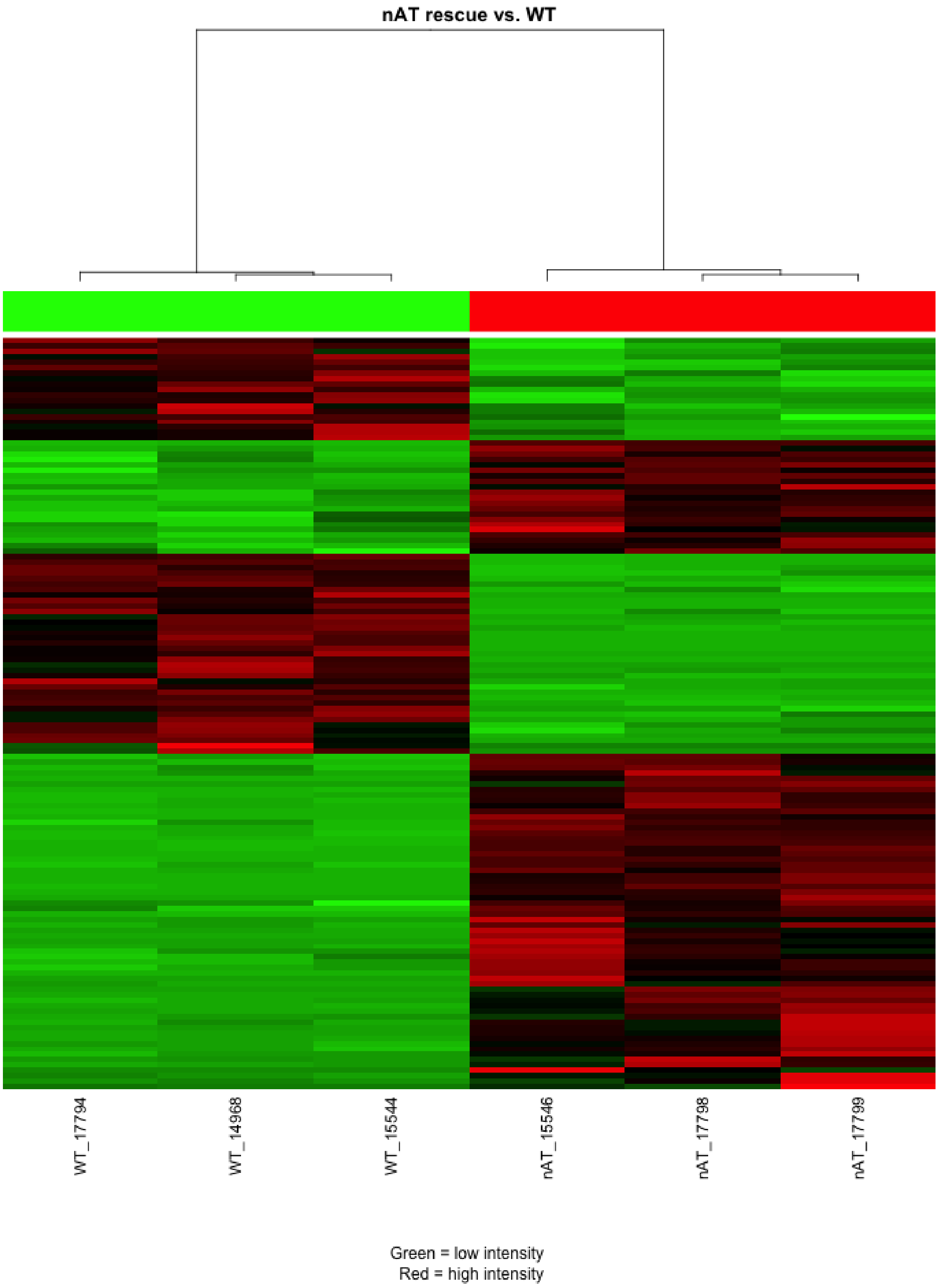

Figure 15: HEATMAP plot for comparison nAT rescue vs. WT.

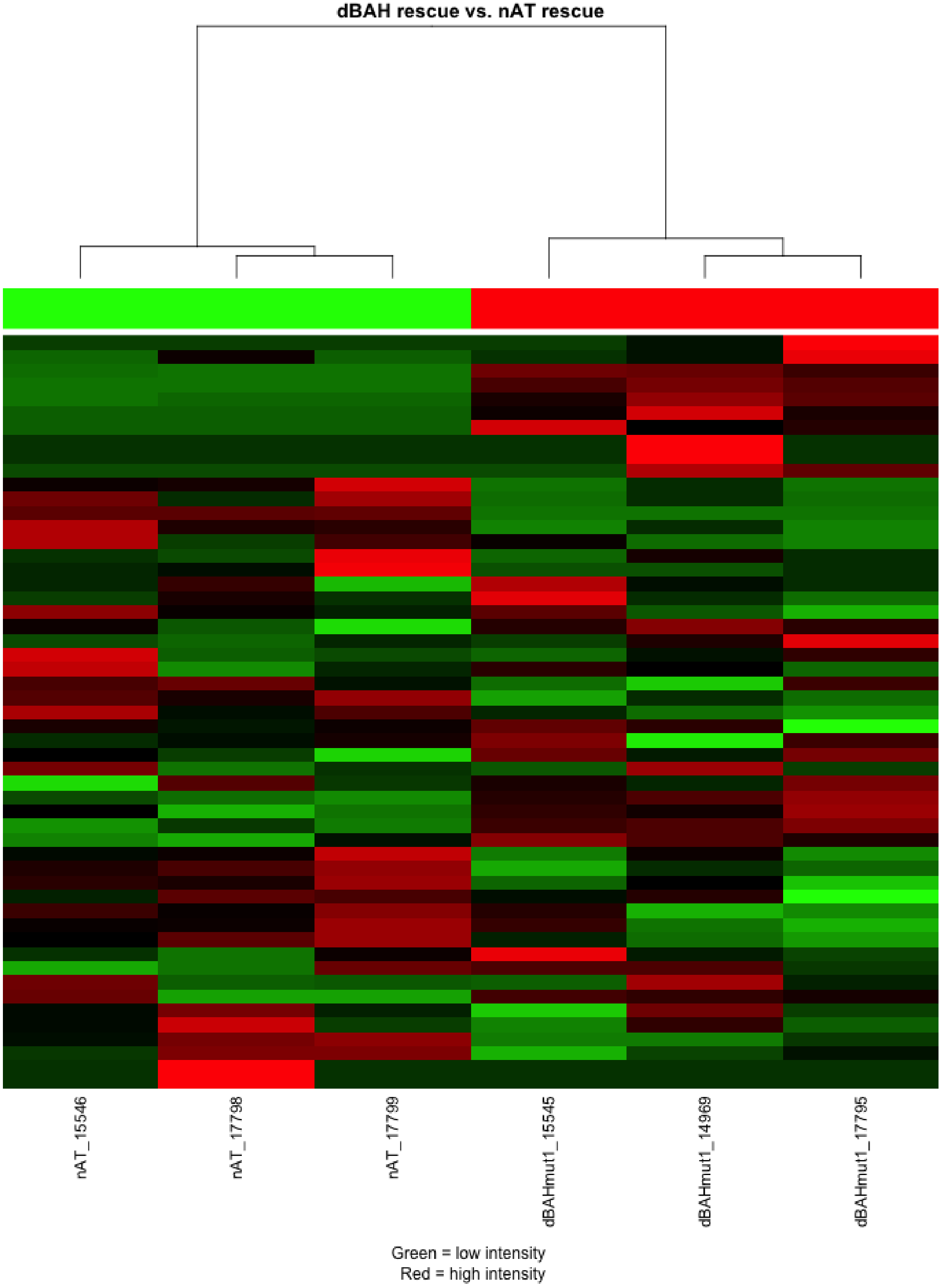

Figure 16: HEATMAP plot for comparison dBAH rescue vs. nAT rescue.

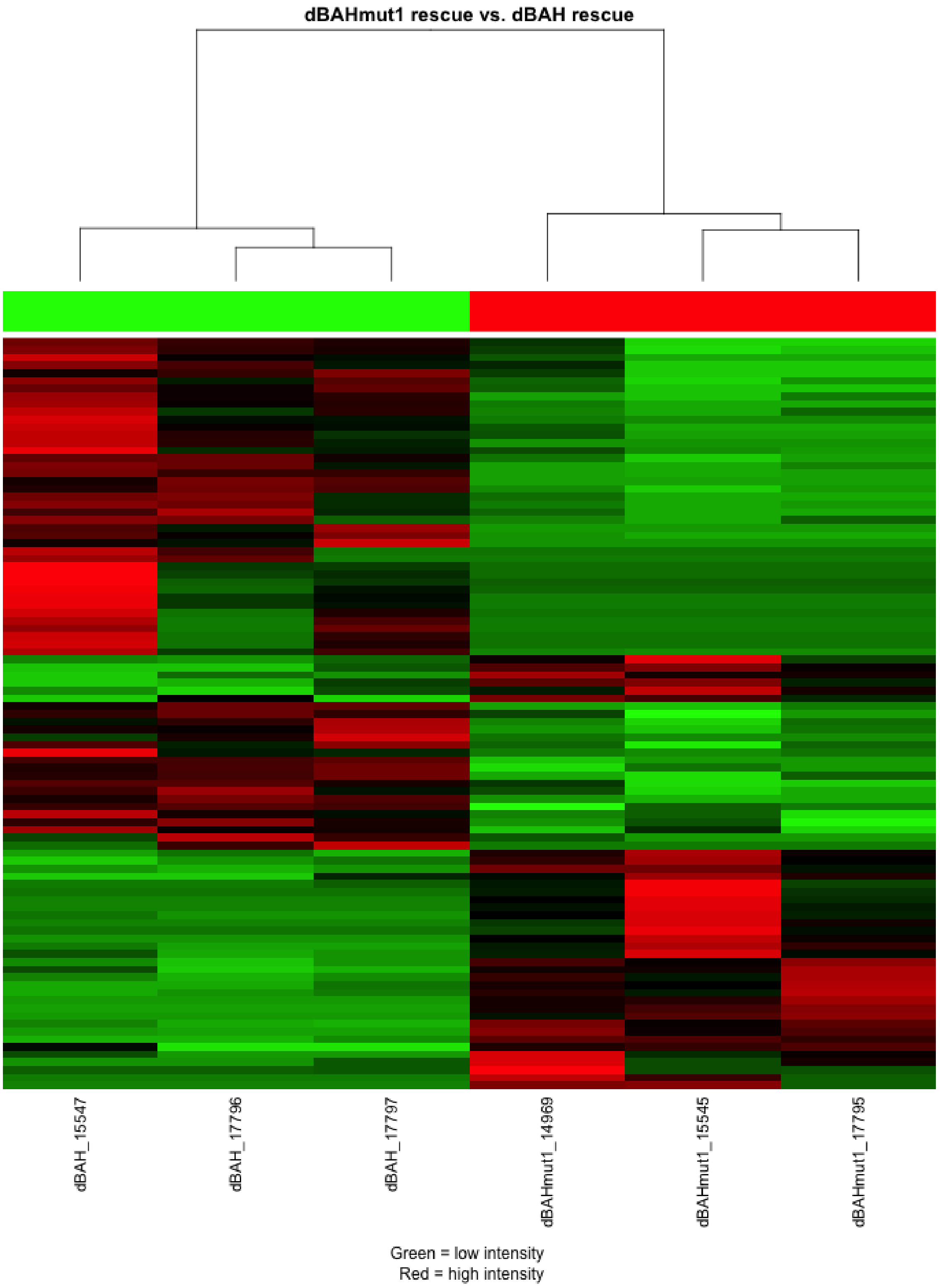

Figure 17: HEATMAP plot for comparison dBAH mut 1 rescue vs. dBAH rescue.

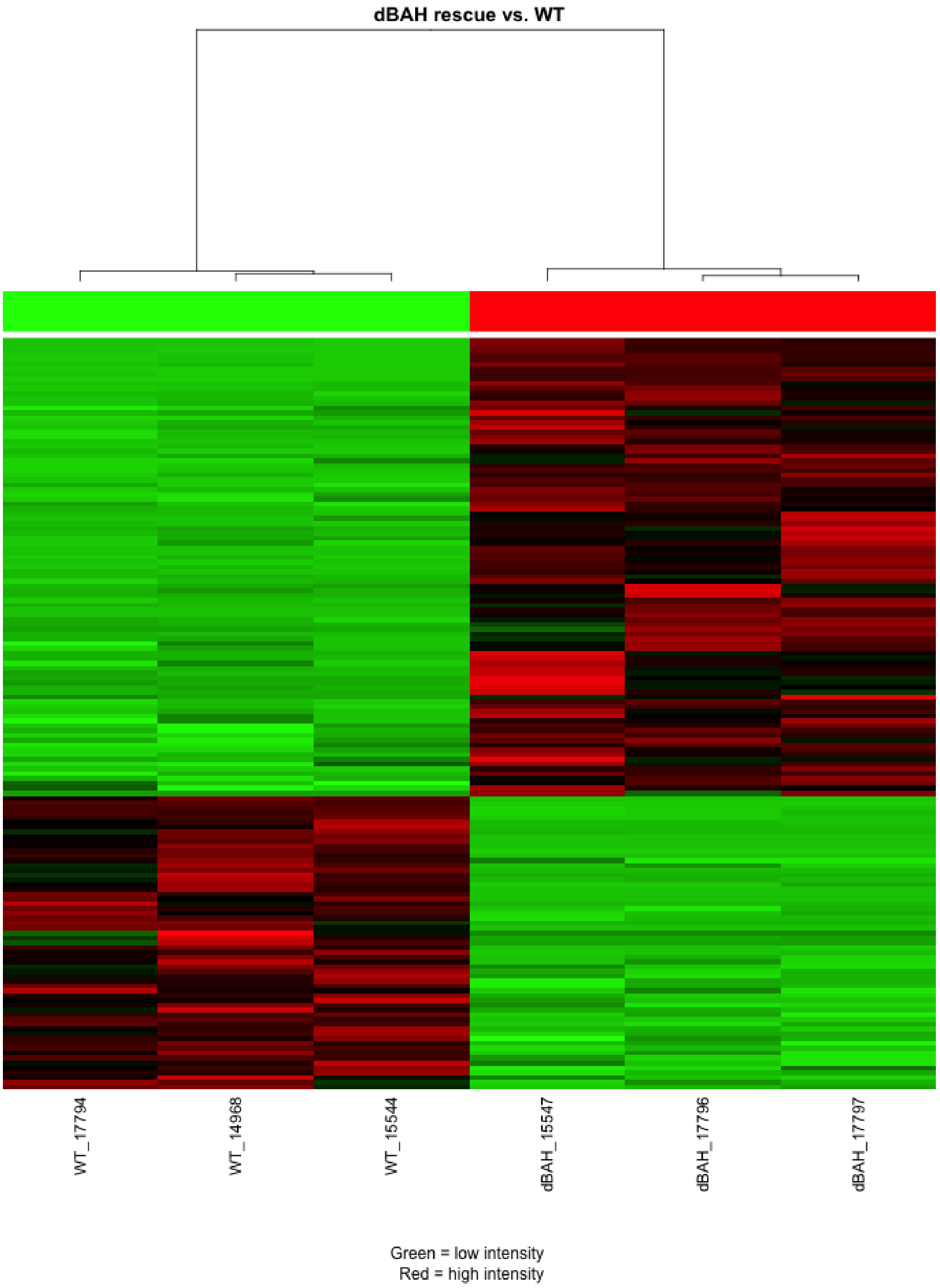

Figure 18: HEATMAP plot for comparison dBAH rescue vs. WT.

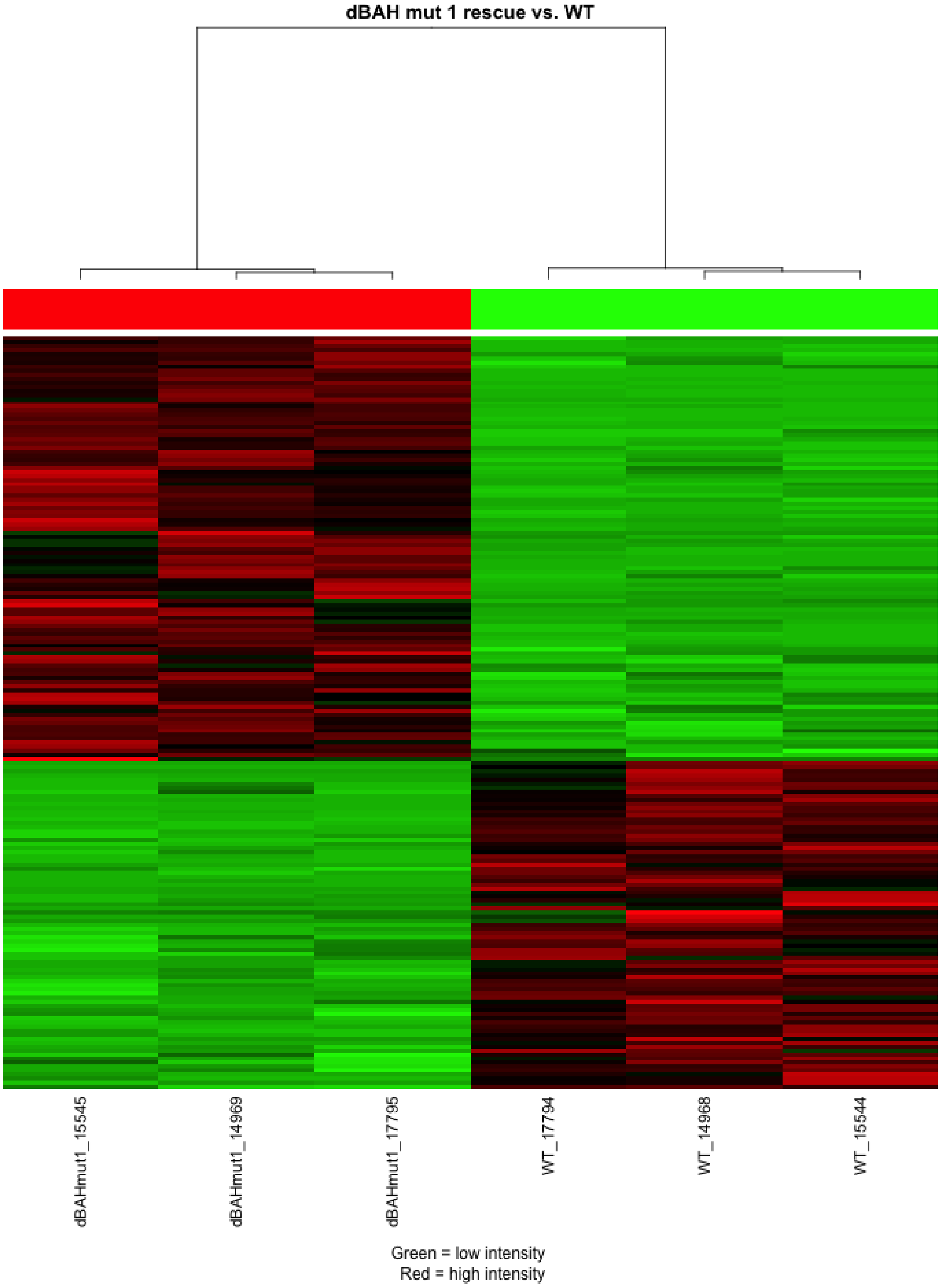

Figure 19: HEATMAP plot for comparison dBAH mut 1 rescue vs. WT.

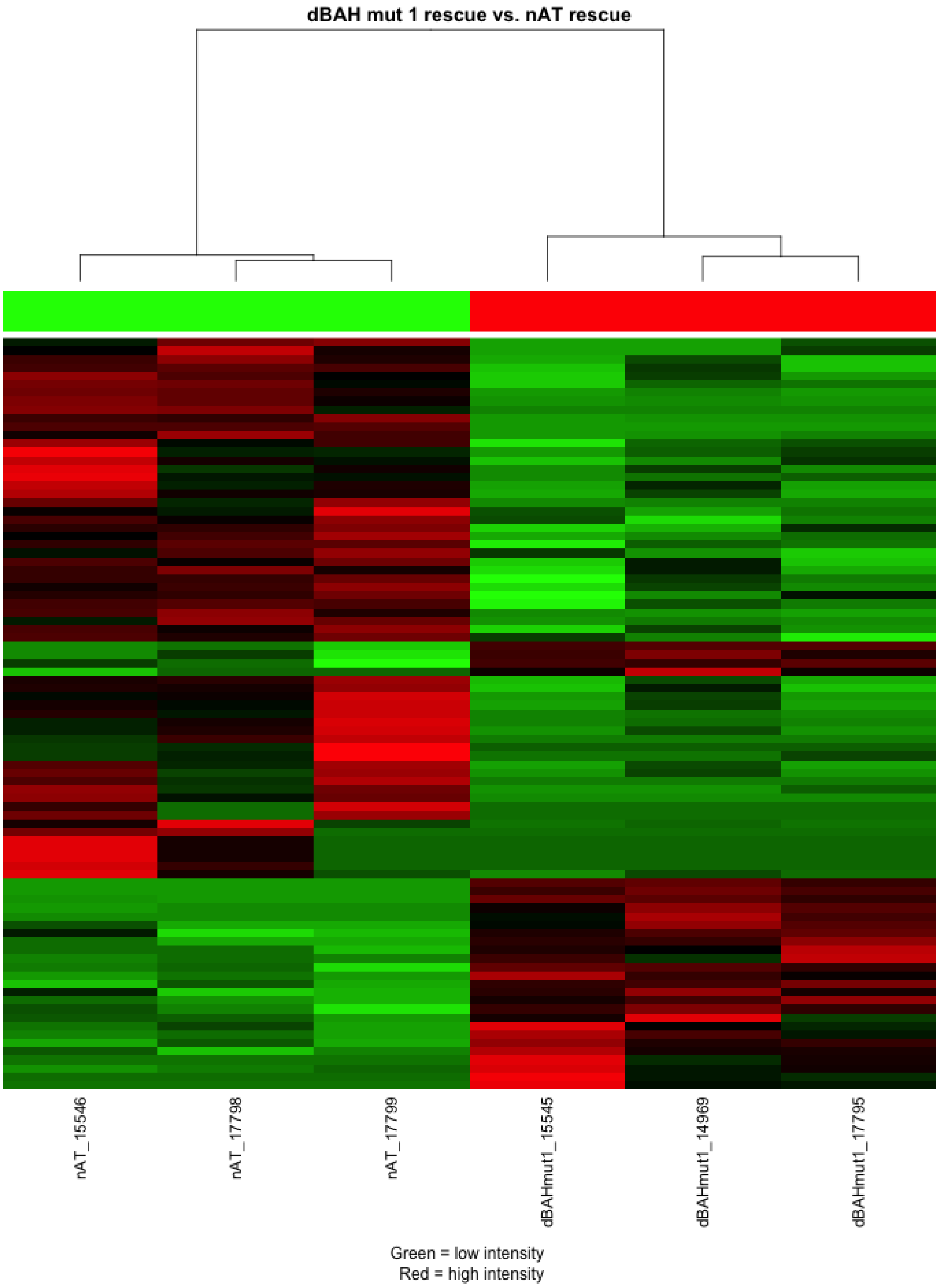

Figure 20: HEATMAP plot for comparison dBAH mut 1 rescue vs. nAT rescue.

PCA plot(s)

The PCA analysis results of the differentially expressed features in the comparison(s) are presented in following figure(s).

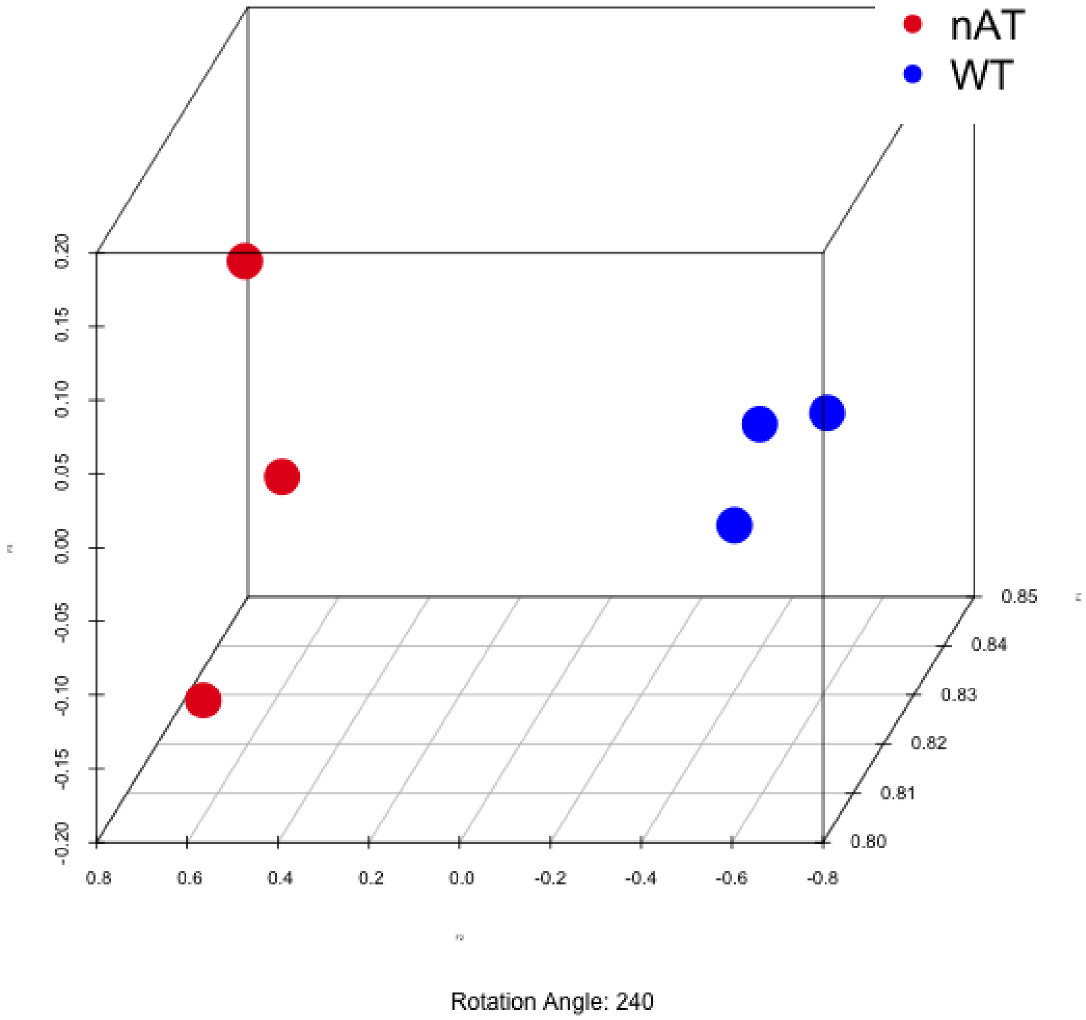

Figure 21: PCA plot for comparison nAT rescue vs. WT.

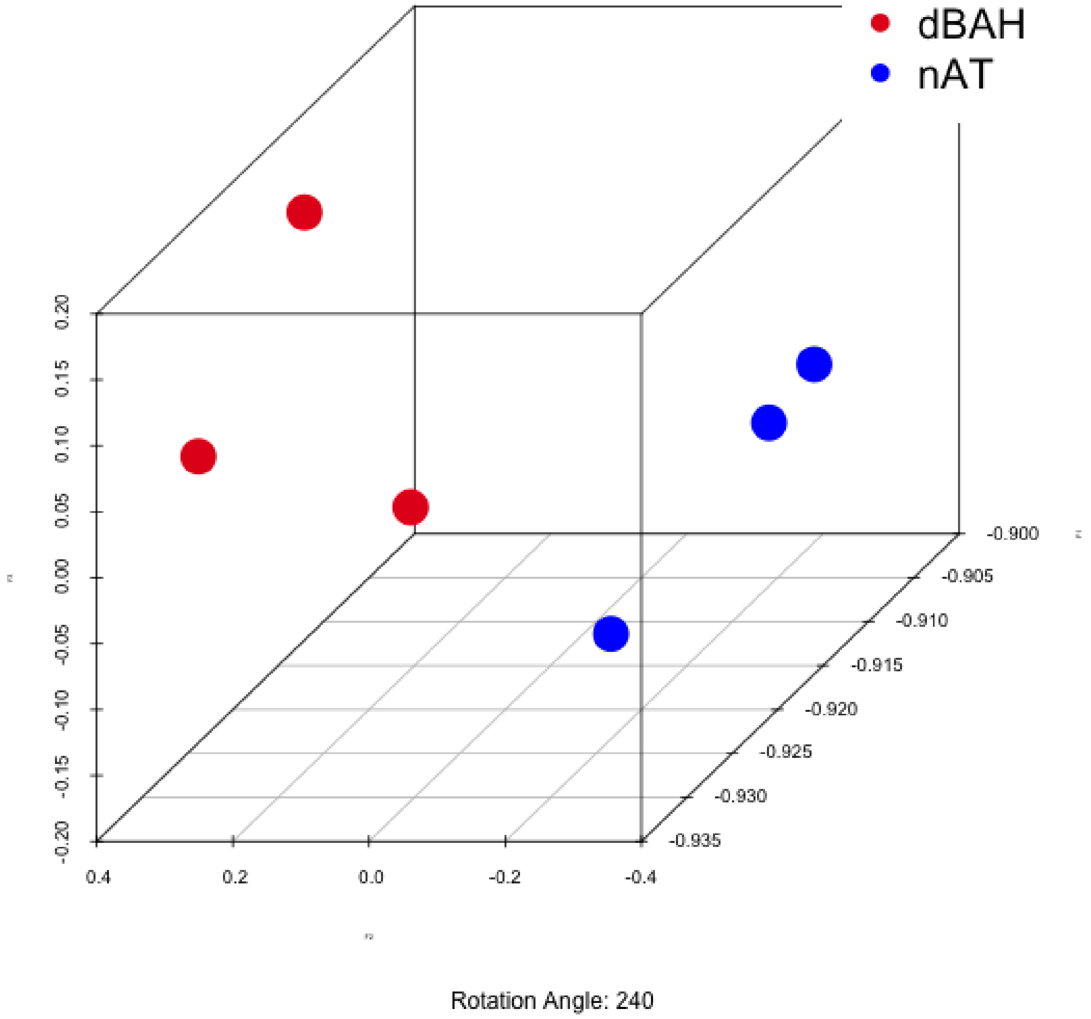

Figure 22: PCA plot for comparison dBAH rescue vs. nAT rescue.

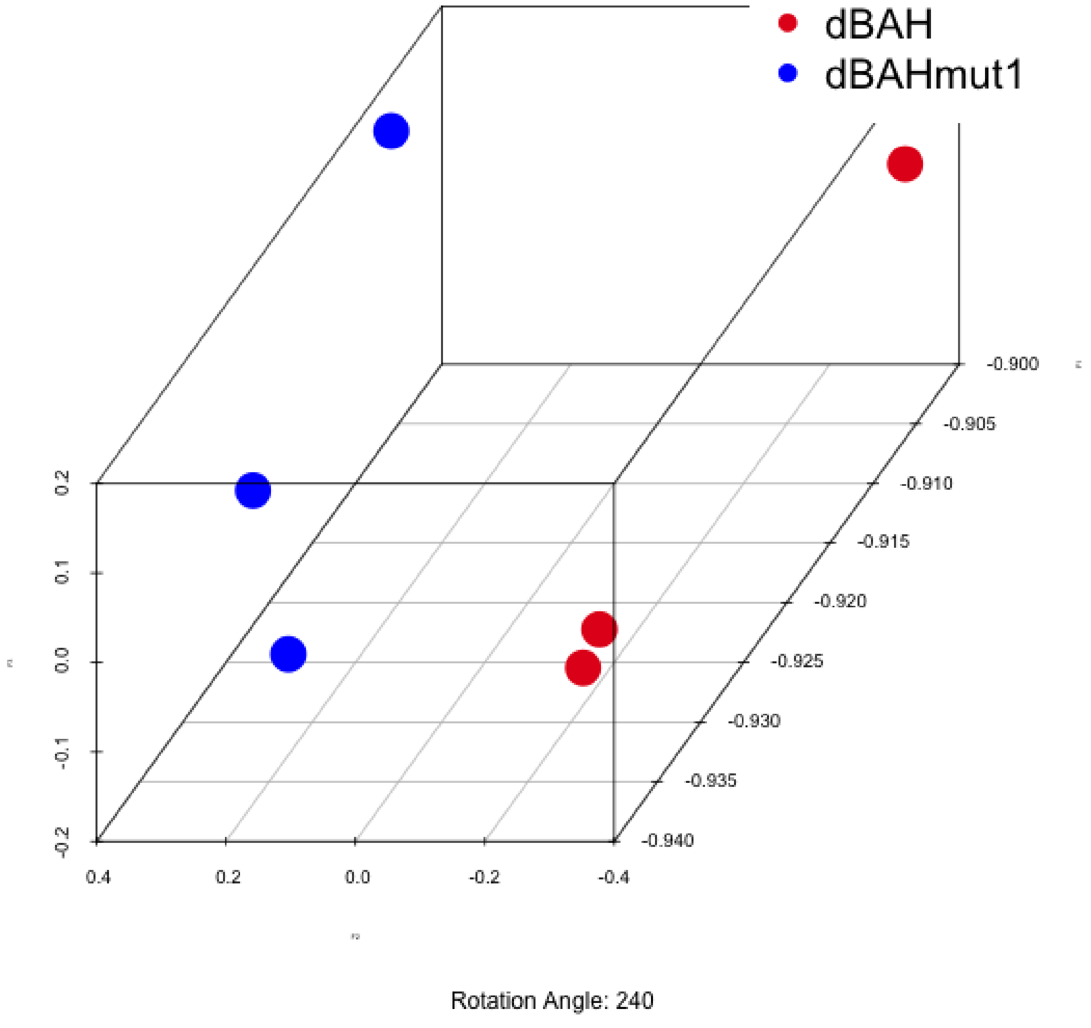

Figure 23: PCA plot for comparison dBAH mut 1 rescue vs. dBAH rescue

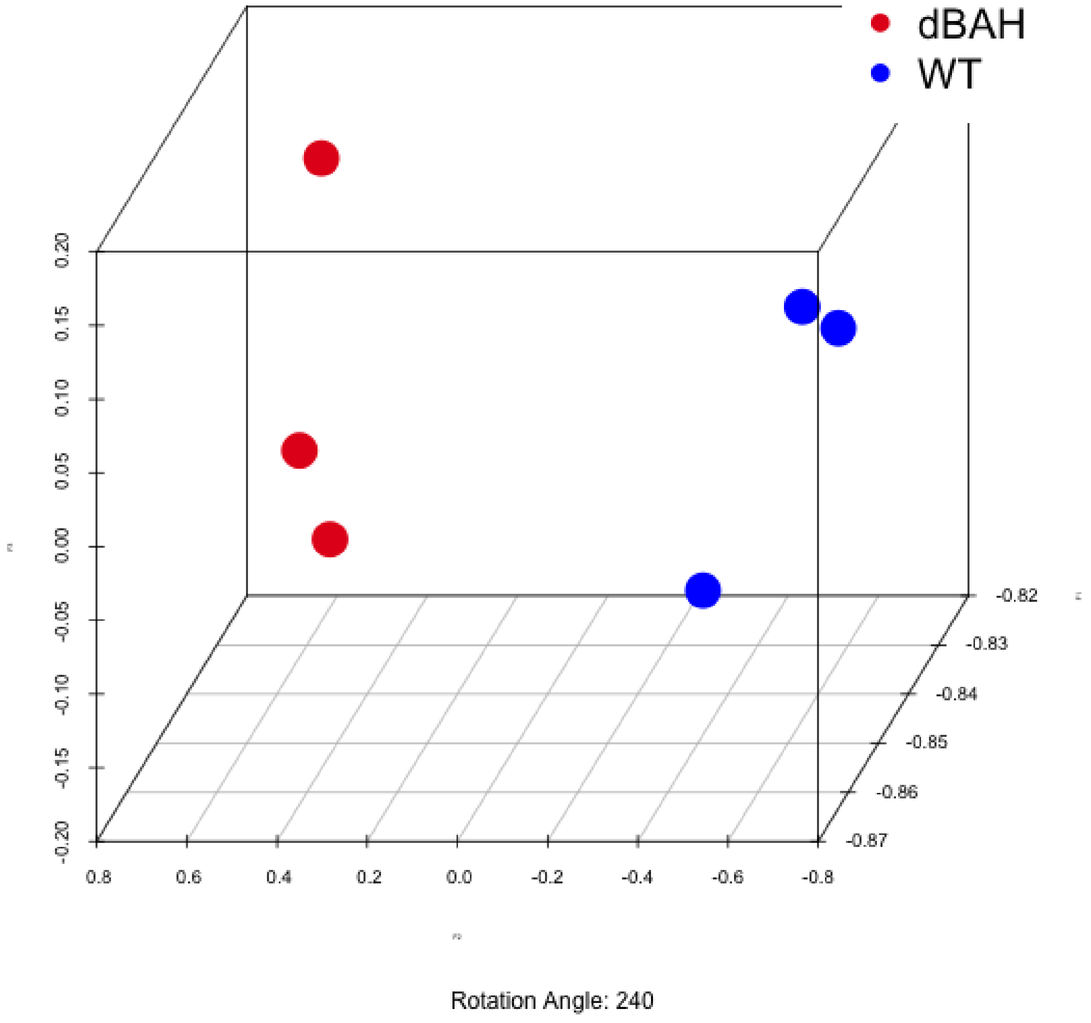

Figure 24: PCA plot for comparison dBAH rescue vs. WT

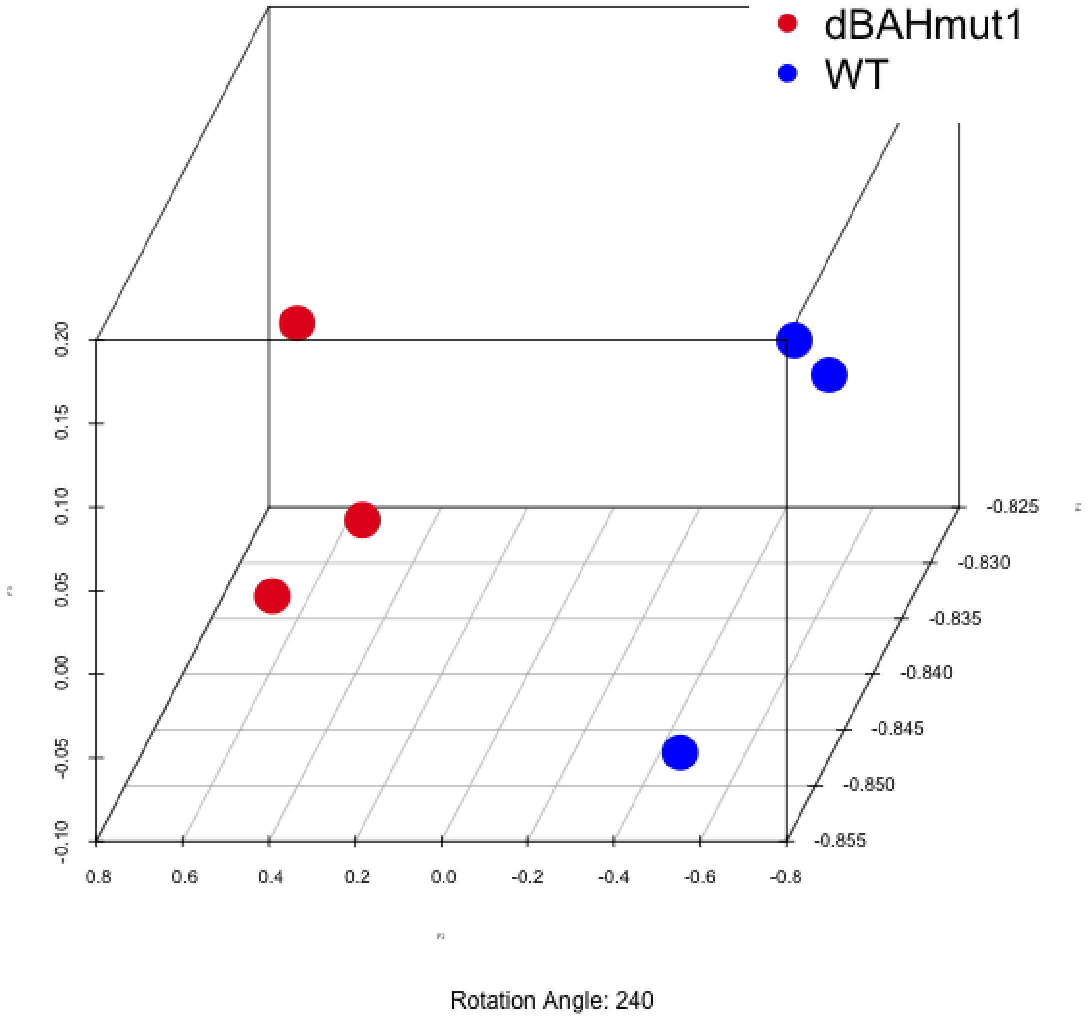

Figure 25: PCA plot for comparison dBAH mut 1 rescue vs. WT

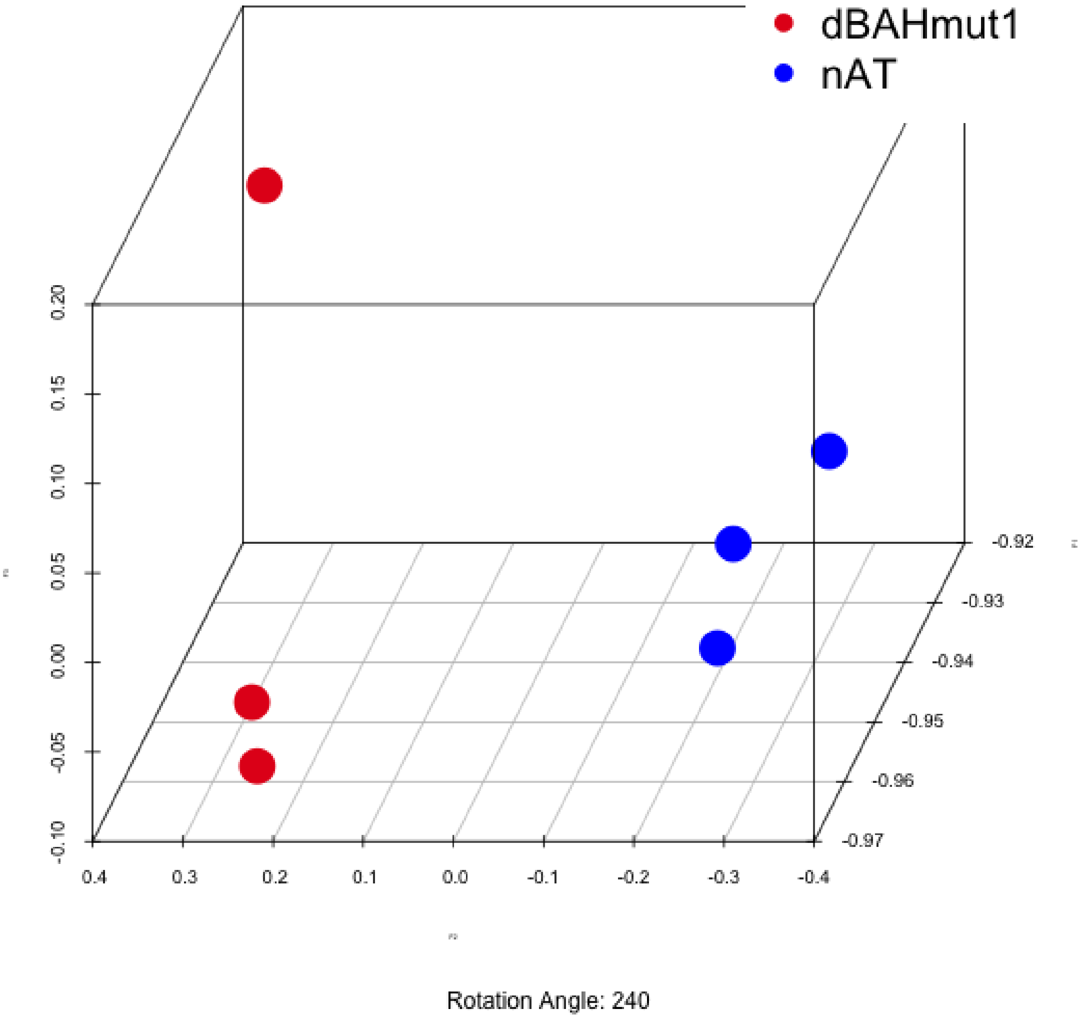

Figure 26: PCA plot for comparison dBAH mut 1 rescue vs. nAT rescue
